# Fish oil and aspirin effects on arteriovenous fistula function: Secondary outcomes of the randomised omega-3 fatty acids (Fish oils) and Aspirin in Vascular access OUtcomes in REnal Disease (FAVOURED) trial

**DOI:** 10.1371/journal.pone.0213274

**Published:** 2019-03-26

**Authors:** Andrea K. Viecelli, Kevan R. Polkinghorne, Elaine M. Pascoe, Peta-Anne Paul-Brent, Carmel M. Hawley, Sunil V. Badve, Alan Cass, Lai-Seong Hooi, Peter G. Kerr, Trevor A. Mori, Loke-Meng Ong, David Voss, David W. Johnson, Ashley B. Irish, for the Omega-3 Fatty Acids (Fish Oils) and Aspirin in Vascular Access Outcomes in Renal Disease (FAVOURED) Study Collaborative Group

**Affiliations:** 1 Department of Nephrology, Princess Alexandra Hospital, Brisbane, Australia; 2 Australasian Kidney Trials Network, Faculty of Medicine, University of Queensland, Brisbane, Australia; 3 Department of Nephrology, Monash Medical Centre, Melbourne, Australia; 4 Department of Medicine, Monash University, Melbourne, Australia; 5 School of Public Health and Preventive Medicine, Monash University, Melbourne, Australia; 6 Translational Research Institute, Brisbane, Australia; 7 Department of Nephrology, St George Hospital, Sydney, Australia; 8 The George Institute for Global Health, Sydney, Australia; 9 Menzies School of Health Research, Charles Darwin University, Darwin, Australia; 10 Department of Medicine and Hemodialysis Unit, Hospital Sultanah Aminah, Johor Bahru, Malaysia; 11 Medical School, University of Western Australia, Perth, Australia; 12 Department of Nephrology, Penang Hospital, Georgetown, Malaysia; 13 Middlemore Renal Services, Middlemore Hospital, Auckland, New Zealand; 14 Department of Nephrology, Fiona Stanley Hospital, Perth, Australia; Public Library of Science, UNITED KINGDOM

## Abstract

**Background:**

Arteriovenous fistulas (AVF) for haemodialysis often experience early thrombosis and maturation failure requiring intervention and/or central venous catheter (CVC) placement. This secondary and exploratory analysis of the FAVOURED study determined whether omega-3 fatty acids (fish oils) or aspirin affected AVF usability, intervention rates and CVC requirements.

**Methods:**

In 567 adult participants planned for AVF creation, all were randomised to fish oil (4g/d) or placebo, and 406 to aspirin (100mg/d) or placebo, starting one day pre-surgery and continued for three months. Outcomes evaluated within 12 months included AVF intervention rates, CVC exposure, late dialysis suitability failure, and times to primary patency loss, abandonment and successful cannulation.

**Results:**

Final analyses included 536 participants randomised to fish oil or placebo (mean age 55 years, 64% male, 45% diabetic) and 388 randomised to aspirin or placebo. Compared with placebo, fish oil reduced intervention rates (0.82 vs 1.14/1000 patient-days, incidence rate ratio [IRR] 0.72, 95% confidence interval [CI] 0.54–0.97), particularly interventions for acute thrombosis (0.09 vs 0.17/1000 patient-days, IRR 0.53, 95% CI 0.34–0.84). Aspirin significantly reduced rescue intervention rates (IRR 0.45, 95% CI 0.27–0.78). Neither agent significantly affected CVC exposure, late dialysis suitability failure or time to primary patency loss, AVF abandonment or successful cannulation.

**Conclusion:**

Although fish oil and low-dose aspirin given for 3 months reduced intervention rates in newly created AVF, they had no significant effects on CVC exposure, AVF usability and time to primary patency loss or access abandonment. Reduction in access interventions benefits patients, reduces costs and warrants further study.

## Introduction

A functioning vascular access is essential for patients requiring haemodialysis (HD). A native arteriovenous fistula (AVF) has the best long-term outcomes although this advantage is frequently limited by early thrombosis, maturation failure, the need for access interventions and/or placement of a central venous catheter (CVC)[1,2]. Vascular access interventions are burdensome for patients and incur significant health care costs[3]. Patients and health professionals consider the need for interventions to maintain the use of a vascular access for HD the most important adverse outcome of a vascular access[4], yet treatments to reduce intervention rates and to increase the usability of AVF have not been a major focus of randomised trials in patients requiring HD[5]. The inhibitory effects of omega-3 polyunsaturated fatty acids (fish oils) on platelet aggregation[6,7], inflammation[8,9], and neointimal hyperplasia[10], and of aspirin on platelet inhibition could be beneficial in reducing the need for interventions for acute thrombosis and maturation-enhancing procedures. Fish oil supplementation has been shown to reduce intervention and thrombosis rates in arteriovenous grafts[11] but has not previously been studied in AVF.

The omega-3 fatty acids (Fish oils) and Aspirin in Vascular access OUtcomes in REnal Disease (FAVOURED) trial found that neither fish oil nor aspirin reduced the primary outcome ‘AVF failure’, a binary composite outcome comprising AVF thrombosis and/or AVF abandonment and/or cannulation failure assessed at 12 months following AVF creation[12]. The current analysis of secondary and exploratory outcomes of the FAVOURED trial aimed to determine whether fish oil or low-dose aspirin could reduce the need for access interventions and CVC exposure and/or increase the usability of newly created AVF for HD.

## Materials and methods

### Design and population

The design and results of the main outcomes of the FAVOURED study have been published[12–16] and the study protocol and statistical analysis plan are provided in Supporting Information S1 and S2 Files, respectively. FAVOURED was a prospective, double-blind, randomised controlled trial conducted in Australia, New Zealand, the United Kingdom and Malaysia involving 567 adults with stage 4 or 5 chronic kidney disease who were receiving or expected to receive HD within 12 months and scheduled for AVF surgery. The original study protocol underwent two amendments after study initiation[14]: First, the primary outcome of “early thrombosis” within 12 weeks of AVF creation was broadened to the clinically important outcome of “AVF access failure”, a composite of thrombosis and/or AVF abandonment and/or cannulation failure at 12 months. Second, the exclusion criteria of aspirin use were removed to allow participants on medically indicated aspirin to be randomised to fish oil or matching placebo and to continue using an open-label aspirin. Participants were randomly allocated in a 1:1 ratio to receive either fish oil (4g/d, 46% eicosapentaenoic acid and 38% docosahexaenoic acid) or matching placebo (olive oil). A subset of 406 participants either not taking aspirin or able to cease it prior to enrolment were further randomised in a 1:1 ratio to receive 100mg of oral aspirin daily or matching placebo. Treatment commenced one day prior to surgery and continued for 12 weeks. Participants were randomised via a central, web-based system (Flexetrials) using an adaptive minimisation algorithm with study site and planned AVF location (upper versus lower arm) as minimisation variables. Participants and care providers, laboratory staff and members of the study team were blinded to treatment allocation. Ethics approval for the Omega-3 fatty acids (Fish Oils) and Aspirin in Vascular access OUtcomes in REnal Disease (FAVOURED) trial was obtained from local Human Research Ethics Committees (HREC) in all participating centres prior to study initiation and participant enrolment. Approving HRECs included: *Australia*—Austin Health HREC, Cairns & Hinterland Health Service District HREC, Ethics Review Committee (RPAH Zone), South Metropolitan Area Health Service HREC, Barwon Health HREC, Gold Coast Health Service District HREC, Greenslopes Private Hospital Ethics Committee, Tasmanian Health and Medical HREC, Southern Health HREC C, Metro South HREC, Royal Adelaide Hospital Research Ethics Committee, Melbourne Health HREC, Royal Perth Hospital HREC, Sir Charles Gairdner Hospital HREC, Sydney Adventist Hospital HREC, Sir Charles Gairdner Hospital HREC, Sydney Adventist Hospital HREC, The Alfred Ethics Committee, Australian Capital Territory Health HREC, Ethics of Health Research Committee (TQEH & LMH), Darling downs–West Moreton (Toowoomba & Darling Downs) Health Service District HREC, Townsville Health Service District HREC, The University of Queensland Medical Research Ethics Committee; *New Zealand*—Multi-region Ethics Committee; *Malaysia*—Ministry of Health Malaysia Medical Research & Ethics Committee; and the *United Kingdom*—National Research Ethics Service Nottingham Research Ethics Committee. The study was performed in accordance with the Declaration of Helsinki and written consent was obtained from all participants. The study was terminated early because of slower than anticipated recruitment, funding issues and lack of ongoing availability of trial medications. Two interim efficacy analyses using the Haybittle-Peto rule were planned after one-third and two-thirds of recruited patients with at least 12 months follow-up, but only the first interim analysis was conducted due to early trial cessation. FAVOURED was registered with the Australia & New Zealand Clinical Trials Register (ACTRN12607000569404).

### Outcomes

Pre-specified secondary[12] and exploratory outcomes included the number and type of interventions from AVF creation to 12 months, and the time to the first intervention. Interventions comprised *rescue* procedures designed to restore patency of the AVF (medical thrombolysis or surgical thrombectomy) and *non-rescue* procedures (surgical or radiological revision or dilation of the AVF from or proximal to the anastomosis to the ipsilateral central vein, dilation of central venous stenosis, ligation of tributaries, superficialisation of the AVF, ligation of the AVF or salvage by distal reconstruction and interval ligation due to distal ischemia). Additional secondary outcomes encompassed the time to first successful cannulation (the time between surgery and first of three consecutive successful cannulations), time to primary patency loss (first thrombosis or need for rescue intervention), time to permanent AVF abandonment, time to abandonment or primary patency loss, and CVC exposure for HD (binary and count [duration in situ] outcome). Exploratory analyses included the binary outcomes of primary patency loss within the first 12 months and late dialysis suitability failure[17] (inability to cannulate the study AVF for at least 8 out of 12 consecutive HD sessions or access abandonment by 6 months post-surgery). S1 Table provides a summary of the outcomes and definitions.

### Statistical analysis

Continuous variables were expressed as mean [± standard deviation] or median [interquartile range] depending on the distribution and categorical variables as numbers and percentages. Pearson’s Chi-square test or Fisher’s exact test were used to compare categorical data as appropriate. Treatment effects for binary outcomes were determined by log-binomial regression and expressed as relative risks (RR) and 95% confidence interval (CI). Incidence rate ratios from Poisson regression were used for treatment comparisons on count outcomes. Cox proportional sub-distribution hazards models were used to compare treatment effects on time to first intervention, first successful cannulation and AVF abandonment treating death and transplantation as competing events. The proportional hazards assumption was tested by adding an interaction between treatment and time to each model. As there were few competing risks (<1% to 2.4%), survival results were displayed as Kaplan-Meier curves with 95% CI and competing events censored. All outcome comparisons of fish oil with placebo were adjusted for differences in aspirin use (randomised to aspirin, randomised to placebo aspirin, open-label aspirin). The robustness of the fish oil effect was assessed by additional analyses that adjusted for pre-specified baseline characteristics (planned AVF site, diabetes mellitus, age, cardiovascular comorbidities, and renal replacement therapy at baseline) and study region (Australia and New Zealand, Malaysia and the United Kingdom). The same statistical methods were used for the comparison of aspirin with matching placebo. A two-sided p-value less than 0.05 was considered statistically significant. Statistical analyses were performed using SAS version 9.4 (SAS Institute).

## Results

**Fig 1:**
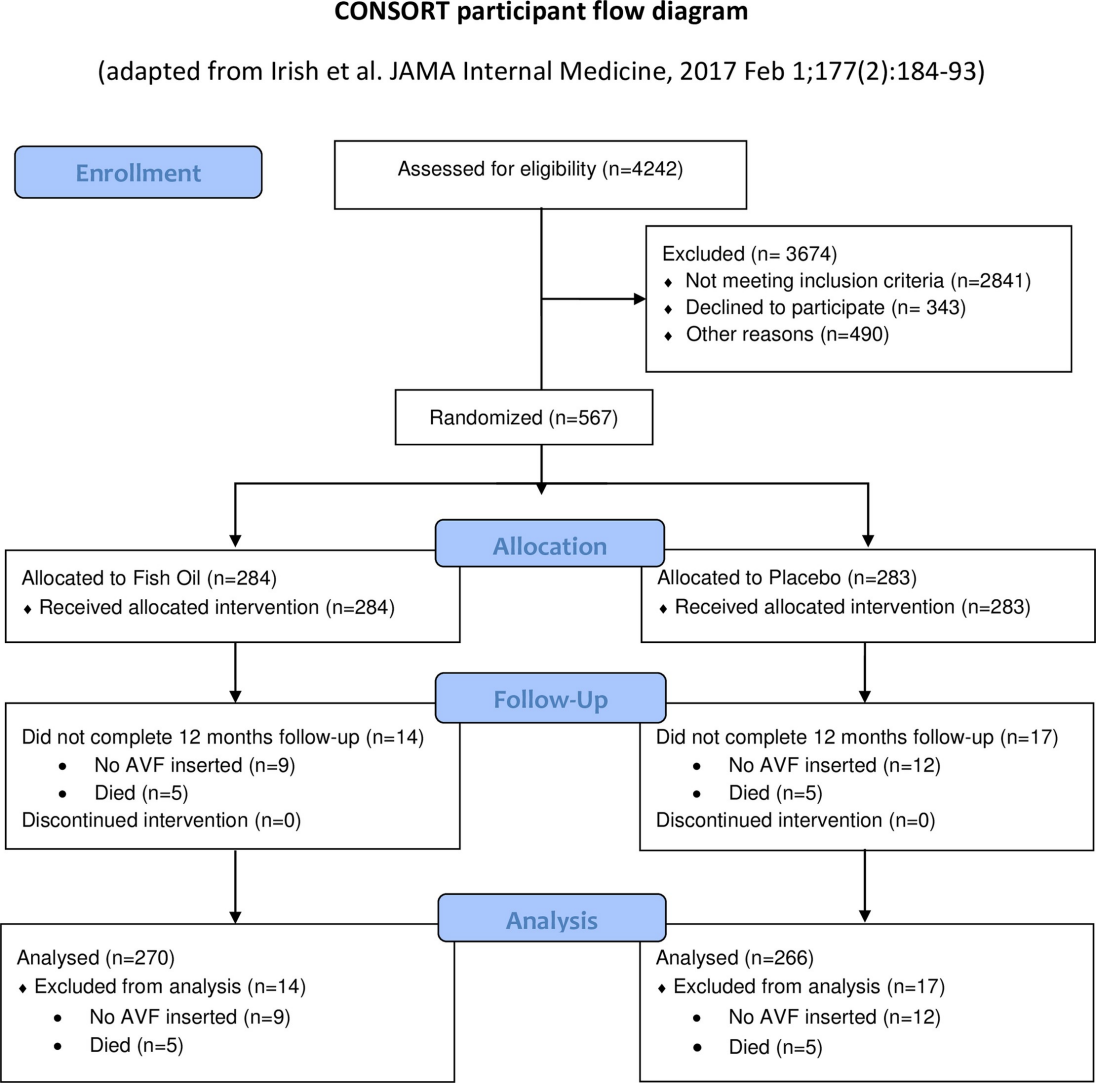
CONSORT participant flow diagram.

The FAVOURED study randomised 567 participants to fish oil or placebo from August 21, 2008 to February 28, 2014, of which 536 were included in the final analysis[12]Fig 1. Table 1 shows the baseline characteristics of the 536 participants; 31 participants were excluded because they either died prior to being assessed on any outcome (n = 5 in each of the fish oil and placebo groups) or did not have an AVF created (n = 9 randomised to fish oil, n = 12 randomised to placebo)[12]. Participants had a mean age of 55 years and 64% were male. Baseline characteristics were generally well balanced although more participants treated with fish oil compared to placebo were diabetic (48% versus 43%) or smokers (53% versus 48%). At study initiation, 49% were on dialysis with 84% dialysing through a CVC. At study end, 83% received dialysis with 61% using the study AVF (167 [62%] randomised to fish oil, 159 [60%] randomised to placebo).

**Table 1:** Patient demographics and baseline characteristics for fish oil versus placebo and separately for the subset of aspirin versus placebo.

Characteristics	Fish oil (n = 270)	Placebo (n = 266)	Aspirin (n = 194)	Placebo (n = 194)
Age, (years, mean ± SD)	54.2 ± 14.1	55.9 ± 14.7	52.4 ± 14.6	54.2 ± 14.9
Male, n (%)	171 (63)	171 (64)	120 (62)	126 (65)
Country, n (%)				
Australia and New Zealand	192 (71)	191 (72)	150 (74)	147 (72)
Malaysia	75 (28)	69 (26)	53 (26)	53 (26)
United Kingdom	3 (1)	6 (2)	0	3 (2)
Ethnicity, n (%)				
Asian	88 (33)	81 (31)	66 (34)	56 (29)
White	139 (52)	150 (56)	102 (53)	117 (60)
Indigenous^a^	34 (13)	25 (9)	22 (11)	15 (8)
Other	9 (3)	10 (4)	4 (2)	6 (3)
Body mass index, (kg/m^2^; mean ± SD)	28.8 ± 7.4	28.3 ± 7.0	27.7 ± 6.6	28.4 ± 7.7
Blood pressure, (mm Hg; mean ± SD)				
Systolic	146 ± 23	146 ± 23	145 ± 22	146 ± 24
Diastolic	82 ± 14	81 ± 13	81 ± 13	83 ± 14
Comorbid conditions				
Diabetes mellitus, n (%)	130 (48)	113 (43)	79 (41)	70 (36)
Cardiovascular disease, n (%)	39 (14)	40 (15)	14 (7)	10 (5)
Hypertension, n (%)	234 (87)	241 (91)	174 (90)	172 (89)
Current or prior smoking, n (%)	144 (53)	128 (48)	92 (47)	93 (48)
Medications				
Aspirin, n (%)	71 (26)	72 (27)	NA	NA
Statin, n (%)	143 (53)	132 (50)	79 (41)	87 (45)
ESA, n (%)	119 (44)	134 (50)	99 (51)	90 (46)
Beta-blocker, n (%)	123 (46)	124 (47)	91 (47)	77 (40)
ARB/ACEI, n (%)	108 (40)	116 (44)	85 (44)	80 (41)
CCB, n (%)	150 (56)	149 (56)	113 (58)	103 (53)
Planned study AVF location, n (%)^b^				
Upper arm	104 (39)	103 (39)	84 (41)	82 (40)
Forearm	166 (62)	163 (61)	119 (59)	121 (60)
Renal replacement therapy at time of AVF creation, n (%)				
Peritoneal dialysis	14 (5)	19 (7)	16 (8)	10 (5)
Haemodialysis	115 (43)	111 (42)	79 (41)	88 (45)
Not currently receiving dialysis	141 (52)	136 (51)	99 (51)	96 (50)
Principal access currently in use for participants receiving dialysis, n (%)				
AVF	5 (4)	2 (2)	2 (2)	1 (1)
AVG	0	1 (1)	0	0
CVC (cuffed and non-cuffed)	110 (85)	108 (83)	77 (81)	87 (89)
Peritoneal dialysis catheter	14 (11)	19 (15)	16 (17)	10 (10)
Dialysis duration (months; median [IQR])^c^	3.8 [1.8, 18.2]	4.2 [1.9, 16.1]	4.6 (2.0, 16.3)	4.0 (1.7, 15.1)

^a^Aboriginal, Torres Strait Islanders, Maori and Pacific Islanders

^b^Actual AVF location upper arm versus forearm: fish oil n = 110 (41%) versus n = 160 (59%); placebo n = 107 (40%) versus n = 159 (60%); aspirin n = 79 (41%) versus n = 115 (59%); placebo n = 79 (41%) versus n = 115 (59%).

^c^For pre-dialysis participants only (fish oil n = 141 and placebo n = 136; aspirin n = 99 and placebo n = 96)

Abbreviations: ACEI–angiotensin-converting enzyme inhibitor; AVF–arteriovenous fistula; ARB–angiotensin receptor blocker; CCB–calcium channel blocker; CVC–Central venous catheter; eGFR–estimated glomerular filtration rate; ESAs–erythropoietin stimulating agents; IQR–interquartile range; N/A–not applicable; SD–standard deviation.

Of the 406 participants not taking aspirin or able to cease it prior to enrolment, 203 were randomised to aspirin and 203 to matching placebo[12] of which 388 (194 in each group) were included in the analysis (S1 Fig). The remaining 18 participants were excluded because they either did not undergo AVF creation (n = 5 randomised to aspirin, n = 8 randomised to placebo) or died before they could be assessed on any outcome (n = 4 randomised to aspirin, n = 1 randomised to placebo). As outlined in Table 1, this subset of 388 participants had a lower prevalence of cardiovascular disease (7% randomised to aspirin, 5% randomised to placebo) compared to the full set of 536 participants randomised to fish oil (14%) or matching placebo (15%).

### AVF interventions

**Fig 2:**
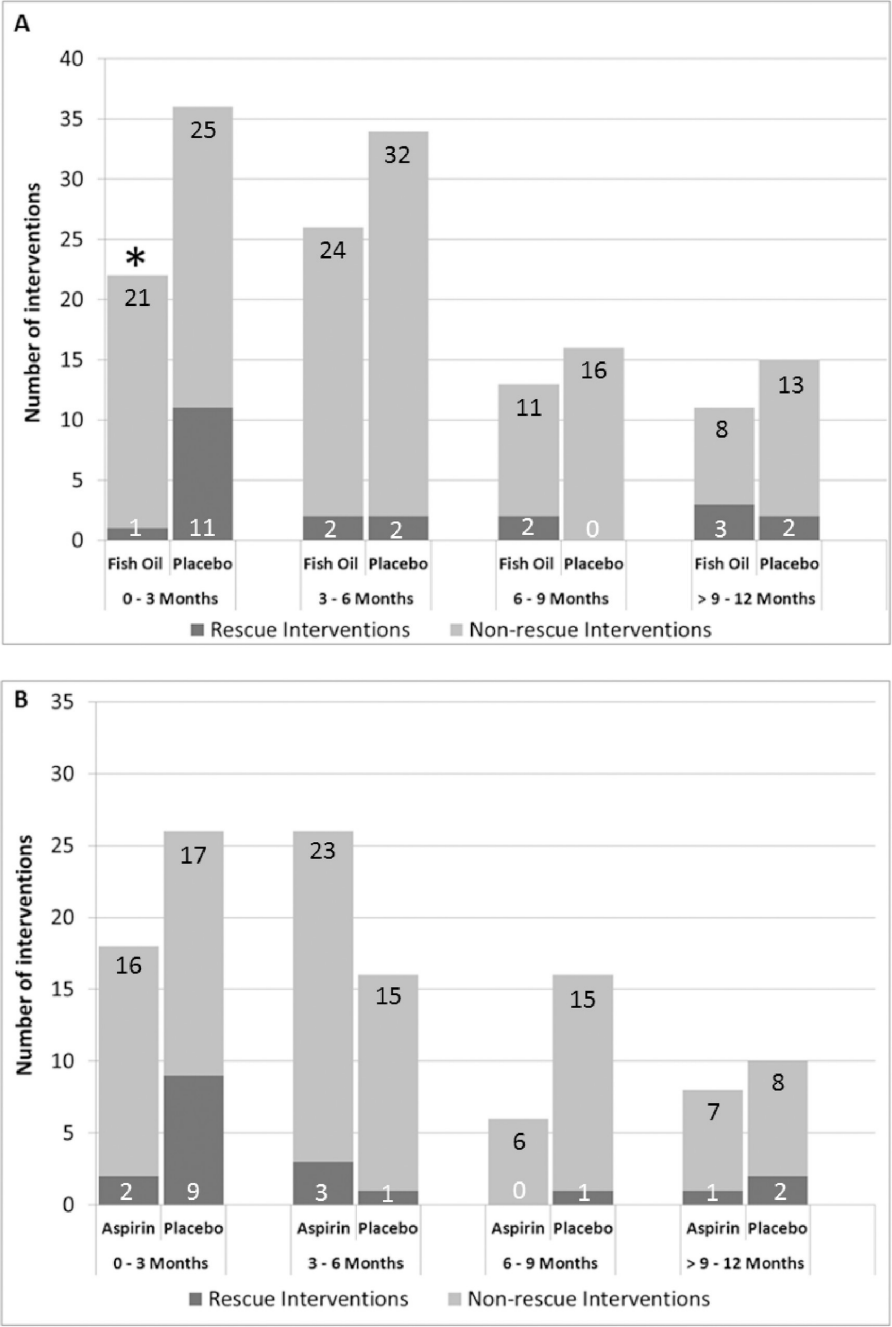
**Number of rescue- and non-rescue interventions by time period for fish oil versus placebo (A) and aspirin versus placebo (B).** *p = 0.009 (Fisher’s Exact Test) for comparison of rescue intervention during first 3 months (active treatment phase) and beyond 3 months.

**Fig 3:**
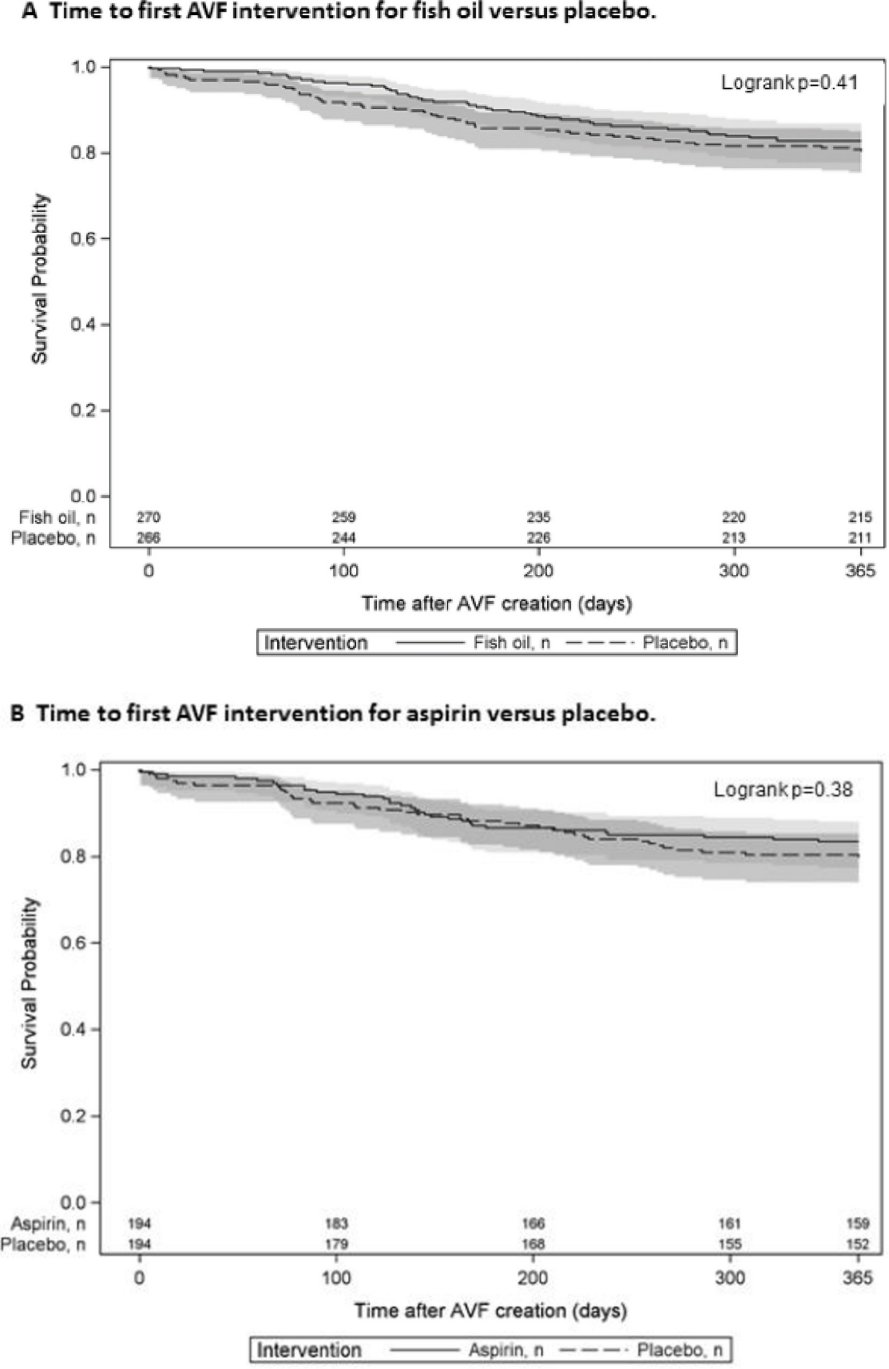
**Time to first AVF intervention for fish oil versus placebo (A) and aspirin versus placebo (B).** Kaplan-Meier curve for active therapy (solid line with 95% confidence interval in light gray) and matching placebo (dashed line with 95% confidence interval in dark gray).

**Fig 4:**
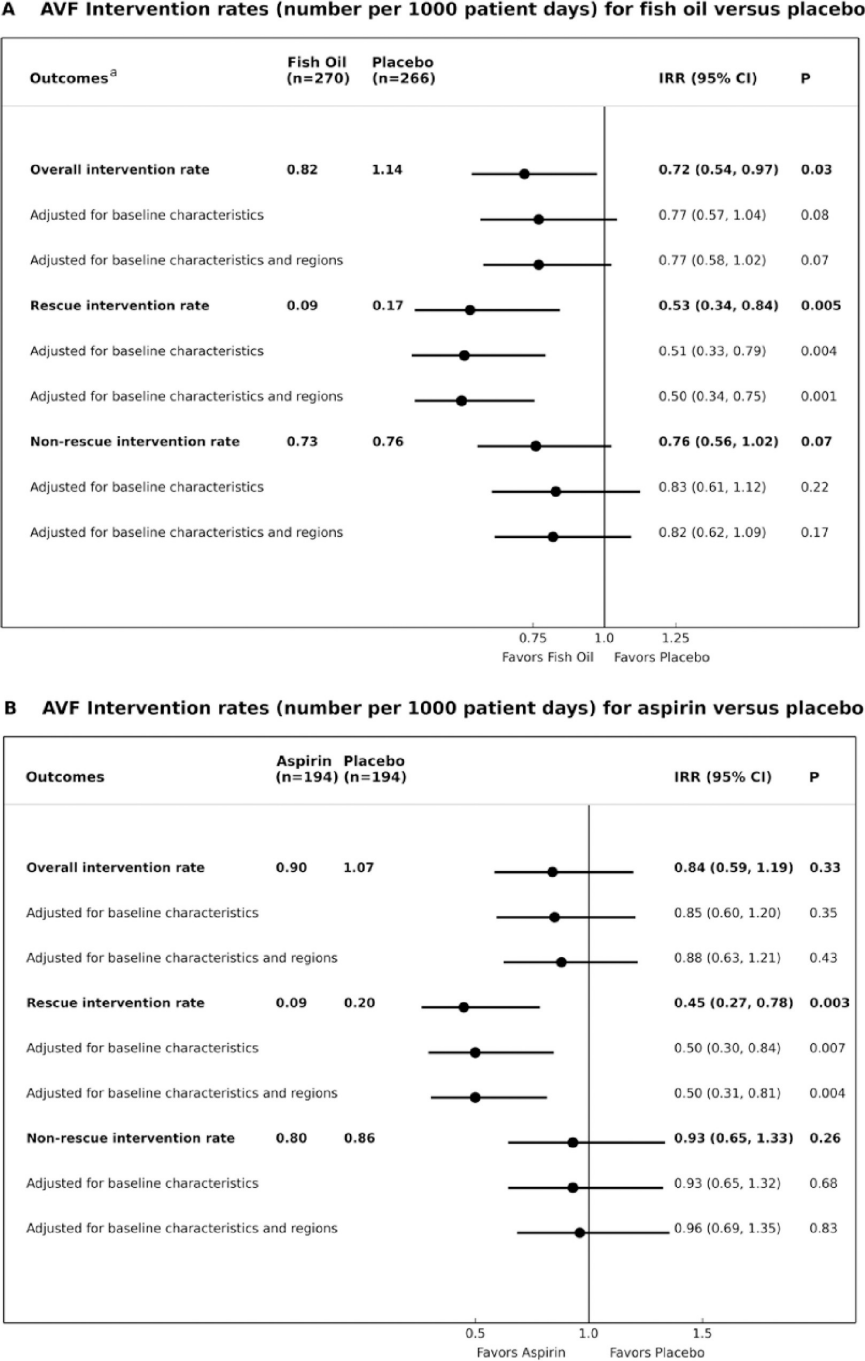
**AVF intervention rates for fish oil versus placebo (A) and aspirin versus placebo (B).** Overall interventions: Surgical or radiological revision or dilatation of the AVF from or proximal to the anastomosis to the ipsilateral central vein, dilation of central venous stenosis, ligation of tributaries, superficialisation of AVF, thrombolysis or thrombectomy, ligation of fistula or salvage by distal reconstruction and interval ligation, others. Rescue interventions: Thrombolysis or thrombectomy. Non-rescue interventions: Surgical or radiological revision or dilatation of the AVF from or proximal to the anastomosis to the ipsilateral central vein, dilation of central venous stenosis, ligation of tributaries, superficialisation of AVF, ligation of fistula or salvage by distal reconstruction and interval ligation. Regions included Australia and New Zealand, Malaysia and the United Kingdom. Pre-specified baseline characteristics included planned AVF site (lower arm, upper arm), diabetes mellitus, age group (quartiles), cardiovascular comorbidity, including any one or more of peripheral vascular disease, ischaemic heart disease and cerebrovascular accidents, and renal replacement therapy at baseline (no [pre-dialysis/transplant], yes [haemodialysis or peritoneal dialysis]). ^a^Adjusted for differences in aspirin use (no aspirin, randomized to aspirin, open-label aspirin).

Fig 2 and S2 Table present the frequency and type of AVF interventions by treatment arms for fish oil versus placebo (A) and aspirin versus placebo (B). Overall, 22% of participants receiving fish oil supplementation required at least one AVF intervention compared to 27% treated with placebo (S2 Table). Of those, 17% treated with fish oil and 28% treated with placebo required more than one intervention. The majority of interventions occurred within the first 6 months of AVF creation (Fig 2). Neither fish oil nor aspirin reduced the risk of needing at least one rescue- or non-rescue intervention compared to their matching placebo. Similarly, the time to first intervention was not significantly reduced by fish oil or aspirin (Fig 3A and 3B; HR 0.85, 95% CI 0.57, 1.26, p = 0.41 for fish oil versus placebo; HR 0.81, 95% CI 0.51, 1.29, p = 0.38 for aspirin versus placebo). As shown in Fig 4A, intervention *rates* were significantly reduced by fish oil compared to placebo, driven by a significant reduction in rescue procedures (IRR 0.53 95% CI 0.34, 0.84, p = 0.005). The effect size remained similar when adjusting for pre-specified baseline characteristics and geographical regions. Of note, there was a significant reduction in the number of rescue interventions with fish oil during the active treatment period, i.e. the first 3 months, compared with placebo (p = 0.009) (Fig 2A). Similarly, the rate of rescue interventions was reduced in the participants treated with low-dose aspirin compared to matching placebo (Fig 4B). The fish oil by aspirin interaction test was not statistically significant for rescue or non-rescue interventions (p = 0.12).

### Primary patency loss and AVF abandonment

**Fig 5:**
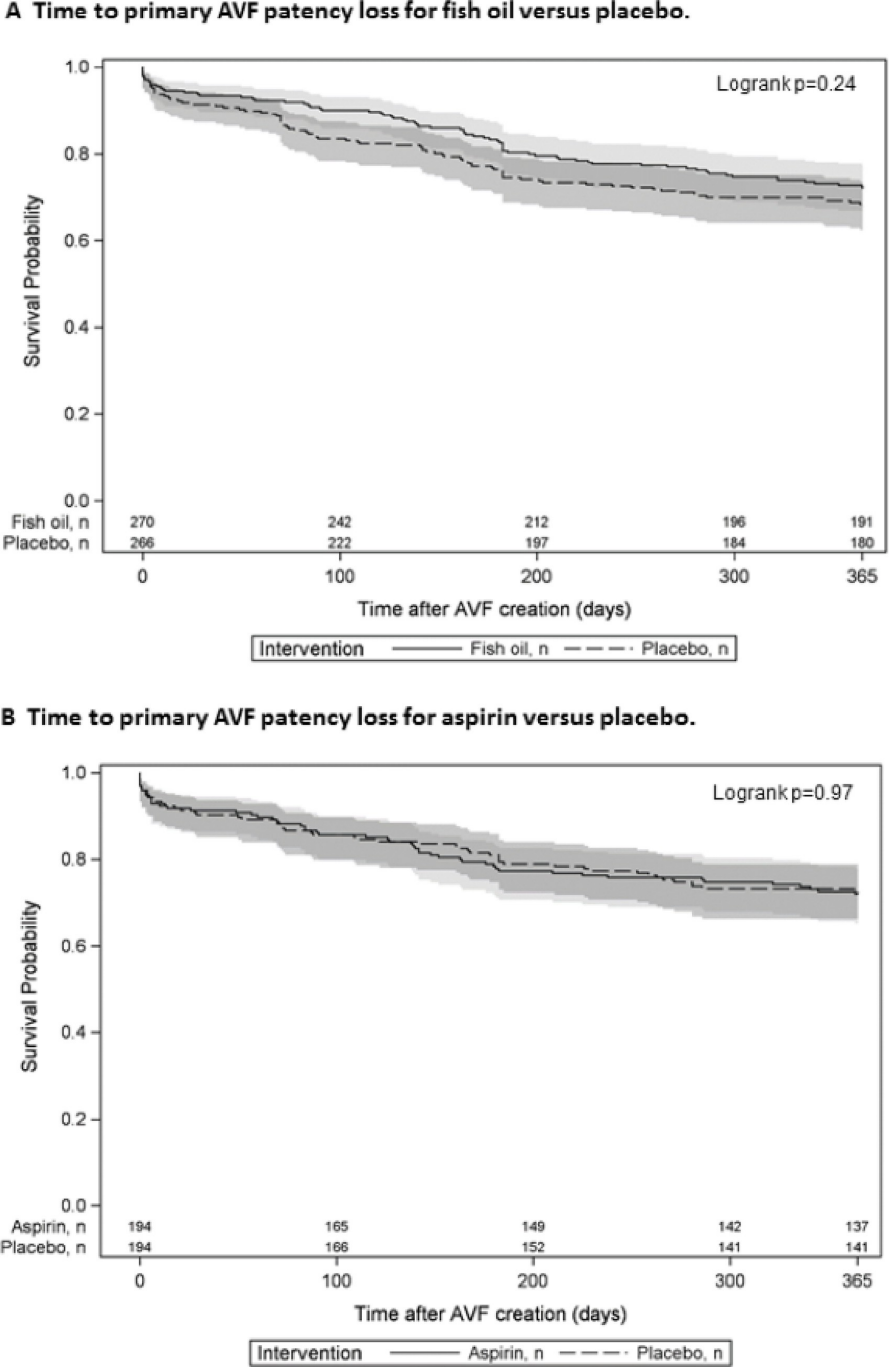
**Time to primary AVF patency loss for fish oil versus placebo (A) and aspirin versus placebo (B).** Kaplan-Meier curve for active therapy (solid line with 95% confidence interval in light gray) and matching placebo (dashed line with 95% confidence interval in dark gray).

**Fig 6:**
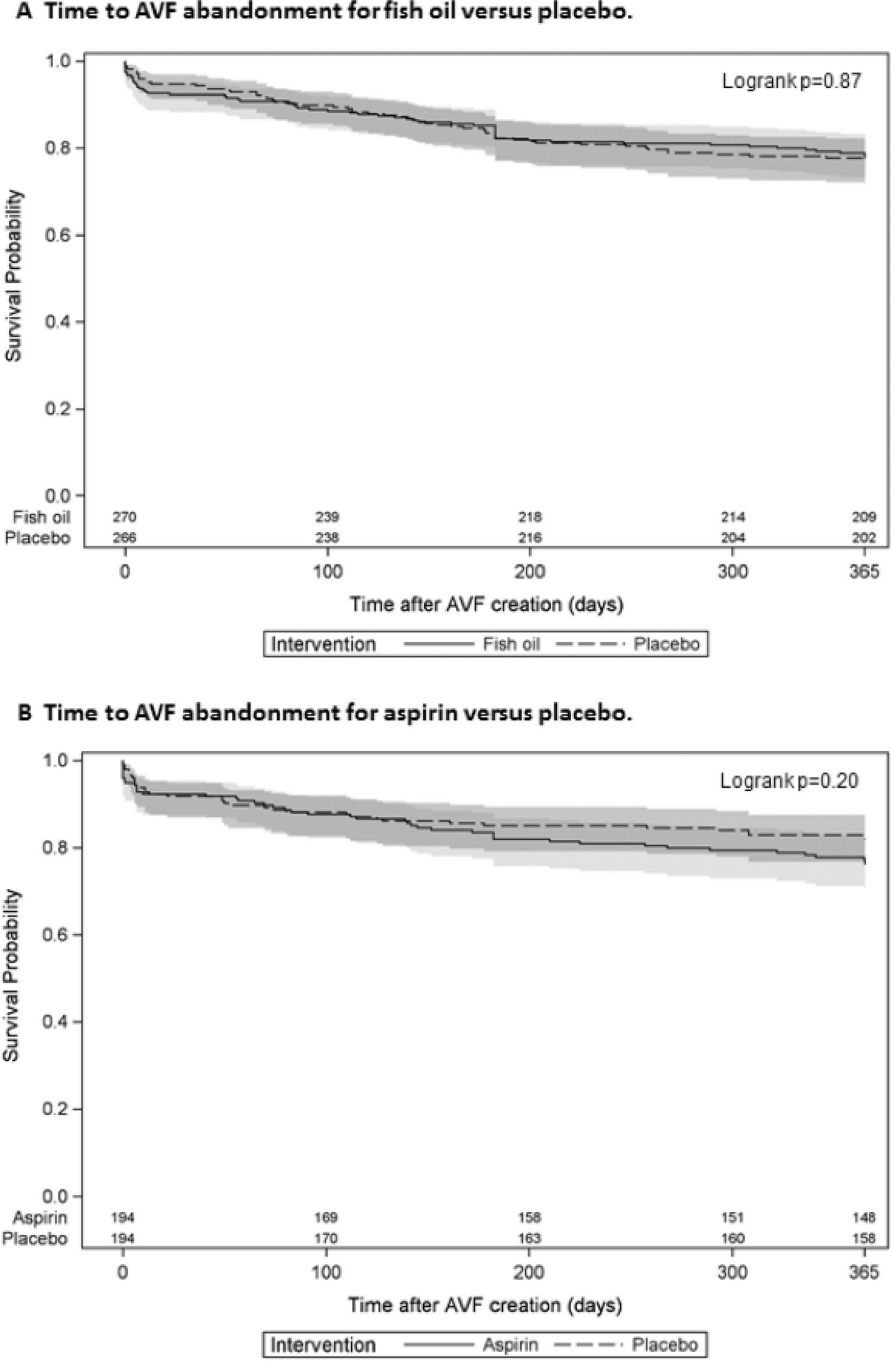
**Time to AVF abandonment for fish oil versus placebo (A) and aspirin versus placebo (B).** Kaplan-Meier curve for active therapy (solid line with 95% confidence interval in light gray) and matching placebo (dashed line with 95% confidence interval in dark gray).

The proportion of participants with primary patency loss within 12 months of their AVF creation was not significantly reduced by fish oil (70/270 [26%] participants) compared to placebo (81/266 [31%]; RR 0.85, 95% CI 0.65, 1.12, p = 0.25). Similarly, the time to primary patency loss was not significantly improved in the fish oil treated group compared to placebo (HR 0.81, 95% CI 0.51, 1.29, p = 0.38), as shown in Fig 5A. In participants treated with aspirin or matching placebo, primary patency loss occurred in 27% of participants in both treatment arms and the time to primary patency loss was similar for aspirin and placebo treated participants (Fig 5B; HR 1.01, 95% CI 0.69, 1.47, p = 0.98). Fig 6 shows that neither fish oil (6A) nor aspirin (6B) led to a significant prolongation in the time to AVF abandonment. Similarly, time to either primary patency loss or AVF abandonment was not altered by fish oil (HR 0.92, 95% CI 0.69–1.21, p = 0.53) or aspirin (HR 1.01, 95% CI 0.73–1.42, p = 0.94).

### CVC requirements

Half of the participants required at least one CVC within the first 12 months of AVF creation and this was not reduced by fish oil (RR 1.00, 95% CI 0.84, 1.19, p = 0.97) or aspirin (RR 0.93, 95% CI 0.76, 1.14, p = 0.48) compared to their matching placebos. The median number of days CVCs stayed in situ was comparable in the fish oil and placebo groups (101 days, IQR 56–175 versus 101 days, IQR 57–176, IRR 0.96, 95% CI 0.76, 1.21, p = 0.73). No significant difference in CVC exposure time was found between participants treated with aspirin or matching placebo (103 days, IQR 63–154 versus 87 days, IQR 54–157, IRR 0.91 95% CI 0.69, 1.19, p = 0.48).

### AVF usability

**Fig 7:**
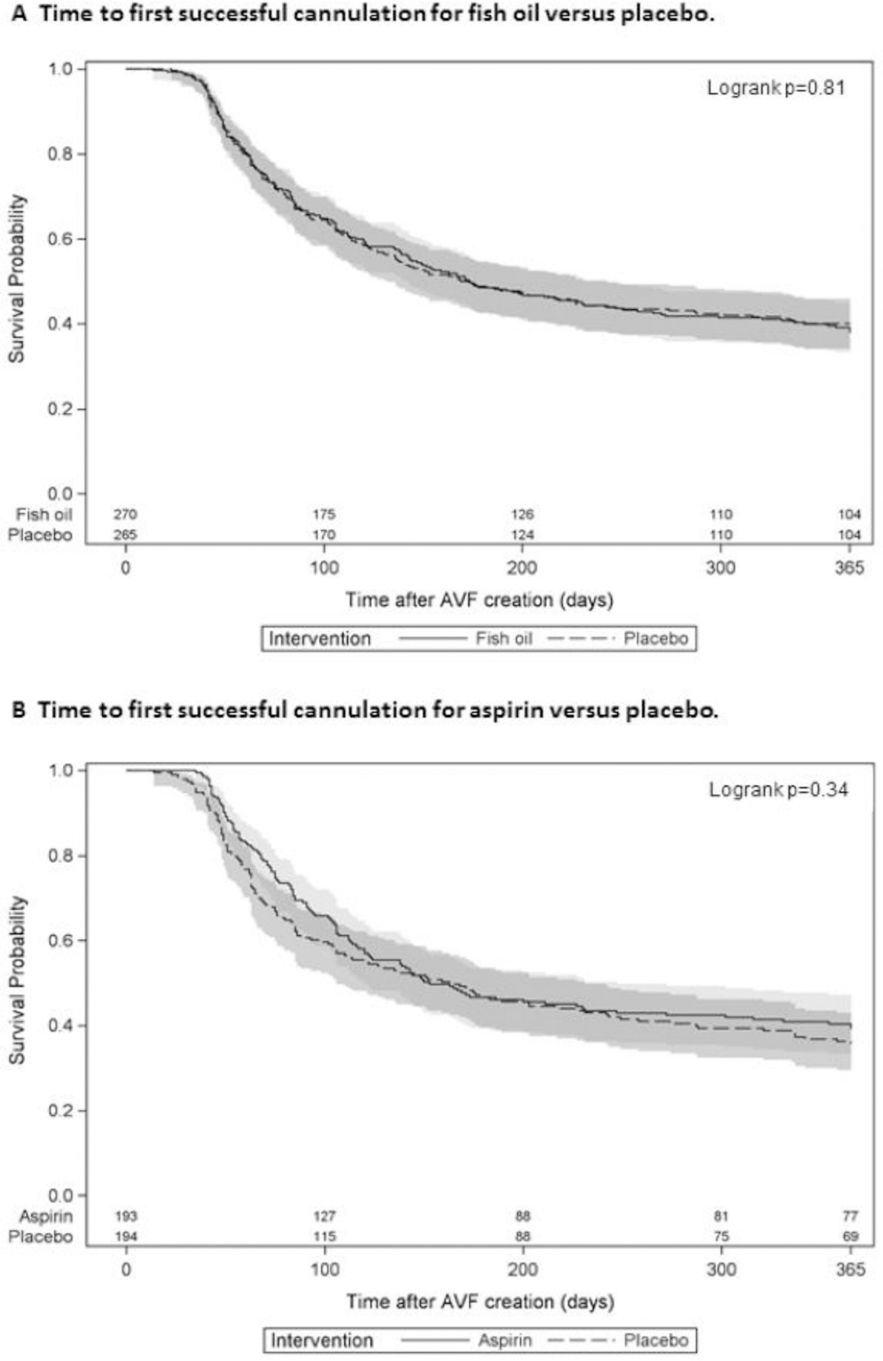
**Time to first successful cannulation for fish oil versus placebo (A) and aspirin versus placebo (B).** Kaplan-Meier curve for active therapy (solid line with 95% confidence interval in light gray) and matching placebo (dashed line with 95% confidence interval in dark gray).

Of the 356 participants who required HD within 6 months of AVF surgery, 36% on fish oil and 34% on placebo were unable to use the AVF for HD (RR 1.05, 95% CI 0.79, 1.39, p = 0.73). Aspirin was similarly ineffective in reducing late dialysis suitability failure compared to placebo (47/135 [35%] versus 38/124 [31%], RR 1.14, 95% CI 0.80, 1.61, p = 0.48). By 12 months following AVF surgery, 444 participants received dialysis and 74% of participants in the fish oil group and 73% of those in the placebo group had three consecutive successful cannulations of their AVF. Neither fish oil (HR 1.03, 95% CI 0.83–1.28, p = 0.77) nor aspirin (HR 0.89, 95% CI 0.69–1.15, p = 0.37) reduced the time to first successful cannulation of the study AVF (Fig 7A and 7B).

## Discussion

Secondary and exploratory outcome analyses of the FAVOURED trial showed that a quarter of participants required at least one AVF intervention within the first year of AVF creation, almost 30% experienced primary patency loss and 50% required at least one CVC. Three months of fish oil supplementation reduced the rate of AVF interventions, principally driven by a 47% relative reduction in rescue procedures for acute thrombosis. Similarly, low-dose aspirin reduced the incidence of rescue interventions by 55%. However, neither fish oil nor aspirin was effective in reducing the frequency and duration of CVC use, the frequency of late dialysis suitability failure or the time to first successful cannulation. Similarly, the time to first AVF intervention, primary patency loss or AVF abandonment did not differ significantly between treatment groups.

Fish oil had no effect on the primary composite outcome of AVF failure[12], defined as the proportion of participants with AVF thrombosis, AVF abandonment and/or cannulation failure within 12 months of access creation. However, these secondary outcome analyses suggested a significant treatment benefit of fish oil in reducing the rate of AVF interventions. Similar findings were reported in arteriovenous grafts by Lok and colleagues[11], whereby fish oil did not significantly reduce the *proportion* of AVG thrombosis or interventions to maintain patency but did lead to a clinically meaningful reduction in *rates* of thrombosis and access interventions. These observations suggest that count outcomes (i.e. rates) are more sensitive to the detection of changes by interventions compared to binary outcomes (i.e. proportions). In addition, a reduction in intervention *rates* but not in either the *proportion* of AVFs requiring intervention or *time to first* intervention suggests that fish oil may be beneficial in reducing *recurrent* interventions. Interventions, such as angioplasties, thrombectomies and revisions, can increase the risk of recurrent thrombosis and stenosis due to disruption of the endothelial layer and subsequent vasoconstrictive, pro-inflammatory and pro-coagulative responses[18]. The vasodilatory[19], anti-inflammatory[8,9], anti-aggregatory[6,7], and anti-proliferative effects[10] of fish oil may be beneficial in reducing this risk and hence the need for recurrent interventions. Additional studies might further explore the potential benefit of fish oil supplementation in secondary prevention of AVF interventions.

High-dose aspirin given as 1000 mg every other day has previously been shown to reduce access thrombosis within the first 28 days of AVF creation[20]. To minimize the risk of bleeding complications, a lower dose of aspirin (100 mg) was used in the FAVOURED trial. Low-dose aspirin, while not associated with increased bleeding, did not reduce the frequency of access thrombosis during the first 12 months of AVF creation as previously shown[12]. However, similar to fish oil there may be a potential role for low-dose aspirin to reduce intervention rates for access thrombosis that warrants confirmation in larger trials.

Vascular access function is the most frequently reported vascular access outcome but is very heterogeneous with almost 900 measures used to assess the usability and function of an access[5]. These additional analyses from the FAVOURED study provide novel information across a broad range of outcomes related to dysfunction of a newly created AVF including patency loss, need for interventions, CVC exposure, cannulation failure and access abandonment. Patients, caregivers and health professionals all consider the need for access interventions the most important clinical outcome measure of the function of a vascular access[21]. From a patient’s perspective, the number of interventions and intervention-free time have a dramatic impact on their quality of life and well-being because access procedures are burdensome and time-consuming[21,22]. Financial costs associated with AVF procedures account for more than half of the expenditure in the first year of AVF creation even without accounting for secondary expenditure for prolonged hospitalisation or procedure-related complications[3]. It is possible that inexpensive interventions including fish oil or low-dose aspirin to reduce AVF-related procedures may be cost-saving and have a positive impact on the patients’ well-being and warrants evaluation in future studies.

More than a third of participants were unable to use their study AVF for dialysis by 6 months after access creation. This is comparable to the national average of AVF use of 36% in prevalent HD patients of the United States (U.S.)[23] and 39% reported in the U.S. Haemodialysis Fistula Maturation study[24]. Of note, in the North American study investigating the effect of clopidogrel on early fistula thrombosis and dialysis suitability failure[25], 61% of participants were unable to use their fistula reliably for HD. The higher prevalence of cardiovascular disease (25%) and participants of black ethnicity (50%), and the use of a more stringent definition that included a minimum machine blood flow rate of 300 mL/min may have contributed to the higher frequency of dialysis suitability failure in the clopidogrel trial. Neither fish oil nor aspirin in our study, or clopidogrel in the North American study[25], were effective in improving dialysis suitability despite reducing rescue intervention rates and early thrombosis, respectively. Dialysis suitability, while a clinically meaningful and relevant outcome, is multifaceted and not only the result of a complex fistula maturation process but also that of a multidisciplinary team effort comprising different surgical techniques, variations in fistula care and cannulation skills. A single treatment agent may therefore not be sufficient to alter such a complex outcome. Multipronged health service intervention studies that include pharmacological, patient- (e.g. vein preservation, AVF care) and clinician-directed (e.g. surgical technique, cannulation skills, access surveillance) interventions to improve AVF outcomes may be required and warrant further exploration.

Our study addresses multiple clinically meaningful and relevant outcomes to assess AVF function, particularly the need for intervention. However, the study has some limitations that should be considered. Considering the 95% CI, the treatment benefits of fish oil in reducing intervention rates might have been as low as 16% or as large as 76% and a reduction in late dialysis suitability failure by up to 21% cannot be excluded. For enhanced precision in treatment estimates, a larger study would be required. It is possible that fish oil supplementation or aspirin use beyond the first three months may have had prolonged benefits in reducing the need for access interventions or CVCs. Three months of therapy were selected because early thrombosis and physiologic maturation (i.e. AVF flow and vein diameter) typically occur within the first few weeks of AVF creation[26,27]. However, there is significant variability in the time to successful clinical maturation. Studies have shown a range in median time from AVF creation to cannulation of 25 days in Japan to 98 days in the U.S. based on the Dialysis Outcomes and Practice Patterns Study[28]. These observations reflect differences in case-mix, care processes, AVF complications (e.g. infiltrations), and AVF procedures[24] and are not expected to be influenced by fish oil or aspirin use.

### Conclusions

Secondary outcomes of the FAVOURED study suggest that three months of fish oil supplementation or low-dose aspirin use may be beneficial in reducing intervention rates for acute thrombosis in newly formed AVF. However, neither fish oil nor aspirin was effective in reducing CVC exposure, decreasing dialysis suitability failure or prolonging the time to primary patency loss, access abandonment, first successful cannulation or first access intervention. Given the importance of access interventions to patients and health professionals and associated costs, we consider further studies to explore benefits of ongoing fish oil supplementation or low-dose aspirin use, particularly for secondary prevention of access interventions to be warranted.

## Supporting information

S1 File Study protocol. (PDF)

S2 File Statistical analysis plan. (PDF)

S3 File CONSORT 2010 checklist for the FAVOURED trial. (DOC)

S1 Table Outcomes including measurement definition, metrics and method of aggregation. (DOC)

S2 Table Type and frequency of AVF interventions for fish oil versus placebo (A) and aspirin versus placebo (B).
(DOCX)

S1 Fig Flow diagram of randomised participants included in the analysis. (TIF)

## References

[1] Lok CE, Sontrop JM, Tomlinson G, Rajan D, Cattral M, et al (2013) Cumulative patency of contemporary fistulas versus grafts (2000–2010). Clin J Am Soc Nephrol 8: 810–818. 10.2215/CJN.00730112 23371955 PMC3641610

[2] Ravani P, Gillespie BW, Quinn RR, MacRae J, Manns B, et al (2013) Temporal risk profile for infectious and noninfectious complications of hemodialysis access. J Am Soc Nephrol 24: 1668–1677. 10.1681/ASN.2012121234 23847278 PMC3785277

[3] Manns B, Tonelli M, Yilmaz S, Lee H, Laupland K, et al (2005) Establishment and maintenance of vascular access in incident hemodialysis patients: a prospective cost analysis. J Am Soc Nephrol 16: 201–209. 10.1681/ASN.2004050355 15563567

[4] Brown EA, Johansson L, Farrington K, Gallagher H, Sensky T, et al (2010) Broadening Options for Long-term Dialysis in the Elderly (BOLDE): differences in quality of life on peritoneal dialysis compared to haemodialysis for older patients. Nephrol Dial Transplant 25: 3755–3763. 10.1093/ndt/gfq212 20400451 PMC2957589

[5] Viecelli AK, O'Lone E, Sautenet B, Craig JC, Tong A, et al (2018) Vascular Access Outcomes Reported in Maintenance Hemodialysis Trials: A Systematic Review. Am J Kidney Dis 71: 382–391. 10.1053/j.ajkd.2017.09.018 29203125

[6] Mori TA, Beilin LJ, Burke V, Morris J, Ritchie J (1997) Interactions between dietary fat, fish, and fish oils and their effects on platelet function in men at risk of cardiovascular disease. Arterioscler Thromb Vasc Biol 17: 279–286. 9081682 10.1161/01.atv.17.2.279

[7] Rylance PB, Gordge MP, Saynor R, Parsons V, Weston MJ (1986) Fish oil modifies lipids and reduces platelet aggregability in haemodialysis patients. Nephron 43: 196–202. 10.1159/000183829 3724927

[8] Calder PC (2013) Omega-3 polyunsaturated fatty acids and inflammatory processes: nutrition or pharmacology? Br J Clin Pharmacol 75: 645–662. 10.1111/j.1365-2125.2012.04374.x 22765297 PMC3575932

[9] Endres S, Ghorbani R, Kelley VE, Georgilis K, Lonnemann G, et al (1989) The effect of dietary supplementation with n-3 polyunsaturated fatty acids on the synthesis of interleukin-1 and tumor necrosis factor by mononuclear cells. N Engl J Med 320: 265–271. 10.1056/NEJM198902023200501 2783477

[10] Fox PL, DiCorleto PE (1988) Fish oils inhibit endothelial cell production of platelet-derived growth factor-like protein. Science 241: 453–456. 3393911 10.1126/science.3393911

[11] Lok CE, Moist L, Hemmelgarn BR, Tonelli M, Vazquez MA, et al (2012) Effect of fish oil supplementation on graft patency and cardiovascular events among patients with new synthetic arteriovenous hemodialysis grafts: a randomized controlled trial. JAMA 307: 1809–1816. 10.1001/jama.2012.3473 22550196 PMC4046844

[12] Irish AB, Viecelli AK, Hawley CM, Hooi LS, Pascoe EM, et al (2017) Effect of Fish Oil Supplementation and Aspirin Use on Arteriovenous Fistula Failure in Patients Requiring Hemodialysis: A Randomized Clinical Trial. JAMA Intern Med 177: 184–193. 10.1001/jamainternmed.2016.8029 28055065

[13] Irish A, Dogra G, Mori T, Beller E, Heritier S, et al (2009) Preventing AVF thrombosis: the rationale and design of the Omega-3 fatty acids (Fish Oils) and Aspirin in Vascular access OUtcomes in REnal Disease (FAVOURED) study. BMC Nephrol 10: 1 10.1186/1471-2369-10-1 19159453 PMC2637871

[14] Viecelli AK, Pascoe E, Polkinghorne KR, Hawley C, Paul-Brent PA, et al (2015) The Omega-3 fatty acids (Fish Oils) and Aspirin in Vascular access OUtcomes in REnal Disease (FAVOURED) study: the updated final trial protocol and rationale of post-initiation trial modifications. BMC Nephrol 16: 89 10.1186/s12882-015-0089-2 26116581 PMC4482267

[15] Viecelli AK, Pascoe EM, Polkinghorne KR, Hawley CM, Paul-Brent PA, et al (2016) Updates on Baseline characteristics of the omega-3 fatty acids (Fish oils) and Aspirin in Vascular access OUtcomes in REnal Disease (FAVOURED) study. Nephrology (Carlton). 10.1111/nep.12573 26205903

[16] Viecelli AK, Pascoe EM, Polkinghorne KR, Hawley CM, Paul-Brent PA, et al (2016) Baseline characteristics of the omega-3 fatty acids (Fish oils) and Aspirin in Vascular access OUtcomes in REnal Disease (FAVOURED) study. Nephrology (Carlton) 21: 217–228. 26205903 10.1111/nep.12573

[17] Lee T, Mokrzycki M, Moist L, Maya I, Vazquez M, et al (2011) Standardized definitions for hemodialysis vascular access. Semin Dial 24: 515–524. 10.1111/j.1525-139X.2011.00969.x 21906166 PMC3999346

[18] Roy-Chaudhury P, Sukhatme VP, Cheung AK (2006) Hemodialysis vascular access dysfunction: a cellular and molecular viewpoint. J Am Soc Nephrol 17: 1112–1127. 10.1681/ASN.2005050615 16565259

[19] Wang Q, Liang X, Wang L, Lu X, Huang J, et al (2012) Effect of omega-3 fatty acids supplementation on endothelial function: a meta-analysis of randomized controlled trials. Atherosclerosis 221: 536–543. 10.1016/j.atherosclerosis.2012.01.006 22317966

[20] Andrassy K, Malluche H, Bornefeld H, Comberg M, Ritz E, et al (1974) Prevention of p.o. clotting of av. cimino fistulae with acetylsalicyl acid. Results of a prospective double blind study. Klin Wochenschr 52: 348–349. 4600820 10.1007/BF01468835

[21] Viecelli AK, Tong T, O’Lone M, Ju A, Hanson C, et al (2018) Report of the Standardized Outcomes in Nephrology-Hemodialysis (SONG-HD) Consensus Workshop on Establishing a Core Outcome Measure for Hemodialysis Vascular Access. Am J Kidney Dis. 71(5):690–700 10.1053/j.ajkd.2017.12.003 29478866

[22] Taylor MJ, Hanson CS, Casey JR, Craig JC, Harris D, et al (2015) "You know your own fistula, it becomes a part of you"-Patient perspectives on vascular access: A semistructured interview study. Hemodial Int. 10.1111/hdi.12340 26201992

[23] Woodside KJ, Bell S, Mukhopadhyay P, Repeck KJ, Robinson IT, et al (2018) Arteriovenous Fistula Maturation in Prevalent Hemodialysis Patients in the United States: A National Study. Am J Kidney Dis. 71(6):793–801 10.1053/j.ajkd.2017.11.020 29429750 PMC6551206

[24] Allon M, Imrey PB, Cheung AK, Radeva M, Alpers CE, et al (2018) Relationships Between Clinical Processes and Arteriovenous Fistula Cannulation and Maturation: A Multicenter Prospective Cohort Study. Am J Kidney Dis. 71(5):677–689. 10.1053/j.ajkd.2017.10.027 29398178 PMC5916528

[25] Dember LM, Beck GJ, Allon M, Delmez JA, Dixon BS, et al (2008) Effect of clopidogrel on early failure of arteriovenous fistulas for hemodialysis: a randomized controlled trial. JAMA 299: 2164–2171. 10.1001/jama.299.18.2164 18477783 PMC4943222

[26] Dixon BS (2006) Why don't fistulas mature? Kidney Int 70: 1413–1422. 10.1038/sj.ki.5001747 16883317

[27] Robbin ML, Greene T, Cheung AK, Allon M, Berceli SA, et al (2016) Arteriovenous Fistula Development in the First 6 Weeks after Creation. Radiology 279: 620–629. 10.1148/radiol.2015150385 26694050 PMC4851120

[28] Rayner HC, Pisoni RL, Gillespie BW, Goodkin DA, Akiba T, et al (2003) Creation, cannulation and survival of arteriovenous fistulae: data from the Dialysis Outcomes and Practice Patterns Study. Kidney Int 63: 323–330. 10.1046/j.1523-1755.2003.00724.x 12472799

