# Supplementary material for: Fish oil and aspirin effects on arteriovenous fistula function: Secondary outcomes of the randomised omega-3 fatty acids (Fish oils) and Aspirin in Vascular access OUtcomes in REnal Disease (FAVOURED) trial
Source: PLoS One. 2019 Mar 26;14(3):e0213274. doi: 10.1371/journal.pone.0213274 (PMC6435148; doi:10.1371/journal.pone.0213274)
Supplement: S1 File — (PDF) [file pone.0213274.s001.pdf]

AKTN Trial number 06.01

A randomised, double-blind, placebo-controlled, factorial-design trial to assess the effect of aspirin and fish oil (omega-3 fatty acids) in the prevention of early thrombosis in arterio-venous fistulae in patients with Stage IV or V chronic kidney disease requiring haemodialysis

FAVOURED (Fish oil and Aspirin in Vascular access OUtcomes in REnal Disease)

Trial Protocol
Version 9, 30 November 2010.

Principal Investigator

Dr Ashley Irish	Renal Unit, Royal Perth Hospital, Perth<br>phone: 61 8 9224 2546<br>Ashley.Irish@health.wa.gov.au

Trial Management Committee

Dr Alan Cass	The George Institute for International Health, Sydney, Australia
Dr Vlado Perkovic	The George Institute for International Health, Sydney, Australia
Dr Sharan Dogra	Sir Charles Gardiner Hospital, Perth, Australia
Elaine Beller	University of Queensland, Brisbane, Australia
Ass Prof Carmel Hawley	Princess Alexandra Hospital, Brisbane, Australia
Professor Peter Kerr	Monash Medical Centre, Melbourne, Australia
Dr Trevor Mori	University of Western Australia, Perth, Australia
Dr Kevan Polkinghorne	Monash Medical Centre, Melbourne, Australia
Dr Amanda Robertson	Royal Melbourne Hospital, Melbourne, Australia
Dr David Voss	Middlemore Hospital, Auckland, New Zealand
Elvie Haluszkiewicz	Royal Perth Hospital, Perth, Australia
Dr David Gracey	Royal Prince Alfred Hospital, Sydney, Australia
Dr Amanda Mather	Royal North Shore Hospital, Sydney, Australia
Dr Stephen McDonald	Royal Adelaide Hospital, Adelaide, Australia
Dr Hooi Lai Seong	Hospital Sultanah Aminah, Johor Bahru, Malaysia
Dr Colin Hutchison	Queen Elizabeth Hospital, Birmingham, United Kingdom
Dr Chris McIntyre	Derby City Hospital, Derby, United Kingdom
Peta-Anne Paul-Brent	University of Queensland, Brisbane, Australia

Trial Statisticians

Charles Thompson	University of Queensland, Brisbane, Australia
Stephane Heritier	The George Institute for International Health, Sydney, Australia

AKTN Project Officer:
Peta-Anne Paul-Brent
phone 61 7 3176 5817
fax 61 7 3176 5663
University of Queensland, School of Medicine
Ground Level, Building 33, Princess Alexandra Hospital
Ipswich Rd, Woolloongabba Queensland 4102

Table of contents

1. Schema & Summary 4
1.1 Schema 4
1.2 Summary 5
2. Objectives 5
2.1 Primary efficacy objective 5
2.2 Secondary efficacy objectives 5
2.3 Exploratory efficacy objective 6
2.4 Tertiary efficacy objectives 6
2.5 Safety objective 6
3. Background 6
3.1 Summary of evidence to date 6
3.1.1 Systematic review 6
3.2 Justification for this trial 8
3.2.1 Justification for the inclusion of patients taking aspirin 8
3.2.2 Justification for the amendment of the study outcomes 9
3.2.3 Experimental intervention 9
3.2.4 Control intervention 10
3.2.5 Population chosen 11
3.2.6 How trial results will inform practice 11
4. Trial Design 11
4.1 Study design 11
4.1.1 Design type 11
4.2 Interventions 11
4.2.1 Intervention – experimental 11
4.2.2 Intervention – control 11
4.3 Blinding 11
4.4 Patient population 12
4.4.1 Selection of patients (sources) 12
4.4.2 Inclusion criteria 12
4.4.3 Exclusion criteria 12
4.5 Outcome measures 13
4.5.1 Primary 13
4.5.2 Secondary 13
4.5.3 Tertiary 14
4.5.3 Safety 14
4.6 Significant Study Events 14
4.6.1 End of Study Events 14
4.6.2 Key Study Events 14
4.6.3 Assessment Periods of Study Outcomes 15
4.6.4 Study AVF Abandonment 15
4.6.5 Rescue Intervention Events 15
4.6.6 Patient withdrawal from study medication 16
5. Procedures 17
5.1 Patient recruitment, consent and randomisation 17
5.2 Treatment plan and modifications 17
5.2.1 Experimental 17
5.2.2 Concurrent treatments 18
5.3 Patient monitoring 19
5.3.1 Visit schedule 19
5.3.2 Clinical assessment of outcome 19
5.4 Adverse events 20
5.4.1 Reporting to Regulatory Authorities 21
5.5 Substudies 21
5.5.1 RBC fatty acid substudy 21
5.5.2 Dietary and physical questionnaire 21

5.5.3 Pre-surgical Doppler studies 22
5.5.4 Platelet and Coagulation Function 22
6. Laboratory Procedures and Investigations 22
6.1 Sample handling and storage 22
6.2 Imaging and other investigations 23
7. Data Management 23
8. Quality Assurance 23
8.1 Training 23
8.2 Measurement and laboratory accreditation 23
8.3 Site monitoring 23
8.4 Auditing 23
9. Statistical Considerations 23
9.1 Sample size 23
9.1.1 Primary Outcome: calculation 23
9.1.2 Primary Outcome: justification of assumptions used 24
9.1.3 Planned recruitment rate 24
9.2 Analysis 25
9.2.1 Definition of Populations for Analyses 25
9.2.2 Primary analysis methods 25
9.2.3 Safety analyses 26
9.2.4 Subgroup analyses 26
9.2.5 Interim analyses and trial stopping rules 26
10. Ethical Considerations 26
10.1 Adherence to regulations and guidelines 26
10.2 Ethics committee approvals 27
10.3 Justification of potential risks and benefits 27
10.4 Protection of patient confidentiality 28
10.5 Insurance 28
11. Trial Management 28
11.1 Organisational structure 28
11.2 Publication Policy 29
12. References 30
13. Appendices 33
13.1 Sample Patient Information Sheet (Patients randomised to omega-3 fatty acids and aspirin) 33
13.2 Sample Patient Information Sheet (Patients randomised to omega-3 fatty acids only) 37
13.4 Classification of Chronic Kidney Disease 42
13.5 Lifestyle questionnaire 43
13.6 Data related to patency rates 48

1. Schema & Summary

1.1 Schema

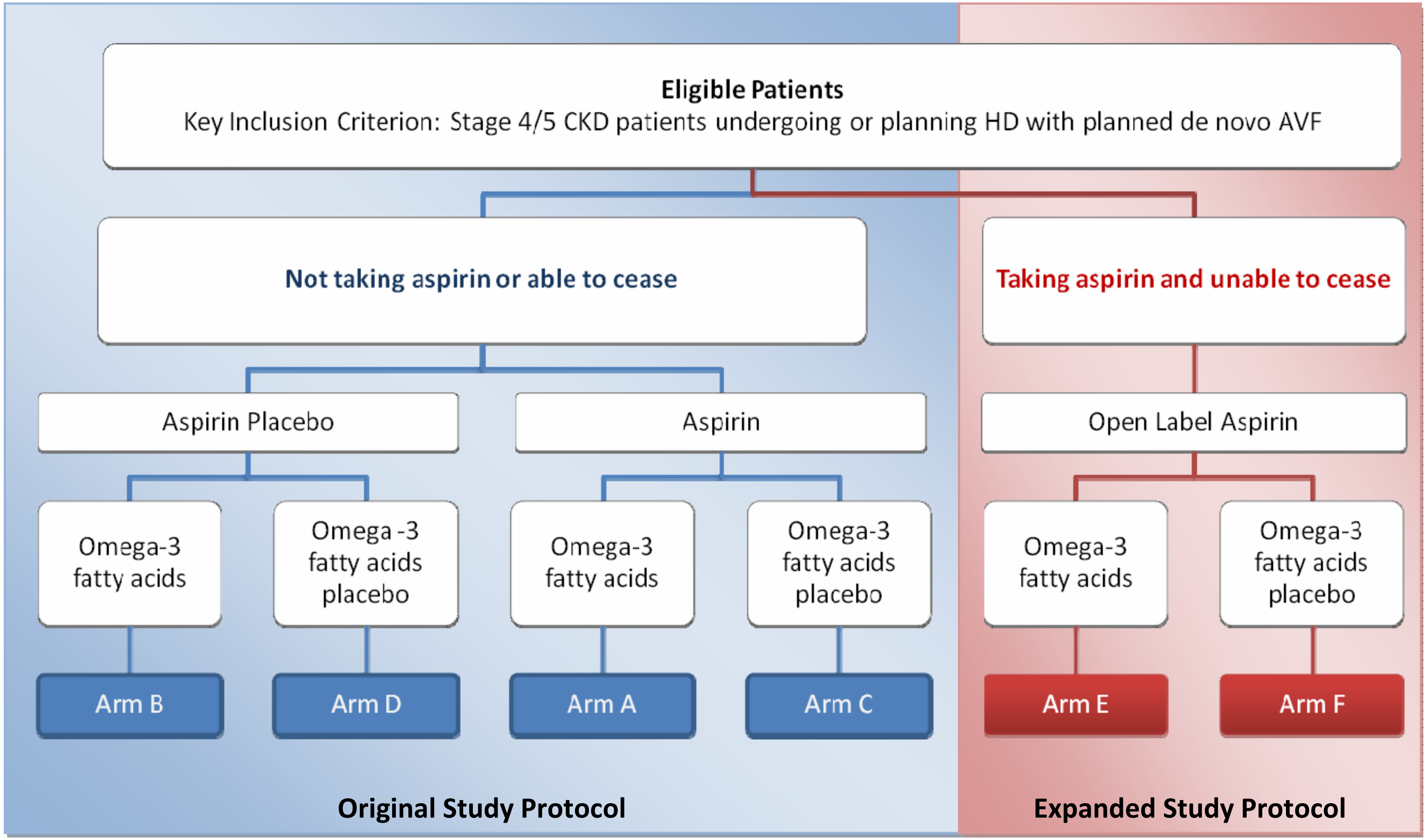

1.2 Summary

FAVOURED is a multicentre, randomised controlled trial with two-by-two factorial design. The objectives of this trial are to determine whether the use of the omega-3 fatty acids and to a lesser extent, aspirin, will effectively improve postsurgical outcomes for patients with de novo arterio-venous fistulae (AVF).

The study population are patients with stage IV or V chronic kidney disease (CKD) who require or will require haemodialysis (HD) and who are scheduled to undergo creation of an AVF. The primary outcome is AVF Access Failure, which is a composite of Thrombosis, AVF Abandonment, and Cannulation Failure during the Cannulation Assessment Period (CAP). Secondary outcomes include AVF access failure according to strata of aspirin use, safety and adverse events of omega-3 fatty acids and aspirin alone or in combination, catheter use, and rescue interventions.

For the purposes of this protocol “omega-3 fatty acids” refers to the two marine-derived long-chain polyunsaturated fatty acids, eicosapentaenoic acid (EPA) and docosahexaenoic acid (DHA).

2. Objectives

2.1 Primary efficacy objective

The primary objective of this trial is to determine whether the use of omega-3 fatty acids reduce the AVF Access Failure rate of de-novo arterio-venous fistulae (AVF), compared to placebo within 12 months post AVF surgery.

2.2 Secondary efficacy objectives

To study the effect of omega-3 fatty acids compared placebo on the following categories of objectives:

1. Thrombosis
    a. the rate of AVF Thrombosis within 12 months post AVF surgery
    b. the rate of primary patency at various time points after surgery (within 24 hours, at weeks 1, 6 and 12 and months 6 and 12)
    c. the rate of rescue interventions within 12 months post AVF surgery
    d. time to the first rescue intervention
2. Permanent AVF abandonment
    a. the rate of permanent abandonment within 12 months post AVF surgery
    b. time to permanent abandonment of the study AVF
3. Cannulation
    a. the rate of Cannulation Failure during the Cannulation Assessment Period
    b. time to the first successful cannulation

4. <u>Central Venous Catheter (CVC) requirement</u>

   a. for haemodialysis on ≥1 occasion between 12 weeks and 12 months post AVF Surgery

   b. for haemodialysis on ≥1 occasion during Cannulation Assessment Period

   c. for haemodialysis on ≥1 occasion from end of Cannulation Assessment Period until 12 months post AVF Surgery

   d. the number of days a CVC is present in situ between 12 weeks and 12 months post AVF Surgery

2.3 Exploratory efficacy objective

To examine whether the use of aspirin reduces AVF Access Failure rate, compared to placebo at 12 months post AVF surgery and possibly on the secondary endpoints listed above.

2.4 Tertiary efficacy objectives

1. To determine whether aspirin and/or omega-3 fatty acids, compared with placebo, reduce the number of days a CVC is present in situ within 12 months post AVF surgery.
2. To study the natural history and long term outcome (beyond 12 months) of the de-novo AV fistulae

2.5 Safety objective

To compare adverse event rates, particularly bleeding, for omega-3 fatty acids and aspirin alone and in combination, compared to placebo.

3. Background

3.1 Summary of evidence to date

3.1.1 Systematic review

Severe chronic kidney disease (CKD) is increasing in both incident and prevalent cases due to ageing of the population and co-morbid conditions such as diabetes. Haemodialysis (HD) is utilised by 70% of patients with end-stage kidney disease (ESKD) in Australia. However, effective haemodialysis is critically dependent upon obtaining and maintaining repeated access to the circulation. Delivering the required blood flow (>300ml/minute) necessary has long been referred to as the “Achilles’ heel” of haemodialysis. Vascular access options include native arterio-venous fistula (AVF), synthetic arterio-venous graft (AVG) or central venous catheter (CVC). AVF is universally acknowledged as the optimal access device with the best long-term patency, lowest cost and lowest infection rates [1-3] and is the most prevalent access used in Australasia (75%) [4]. Because vascular access related surgical procedures and complications of vascular access represent a high proportion of all admissions in the ESKD population and are a major economic burden for health care providers [5], various international initiatives have made this a focus of quality care improvements [6].

Complications associated with artificial vascular access devices (AVG and CVC) include sepsis (especially staphylococcal), vascular malfunction (thrombosis) and death. Several studies confirm the risk (Hazard Ratio) of all cause death is (using AVF as the reference group) around 1.5 for AVG and 3.0 for CVC with similar rates for infectious mortality [2, 3, 7].

While the clinical imperative is to establish AVF in as many patients as possible, primary failure due to thrombosis and failure of mature are major impediments to clinical success [8]. Reports in the literature from the 1970s and 1980s generally describe primary failure rates of between 10 – 20%. More contemporary publications (1990's on) are less encouraging with reports of the primary failure rates increasing to between 20 – 54%. The increase of primary failure in the more recent era may reflect changing patient factors, such as older patients and a higher prevalence of diabetes. A meta-analysis examining the primary failure rate of AVF [9] found a pooled estimated primary failure rate of 15.3%. However, this meta-analysis included early studies starting in the 1960s and omitted a number of key studies published after 2002. These more recent studies have a higher primary failure rate (20-54% in studies published since 1999) [1]. Consistent with these findings of high early failure rate, 50% of new patients commence haemodialysis in Australia and New Zealand with a CVC, due to a failed or inadequate AVF [10].

Primary failure usually occurs as a result of one of two processes [8]:
1) Thrombosis, which usually occurs within weeks of the procedure; and
2) Inadequate size of the artery or maturation of the vein.

Strategies to reduce primary failure rates include pre-operative identification of unsuitable anatomy by the use of ultrasound, improved surgical technique and pharmacological interventions designed to prevent vessel occlusion (thrombosis).

Early thrombosis is defined as thrombosis within the first 30 days post-operatively [11]. There have been a number of small trials evaluating pharmacological agents aimed at reducing the early thrombosis rates. These trials utilised the anti-platelet agents: aspirin, sulphinpyrazone and ticlopidine and varied in size from as few as 5 patients up to 261 patients. In one study using ticlopidine , fistula thrombosis rates for the ticlopidine versus placebo groups were 12% versus 19% ($p=0.10$), respectively. Pooled data for ticlopidine suggests a reduction in thrombosis rate from 25% to 12% ($p<0.001$) [12]. Studies using sulphinpyrazone showed variable results, but were underpowered with the largest study enrolling only 36 patient [13]. Andrassey et al (n=92) compared patients given 500mg aspirin daily for 4 weeks with placebo; the thrombosis rate in the aspirin group was 4% compared with the placebo group of 24%, OR 0.15 (0.03, 0.73) [14]. Another study [15] with a smaller sample size (n=68), did not demonstrate a benefit from aspirin.

Studies examining the effect of omega-3 fatty acids on vascular access are not common. There has only been one study exploring the efficacy of omega-3 fatty acids in the prevention of vascular access thrombosis. This study involved the use of omega-3 fatty acids as a single agent and explored its use in AVG, not AVF. Importantly, the study demonstrated a dramatic reduction in thrombosis at 1 year in patients with AVG [16]. There has also been a study that examined the effect of low-dose aspirin in combination with omega-3 fatty acids on whole blood eicosanoid production [17]. This demonstrated an additive effect of the combination on thromboxane A_2 (TxA_2) (40% with aspirin alone vs. 62% with the combination) and a smaller reduction in the concentrations of prostacyclins (PGI_2 and PGI_3) compared with aspirin alone. There are other effects of omega-3 fatty acids which may be particularly beneficial in patients with CKD, including

improvement in lipid profile [16, 18, 19], blood pressure and heart rate reduction [20], and attenuated inflammatory responses [21] and oxidative stress [22]. Other postulated but as yet unproven benefits in the CKD population may include a reduction cardiovascular mortality and a reduction in uraemic pruritis [23].

3.2 Justification for this trial

The failure of vascular access is the most common cause for hospital admission in CKD patients. Although there is some data to suggest that anti-platelet agents may increase the primary patency of AVF, the limited evidence base and the uncertainty regarding the choice of agent has not supported the widespread use of anti-platelet agents in the prevention of AVF thrombosis. The number of patients requiring dialysis is growing and the maintenance of vascular access in these patients is one of the major challenges faced by health workers in this area, both in terms of clinical input required and economic burden. Furthermore, studies on the natural history of vascular access outcomes show an increase in the incidence of primary failure, which is most likely related to the changing demographics of patients with an increasing prevalence of older patients and diabetic status. Moreover, there has never been an adequately powered study examining the efficacy of anti-platelet agents in the prevention of AVF Access Failure.

3.2.1 Justification for the inclusion of patients taking aspirin

At almost two years into the study, patient recruitment in the FAVOURED trial has been much lower than expected. As of June 2010, 154 patients have been randomised with the recruitment rate about one-ninth of expected. Analysis of screening logs showed that there was a high rate of screening failure. Out of 1,619 patients screened for possible recruitment, 1,180 (73%) were excluded (remaining 323 patients were either suitable but not yet enrolled or their suitability is unknown). The commonest cause of exclusion from the trial was current use of aspirin in 37% patients (433 out of 1,180 patients). Data on the indications for aspirin use is not collected. Our repeated attempts over a 12 month period of educating the participating centres on the lack of evidence about benefits of aspirin for secondary prevention failed to improve the recruitment rate. In a DOPPS report published by Ethier *et al*, aspirin usage in patients on haemodialysis in Australia and New Zealand was 41% [24]. These data strongly support our findings that aspirin usage is a major barrier to recruitment. It is expected that allowing the inclusion of patients on aspirin will significantly boost the recruitment rate.

Analysis of baseline characteristics of the recruited patients showed that the current study population was young and healthy (with a mean age 55 years and an ischaemic heart disease rate of 4%). Since the current study population does not represent contemporary CKD/ESRD population, there is a possibility of weakening the external validity of the study. Previous observational studies have shown that dialysis patients on aspirin were likely to be older with significant cardiovascular disease [24-26]. Thus, with the expanded randomisation process, the study population is more likely to represent the contemporary dialysis population.

A blinded analysis of primary outcome showed much lower primary patency failure rate at 12 weeks than expected (5% vs. 25%). A possible explanation for such a low failure rate could be the relatively young and healthy population as outlined above. Since only 88 patients were included in this analysis, it would be premature to conclude that the study may be under-powered due to very low event rates. Still, if this trend persists, a possibility of an under-powered study cannot be ruled out. The observational studies

have showed patients taking aspirin were more likely to experience a vascular event possibly due to increased age and cardiovascular burden [24, 26]. It is expected that with the amended study design, the event rates (and hence the study power) will increase with the possibility of recruiting older patients with greater cardiovascular burden.

3.2.2 Justification for the amendment of the study outcomes

The FAVOURED trial was designed before the pivotal study examining the effect of Clopidogrel (an anti-platelet agent) in AVF was published [27]. In this study, a total of 877 patients were randomly assigned to clopidogrel or placebo for 6 weeks. Clopidogrel reduced the frequency of early thrombosis of new AV fistula (12.2% vs. 19.5%, relative risk 0.63; 95% confidence interval 0.46 to 0.97). Despite this, failure to attain suitability for dialysis did not differ between the clopidogrel and placebo groups (61.8% vs. 59.5%, respectively; relative risk, 1.05; 95% confidence interval, 0.94 to 1.17); Thus demonstrating that 'thrombosis' is not a valid surrogate for usability or suitability of AV fistula. The FAVOURED Trial Management Committee decided that the primary outcome should be amended to a more clinically relevant outcome - AVF Access Failure, which is a composite of Thrombosis, AVF Abandonment and Cannulation Failure (the inability to cannulate the AVF during a specified period after AVF surgery).

3.2.3 Experimental intervention

Aspirin has been chosen as one of the interventions in this study because of its well-established anti-platelet effects, the trend to efficacy in the underpowered studies to date and because it is both inexpensive and an agent familiar to clinicians. Its use as an anti-platelet agent is well established in clinical practice for other purposes relating to thrombosis prevention particularly in patients with established cardiovascular disease. The anti-platelet effect of aspirin is mediated by its ability to inhibit the platelet cyclooxygenase enzyme resulting in blockade of the synthesis of the pro-aggregatory vasoconstrictor, thromboxane A_2 (TxA_2).

Omega-3 fatty acids have a number of biological effects which make it an attractive agent for the prevention of vascular access thrombosis, including the inhibition of platelet aggregation, anti-inflammatory effects and anti-proliferative actions. Omega-3 fatty acids and aspirin both affect the balance between the pro-aggregatory and vasoconstrictor effects of TxA_2 and the anti-aggregatory and vasodilator effects of prostacyclin (PGI_2) but the mechanism of their effect is different. Omega-3 fatty acids have a weaker inhibitory effect on TxA_2 level, its effect mediated by reducing the availability of arachidonic acid (AA), a precursor of TxA_2. In addition, omega-3 fatty acids lead to an increase in PGI_3 formation (anti-aggregatory and vasodilatation effects equipotent to PGI_2) [17]. The anti-inflammatory effect of omega-3 fatty acids is mediated via a reduction in leukotriene and cytokine production. Theoretically then, a combination of aspirin and omega-3 fatty acids should result in a more favourable effect on platelet aggregation than either agent used alone.

The choice of 100mg aspirin for patients randomised to aspirin is based on this dose having adequate anti-platelet aggregation properties and is the dose most commonly used in patients with cardiovascular disease. The dose of omega-3 fatty acids (4g daily) has been shown to be well tolerated and has benefited multiple cardiovascular risk factors in patients at increased risk of cardiovascular disease [28-32]. The omega-3 fatty acid capsules chosen (Omacor©), are commercially available and provide the highest concentration of omega-3 fatty acids per gram of oil. We have chosen an oral route of administration for both aspirin and omega-3 fatty acids in this study. The oral absorption of both agents is excellent and thus alternative routes of administration are not required.

Bleeding episodes are a concern for clinicians in relation to the use of aspirin in this cohort. Even in the general population on chronic low dose aspirin, the risk of serious gastrointestinal bleeding is doubled [33] and the theoretical risk is higher in patients with CKD because of the presence of impaired haemostasis from multiple mechanisms. There have been published studies [34, 35], showing a significant elevation in bleeding times in patients on haemodialysis treatments administered a single dose of aspirin, but surprisingly little evidence-based clinical trial data in this population. This has been explored in the UK-HARP-I study. In this 2x2 factorial design study involving simvastatin and aspirin as active therapies, allocation to the aspirin group was not associated with an excess of major bleeds (2% in patients on aspirin vs. 3% in patients not on aspirin), but there was a three-fold increase in minor bleeding [36]. Importantly though, in this study, patients received aspirin for 12 months, whereas our study has a much shorter period on therapy of 3 months. Exploring the potential risk of even short-term aspirin is a major secondary outcome of this study.

Omega-3 fatty acids appear to be well tolerated, even in high doses, with gastrointestinal complaints particularly nausea, vomiting, diarrhoea and non-specific discomfort being the most often reported. In studies looking at the effect of omega-3 fatty acids on bleeding time it has not been found to be significantly prolonged [37]. In studies that have been done in a CKD cohort, there have been no clear effects demonstrated in relation to platelet aggregation and bleeding times. There has only been one report of a serious bleeding event in a single patient in an uncontrolled study [38]. Furthermore, it has been reported that administration of omega-3 fatty acids can protect the gastric mucosa against aspirin-induced injury, the postulated mechanism being that omega-3 fatty acids counteract the effect of aspirin on the decrease in prostacyclin (combined effect of PGI_2 and PGI_3) [39, 40]. Thus the combination of aspirin and omega-3 fatty acids may be expected to be additive in terms of efficacy in vascular access thrombosis, but to have a lower risk of bleeding complications than the use of aspirin alone.

The design of a 2x2 factorial study is based on the hypothesis that aspirin and omega-3 fatty acids will both be effective therapies and that the combination of aspirin and omega-3 fatty acids will be additive, but not synergistic. In relation to safety concerns, although both aspirin and omega-3 fatty acids prolong bleeding time, the combination may be expected to be safer than aspirin used alone.

The modified protocol will allow inclusion of patients who are already on aspirin at the time of enrolment and not able to cease treatment. This protocol amendment will result in the majority of patients being randomised to arms E and F (approximately 75% of the remaining recruitment target will be randomised to omega-3 fatty acids or placebo) and fewer patients (approximately 25% of the remaining recruitment target) will be randomised to the arms A, B, C and D (omega-3 fatty acids and aspirin or placebo arms – Please refer to section 1.1 for study schema). The resultant sample size will have adequate statistical power to test the omega-3 fatty acids hypothesis, but not the aspirin hypothesis. Hence, the primary objective is restricted to the omega-3 fatty acids hypothesis and the aspirin hypothesis, is downgraded to a secondary objective. Despite of inadequate statistical power, recruitment of eligible patients to the arms A, B, C and D will be continued to obtain the following crucial data: (1) adverse effects of omega-3 fatty acids alone or in combination with aspirin, (2) adverse effects of aspirin.

3.2.4 Control intervention

Currently patients are not given any prophylactic agents in the majority of cases. This is because the studies to date using anti-platelet agents for secondary prevention have not

been definitive in demonstrating an effect, and concern exists about the safety of the agents in respect to bleeding events. Therefore use of placebo is appropriate.

3.2.5 Population chosen

The study will be multi-centre, and open to all public and private hospital renal units that perform vascular access procedures and will be conducted in Australia and selected overseas countries, namely New Zealand, the United Kingdom, and Malaysia.

3.2.6 How trial results will inform practice

If the trial demonstrates a positive effect of either or both agents, this will lead to a reduction in thromboses, quicker time to dialysis access, and a need for less revisional surgery.

If the trial demonstrates no effect of the agents, this will suggest that platelet aggregation is not the major mechanism for thrombosis in this population and that it may be the poor status of vessels used to create the anastomosis that is a more important factor.

4. Trial Design

4.1 Study design

4.1.1 Design type

The original protocol is a 2x2 factorial-design trial, where patients are randomised to aspirin or matching placebo, and also to omega-3 fatty acids or matching placebo, resulting in four treatment groups. Randomisation will be achieved using a minimization method, balancing over the two stratification factors of 1) study site and 2) upper versus lower arm AVF.

Patients who are currently not taking aspirin or those taking aspirin but are able to cease treatment for the purpose of entering into the trial will be randomised as above. Suitable patients who are on aspirin at the time of screening and are unable to cease, will proceed with the expanded protocol, in which they will be allowed to continue with open-label aspirin and randomised to omega-3 fatty acids or matched placebo only.

4.2 Interventions

4.2.1 Intervention – experimental

The principal action of both aspirin and omega-3 fatty acids is by their ability to reduce platelet aggregation. Omega-3 fatty acids have other potentially beneficial effects on rheology, inflammation and blood flow.

4.2.2 Intervention – control

Matching placebo for each agent.

4.3 Blinding

Each of the two interventions will be studied using a matching placebo, in order to make this a double-blind trial. At the completion of treatment, patients and investigators will be asked which treatment they believe the patient has been receiving, in order to have a measure of the degree of effectiveness of blinding.

In addition, the primary outcome of AVF Access Failure will be made by an independent observer, unaware of the patient's treatment assignment.

4.4 Patient population

4.4.1 Selection of patients (sources)

Patients will be recruited through renal units. The study will be multi-centre, and conducted in Australia and selected overseas countries, namely New Zealand, the United Kingdom, and Malaysia. Public hospital renal unit haemodialysis patients are likely to represent the full population of haemodialysis patients well, as most such patients are treated in these units.

4.4.2 Inclusion criteria

Patients must meet the following inclusion criteria:

1. Stage 4 or 5 Chronic Kidney Disease
2. Currently on haemodialysis or haemodialysis is planned to start within 12 months (including patients currently on peritoneal dialysis).
3. Planned AVF will be the primary haemodialysis access mechanism.
4. Surgery to create an arterio-venous fistula in the upper or lower arm is planned.
5. Aged over 19 years
6. Treating team agreeable to patient's involvement in the trial
7. Informed consent

4.4.3 Exclusion criteria

Patients must not meet any of the following exclusion criteria:

1. Revision of existing AVF rather than de novo AVF
2. Medical indication for anti-platelet or thrombolytic agents*****
3. Known intolerance of agents including hypersensitivity to aspirin, allergy to any other NSAIDs or fish
4. Current use of aspirin within two weeks of commencing trial, or of omega-3 fatty acids within 4 weeks of commencing trial*****
5. Pregnancy, lactation or intention to fall pregnant during the time course of the study
6. Known bleeding disorder or established diagnosis of active or suspected bleeding
7. History of GI ulcers or bleeding within the last 3 months
8. Platelet count less than 100 x 10^9 /L
9. Known active peptic ulcer disease
10. Severe hepatic insufficiency
11. Already receiving anti-coagulation therapy such as warfarin
12. Receiving regular non-steroidal anti-inflammatory (NSAIDS) agents for another indication such as arthritis
13. Syndrome of asthma, rhinitis and nasal polyps if uncontrolled on usual therapy
14. Plan to have other (non-access) surgery within 2 weeks of trial medication period where, in the opinion of the investigator, aspirin or omega-3 fatty acids would be contraindicated for the planned procedure.
15. Potential non-compliance with treatment regimen in the view of the treating clinicians
16. Involved in another clinical trial where the intervention being trialled is likely to confound the outcome of this trial
17. Previously randomised to this trial.

***Note:** Patients who fail to meet the exclusion criteria 2 (Medical indication for anti-platelet or thrombolytic agents) and exclusion criteria 4 (Current use of aspirin within two weeks of commencing trial), and who are otherwise suitable will be enrolled into the expanded protocol.

4.5 Outcome measures

4.5.1 Primary

AVF Access Failure is a composite of

- Thrombosis: This is defined as the absence of a thrill or bruit clinically and/or the requirement of Rescue Intervention to restore patency for thrombosis or occlusion for the study AVF between AVF Surgery and Month 12 Visit. Please refer to section 4.6.5 for further information about Rescue Intervention events.
- AVF Abandonment: This is defined as the permanent abandonment of study AVF between AVF Surgery and Month 12 Visit. Please refer to section 4.6.4 for further information about AVF abandonment.
- Cannulation Failure: This is defined as the failure to successfully cannulate the study AVF with 2 needles (or with 1 needle if using single needle dialysis method) during 8 or more 12 HD sessions. Please refer to Section 4.6.3 for further information about Assessment Periods.

4.5.2 Secondary

Thrombosis

- Thrombosis: This is defined as the absence of a thrill or bruit clinically and/or the requirement of Rescue Intervention to restore patency for thrombosis or occlusion for the study AVF between AVF Surgery and Month 12 Visit. Please refer to section 4.6.5 for further information about Rescue Intervention events.
- Primary patency at various time points: This is defined as the presence of an audible bruit over the site of the arterio-venous anastomosis at time points of within 24 hours post surgery (Recovery visit), and Visits Weeks 1, 6 and 12 and Months 6 and 12.
- Number and type of Interventions: This is defined as the number and type of interventions (both rescue and non-rescue) the study AVF requires between AVF Surgery and Month 12 Visit. Please refer to section 4.6.5 for further information about Rescue Intervention events.
- Time to first Rescue Intervention: This is defined as the time from AVF creation to first occasion of rescue intervention up to Month 12 Visit. Please refer to section 4.6.5 for details of the Rescue Intervention events.

Permanent AVF abandonment

- AVF Abandonment: This is defined as the permanent abandonment of study AVF between AVF Surgery and Month 12 Visit. Please refer to section 4.6.4 for further information about AVF abandonment.
- Time to AVF Abandonment: This is defined as the time from AVF Surgery to permanent abandonment of study AVF up to Month 12 Visit. Please refer to section 4.6.4 for further information about AVF abandonment

Cannulation

- Cannulation Failure: This is defined as the failure to successfully cannulate the study AVF with 2 needles (or with 1 needle if using single needle dialysis method) during 8 or more 12 HD sessions. Please refer Section 4.6.3 for the Cannulation Assessment Periods.
- Time to the first Successful Cannulation: This is defined as the time taken from the AVF Surgery until the first successful attempt at access cannulation up until Month 12 Visit.

Central Venous Catheter (CVC) requirement

- CVC Requirement between Visits Week 12 and Month 12: This is defined as the use of a CVC on any occasion to provide vascular access for HD between Week 12 and Month 12 Visits.
- CVC Requirement during CAP: This is defined as the use of a CVC on any occasion to provide vascular access for HD during Cannulation Assessment Period. Please refer to section 4.6.3 for further information about assessment periods.
- CVC Requirement after CAP: This is defined as the use of a CVC on any occasion to provide vascular access for HD after Cannulation Assessment Period to Month 12 Visit. Please refer to section 4.6.3 for further information about Assessment Periods.
- Days of CVC: This is defined as the number of days a CVC is present in situ between Week 12 and Month 12 Visits.

4.5.3 Tertiary

- CVC Requirement at any occasion: This is defined as the use of a CVC on any occasion to provide vascular access for HD between AVF Surgery and Month 12 visit.
- Long term outcome of AVF: This is defined as the rate of permanent abandonment at Month 24 and 36 Visits and the time to permanent abandonment up to Month 36 Visit.

4.5.3 Safety

- Adverse events: All serious adverse events and adverse drug reactions (see section 5.4) will be collected up to Month 6 Visit. The analysis of this secondary outcome will focus specifically on bleeding events

4.6 Significant Study Events

4.6.1 End of Study Events

End of Study Events are events that mean the end of study with no further follow up with participants. End of study events include

- death;
- withdrawal of patient's consent;
- events that indicate that the patient is no longer eligible for the study i.e. patient has AVG inserted at Surgery instead of an AVF;
- patient lost to follow up;
- the end of study (12 months from the last patient's surgery).

The ANZDATA registry patient information may be accessed to confirm end of study events, particularly for patients lost to follow up.

4.6.2 Key Study Events

Key Study Events are events that do not mean the end of study for patients but are important to record with relation to study outcome measures and the patients' progress in the study. These events include:

- Recovery of renal function (such that dialysis is no longer needed);
- Transfer to peritoneal dialysis (such that haemodialysis is no longer needed);
- Renal transplant;
- Thrombosis of the study AVF;

- Use of CVC as HD access;
- Imaging of the study AVF.

4.6.3 Assessment Periods of Study Outcomes

Most study outcomes are assessed in the period between AVF surgery and Month 12 Visit, which is the minimum follow up period for study participants.

Variations to this period include:

- Cannulation Failure - The Cannulation Assessment Period (CAP) is based on when the patient commences maintenance haemodialysis. Please refer to Table 1 for the definition of Cannulation Assessment Periods.

Table 1 - Cannulation Assessment Periods (CAPs)

Start of Maintenance HD	Beginning of CAP	Duration of CAP
Prior to Week 12 Visit	First HD session after Week 12 Visit	First 12 consecutive HD sessions
Between Week 12 and Month 12 Visits	1st HD session	First 12 consecutive HD sessions
After Month 12 Visit	No CAP	No CAP

- Serious Adverse Events and Adverse Drug Reactions – SAEs and ADRs will be assessed for the 3 months of treatment and for 3 months afterwards (Up until Month 6 Visit).

4.6.4 Study AVF Abandonment

Abandonment of the study AVF is defined as the point that which the AVF is unable to be cannulated and no further attempts will be made to rescue or revise the access.

The date that the AVF is abandoned is determined at the discretion of site PI. However, events that may indicate AVF abandonment include:

- thrombosis of study AVF;
- imaging of study AVF that shows that the AVF is unusable and not amenable to any intervention for its improvement;
- insertion of another dialysis access including new AVF, AVG, venous catheter or peritoneal dialysis access;
- ligation of study AVF. If the AVF is ligated due to a reason other than thrombosis i.e. steal syndrome or heart disease, this abandonment will not be included in the primary outcome.

4.6.5 Rescue Intervention Events

There are two types of intervention events, Rescue interventions and Non-rescue interventions.

Rescue Interventions in the study are those designed to restore patency (absence of flow demonstrated clinically by absence of bruit/thrill or radiologically by absence of flow) of the AVF and include:

- Medical Thombolysis
- Surgical thrombectomy.

Non-rescue Interventions are those designed to improve functionality in an otherwise patent AVF and include:

- Surgical or radiological revision or dilatation of the AVF from or proximal to the anastomosis to the ipsilateral central vein.
- Dilation of central venous stenosis.
- Ligation of tributaries.
- Superficialisation of AVF.
- Ligation of fistula or salvage by DRIL (Distal reconstruction and interval Ligation) due to distal ischaemia (steal).

4.6.6 Patient withdrawal from study medication

Withdrawal of study medication may be made at the discretion of the site PI following a SAE or key study event. However, subjects should still be followed for the duration of the study where possible, even if they have ceased treatment. This will include attendance at scheduled trial visits, and data collection, particularly of outcomes. The investigator may withdraw a subject from study medication at any time if it is felt to be in the best interest of the subject.

Study treatment may be discontinued if one of the following occurs:

- A major bleeding event (e.g. haemorrhagic stroke, gastro-intestinal bleed);
- The access is abandoned due to thrombosis at any time after the one week assessment;
- Anti platelet therapy or anticoagulation is indicated because of other co morbid events i.e. major cardiovascular event, particularly for patients randomised to original protocol;
- A female patient becomes pregnant while on study medication. The event should be reported to the trial coordinating centre immediately as a medically important SAE. The event will be reported within 24 hours and follow up performed through to delivery;
- Patient receives renal transplant.

Study treatment should be continued if:

- Further access is created (including a new AVF or AVG, peritoneal and venous catheter) because of failure of maturation of the study AVF, i.e. access is still patent;
- Renal function is recovered; the study AVF can still be measured patency.

Table 2: Study medication continuation based on key study event or SAE occurrence

Study Outcomes	Continue Medication?
Renal transplant	No
Renal function recovered	Yes*
Thrombosis of study AVF	No<br>if occurs after 1 week
Pregnancy	No
New AVF or AVG	Yes*
Peritoneal dialysis commenced	Yes*
Venous catheter	Yes*
Major bleed	No
Anti-platelet therapy	No

* at the treating physicians discretion

Patients withdrawn from treatment will still be regarded as being “on study”, and follow up data will be collected, unless the patient withdraws consent.

If a patient withdraws consent for the study they shall be withdrawn from the study. Any patient is free to withdraw their consent at any time without the need to justify their decision.

5. Procedures

5.1 Patient recruitment, consent and randomisation

Participants will be recruited from renal units providing haemodialysis and vascular access services in Australia and selected overseas countries, namely New Zealand, the United Kingdom, and Malaysia. Patients will meet the inclusion and exclusion criteria and be scheduled to undergo surgery for creation of a de novo AV fistula.

Patient consent forms will be approved by the Human Research Ethics Committee at each participating site prior to the beginning of the trial. A sample consent form and patient information sheet is provided to participating sites. Participating sites will file a copy of the approved consent form and information sheet for their site with the coordinating office (AKTN). After discussing the trial, ample time will be given to the participant, accompanying person or legal representative to inquire about the trial and decide whether to participate. No person involved with the trial will coerce or unduly influence the decision of a patient to participate in a trial. A copy of the signed consent form and the patient information sheet will be supplied to the participant. Patient consent must be obtained prior to the registration or initiation of trial procedures. Patients will not be randomised until a signed consent form is filed at site.

The Trial Management Committee (TMC) will monitor the medical literature, and any other relevant information impacting on the continuation of the trial. Consent forms and patient information sheets will be revised should any relevant and important new information become available.

Patients are to be randomised as close as possible to the time of the scheduled procedure and not more than 7 days before the planned procedure. Patients are to commence taking study medications on the day prior to the scheduled surgery. If the procedure is rescheduled, the patient is to cease taking medications if already started and to recommence the day before the rescheduled surgery. Refer to the operations manual for further information. Stratification will occur for study site and upper vs. lower arm site. Patients will be randomised to one of four treatment groups in equal proportion.

5.2 Treatment plan and modifications

5.2.1 Experimental

For all patients; Omega-3 fatty acids 4 gm daily in the form of 4 Omacor capsules (46% eicosapentaenoic acid (EPA) and 38% docosapentaenoic acid (DHA), as the ethyl esters), or 4 matching placebo capsules (olive oil), supplied by Abbott Products, commencing on the day prior to surgery and continuing for 12 weeks.

For patients not taking open-label aspirin; Aspirin 100 mg per day p.o. or matching placebo, supplied by Bayer Healthcare, commencing on the day prior to scheduled surgery and continuing for 12 weeks. Patients taking aspirin and unable to cease will continue with their current open-label aspirin. Aspirin use and dosage should preferably

remain unchanged for these patients during the treatment period (12 weeks), and any deviations or changes recorded on the case report forms (CRF).

To improve compliance and minimise gastro-intestinal discomfort, the omega-3 fatty acids or matching placebo medications are to be taken morning and evening with 2 capsules taken with a cold drink just before breakfast and 2 capsules again taken with a cold drink just before dinner.

If patient's access surgery is postponed, patient should stop medication until 24 hrs before rescheduled surgery. If the surgery is postponed for more than 4 weeks, the patient must have another blood test and their eligibility (conformity with the inclusion/exclusion criteria) must be reassessed. If they remain eligible, they should commence the medication which they have already been allocated.

Compliance will be monitored by capsule/tablet count at scheduled study visits at 12 weeks. Australian and New Zealand centres will also collect erythrocyte fatty acid samples for testing for compliance with omega-3 fatty acid intake. All centres will apply routine biochemistry (lipids, CRP) and haematology analyses as part of patient monitoring (refer to section 6.1)

5.2.2 Concurrent treatments

No other fish oil/omega-3 fatty acid supplement, or anti-coagulation agents are permitted during the period the patient is taking study medication. Patient randomised into the original protocol will not be permitted to take any other anti-platelet agents and patients randomised to the expanded protocol with only be allowed to take their current open-label aspirin which should be maintained at a constant dosage level throughout the treatment period. Heparin or heparin-like agents that are used during vascular surgery or during haemodialysis to flush lines are permitted during the study treatment period. Once the treatment period is completed, patient may commence or recommence omega-3 fatty acids or aspirin product.

In addition, interaction of the following agents with aspirin should be considered:

- Thrombolytic agents
- Methotrexate, particularly at doses of 15mg/week or greater
- Uricosuric agents
- Digoxin
- Sufonylureas or insulin
- Systemic glucocorticoids, except replacement therapy for Addison's disease
- ACE inhibitors, beta blockers
- Valproic acid, phenytoin
- Acetazolamide
- Diuretics (if daily asa dose is >3g)

5.3 Patient monitoring
5.3.1 Visit schedule

Table 3: FAVOURED Study Visit Schedule

Study Phase	Baseline	Randomisation	Surgery	Treatment Period (wks)			Follow-up Period (mths)			
**Assessment**	**≤ 4wks prior to surgery**	**≤ 7 Days prior to surgery**		**1**	**6**	**12**	**6**	**12**	**24**	**36**
Informed Consent	X									
Subject number		X								
Medical History	X									
Weight and Blood Pressure	X		X	X	X	X	X	X	X	X
Demographics	X									
Physical Exam	X									
Dialysis status	X			X	X	X	X	X	X	X
Description and status of AVF			X	X	X	X	X	X	X	X
AVF Access Failure components			X	X	X	X	X	X		
Concomitant meds	X		X	X	X	X	X			
Adverse events			X	X	X	X	X			
**Fasting Pathology Tests**										
Haematology and coagulation	X					X				
Biochemistry and lipid profile	X					X				
Urinary protein:creatinine ratio	X					X				
Optional: Homocysteine and Lp(a)	X					X				
**Stored Samples for ANZ sites** (send to central laboratory)										
RBC fatty acid sample, hsCRP	X				X	X				
Urinary thromboxane B2 and prostacyclin metabolites	X					X				

Notes:

- Baseline visit is to be completed within 4 weeks prior to surgery. It is recommended that all baseline procedures occur at the same visit but consent and physical exam can occur on a separate day. Patients are to be randomised as close as possible to the time of the scheduled procedure and not more than 7 days before the planned procedure. Patients are to commence taking study medications on the day prior to the scheduled surgery. If the procedure is rescheduled, the patient is to cease taking medications if already started and to recommence the day before the rescheduled surgery.

5.3.2 Clinical assessment of outcome

Two separate observers will independently record the presence or absence of bruit at site of anastomosis. A standard stethoscope will be used (not a Doppler stethoscope). The bruit must be present over the site of anastomosis. In the event that the two observers disagree on the presence of the bruit, the bruit would be recorded as absent.

Bruit will be assessed by medical or nursing staff, specifically

- Consultant nephrologists

- Advanced trainees
- Access surgeons
- Dialysis nurses
- Vascular access nurses

In addition, bruit assessment at discharge post surgery may be performed by recovery nurses.

5.4 Adverse events

Adverse events, including any cases of bleeding, will be managed as per usual local clinical care practice.

Adverse Events and Serious Adverse Events will be reported during study medication period and 3 months afterwards only (up until the Month 6 Visit). Only AEs that the site investigator rates as possibly or probably related with the study medication will be recorded. These will primarily include bleeding events and gastrointestinal symptoms. All SAEs will be recorded, whether related to the study medication or not. AEs and SAEs will be recorded in the regular data collection activities of the trial.

For the purposes of this trial the following definitions will apply;

Adverse Event:
An adverse event is defined as "*any untoward medical occurrence in a patient or clinical investigation subject administered a pharmaceutical product which does not necessarily have a causal relationship with this treatment*"

Serious Adverse Event:
A serious adverse event (experience) or reaction is any untoward medical occurrence that at any dose:
- results in death
- is life threatening (refers to an event in which the patient was at risk of death at the time of the event)
- requires inpatient hospitalisation or prolongation of existing hospitalisation
- results in persistent or significant disability/ incapacity
- is a congenital anomaly/ birth defect or
- is a medically important event or reaction.

Medical and scientific judgment should be exercised in deciding whether other situations should be considered serious such as important medical events that might not be immediately life-threatening or result in death or hospitalisation but might jeopardise the patient or might require intervention to prevent one of the other outcomes list in the definitions above.

Suspected Unexpected Serious Adverse Reaction (SUSAR)
Any adverse reaction that is both serious and suspected to be caused by the study medication that is not consistent with the information in the applicable product information (unexpected) is subject to expedited reporting to the relevant Regulatory Body.

Adverse events will be recorded in the regular data collection activities of the trial.

If the AE is assessed as serious, the site PI must report the event to the Coordinating Office immediately or within 24 hours of being made aware of the event. The initial report should be made using the SAE Fax Notification sheet and an accompanying report that includes at a minimum: the name & contact details of the reporter, the event &

seriousness criteria met, the patient's ID number & treatment pack number and date the reporter became aware of the event.

If required, a more detailed follow-up report is to be submitted, within 7 days of the initial report submission, to the Coordinating Office and to the approving Human Research Ethics Committee (either local HREC or Lead HREC for state/country). Please refer to local site HREC guidelines as they may have a specific form they require for SAE reports.

The follow up written reports include: the patient's identification number, a description, seriousness and likely cause of the SAE, concomitant medications, duration of the SAE with start and end dates, and details of reporter.

A 24 hour contact number will operate to allow site staff to contact an appropriate representative of the Trial Management Committee if they believe breaking the blind is warranted. The representative will discuss and if necessary negotiate the necessity of breaking the blind. If this action is deemed necessary, the time, date and reason will be recorded.

5.4.1 Reporting to Regulatory Authorities

The responsibility for reporting serious, unexpected and associated adverse drug events to the relevant Regulatory Authority such as the Therapeutic Goods Administration in Australia lies with the study coordinating office (AKTN). The study coordinating office is also responsible for reporting all serious adverse events to Abbott Products and Bayer Drug Safety as per their contractual agreements.

5.5 Substudies

5.5.1 RBC fatty acid substudy

Omega-3 fatty acids incorporated into the red blood cell membrane will be used as a compliance measure. In addition, there will be a cross sectional assessment looking at RBC omega-3s and patient characteristics.

RBC fatty acids will be measured for all patients at participating sites before the commencement of study medications (at baseline), once the patient has been taking the study medications for 6 weeks and as close as possible to the last dose of study medications. 2-3 ml volume samples will be collected into EDTA, labelled with study number, patient initials and date of collection and stored frozen at -20 degrees or colder. Samples will be sent to the central testing laboratory in batches as advised. Complete details of collection, storage and shipping requirements are provided in the study operations manual.

This substudy will be undertaken by only the Australian and New Zealand sites.

5.5.2 Dietary and physical questionnaire

Many CKD patients have poor diets and malnutrition. This sub-study explores the status and the significance of dietary intake (including fish) and lifestyle factors in relation to patient outcomes. In particular, the questionnaire results will enable the interaction between these lifestyle and dietary factors and response to omega-3 fatty acids supplementation to be explored.

Trial participants will be asked to complete the dietary and physical questionnaire (appendix 13.4) once only, at baseline. Questionnaires will ideally be completed whilst the patient is in clinic and reviewed by the research nurse/clinical trial coordinator while

the patient is still on site and any missing information completed at that time. Original responses should be kept in the patient's trial folder.

This substudy will be undertaken by all participating sites.

5.5.3 Pre-surgical Doppler studies

This sub-study explores whether pre-operative vascular characteristics determined by duplex ultrasound will predict vascular access outcomes either independently or in particular, the response to aspirin, omega-3 fatty acids and the combination.

Interested centres with vascular laboratories will perform standardised evaluation and reporting of arm vasculature by duplex ultrasound prior to surgery. The outline of the standardised methods for evaluation will be provided in the study operations manual.

This substudy will be undertaken by interested sites in any of participating countries with the requisite vein mapping faculties.

5.5.4 Platelet and Coagulation Function

This substudy aims to study the effects of aspirin and omega-3 fatty acids alone and the combination on platelet function, coagulation and platelet activation as well as quantification of compliance with aspirin (prostaglandin metabolites).

At baseline and week 12 spot urine samples will be collected from participants with urinary output for the measurement of protein:creatinine ratio (to be tested by the local laboratory) and urinary thromboxane B2 and prostacyclin metabolites (a 5mL sample stored at a temperature of -20˚C or lower, to be sent with the RBC fatty acid blood samples for testing by the central laboratory).

This substudy will be undertaken by only the Australian and New Zealand sites.

6. Laboratory Procedures and Investigations

6.1 Sample handling and storage

Apart from samples for the above substudies, all other routine blood and urine samples will be analysed by local laboratories, and samples will be collected in accordance with the requirements of these laboratories. The required sampling points for the routine blood tests are shown in table 5.3.1. For the blood samples taken at baseline and Week 12, patients must fast as per individual sites requirements for fasted lipid and glucose tests. Samples for the RBC fatty acid and platelet function substudies will be collected and stored as per section 5.6.1. Baseline blood samples must be tested within 4 weeks of AVF surgery. The routine blood tests required are:

1) Haematology and Coagulation: Full blood count (including Hb, WCC and platelets) and coagulation (INR, APTT and fibrinogen).
2) Biochemistry: Creatinine, urea, electrolytes, calcium, phosphate, albumin, glucose, parathormone and HbA1c. The protein:creatinine ratio of patients with urine output will be tested.
3) Fasting Lipids: Total, LDL, HDL and triglycerides.
4) Optional tests (where these are routinely available) are fasting Homocysteine and Lipoprotein(a).

.

6.2 Imaging and other investigations

Imaging and other investigations will be performed as per normal local practice.

7. Data Management

The timing of forms to be completed follows the schedule in Section 5.3.1 and is outlined in more detail in the operations manual.

Original consent forms are to be stored locally.

All source documents used in this trial, including medical records, should be stored for a period of 15 years following the close of the study.

8. Quality Assurance

8.1 Training

Assessment of bruit is to be performed according to guidelines provided.

8.2 Measurement and laboratory accreditation

A copy of the NATA or other accreditation certificate and local normal ranges will be sent to the coordinating centre prior to the use of any local laboratories.

8.3 Site monitoring

The coordinating centre reserves the right to visit sites for study monitoring purposes. At these visits, the authorised study monitor will require access to the medical records of study patients for the purpose of source data verification. The study monitor will also have access to all other study documentation, including ethics correspondence for the purpose of verifying appropriate study management.

8.4 Auditing

The coordinating centre reserves the right to audit sites. At these visits, the authorised study auditor will require access to the medical records of study patients.

9. Statistical Considerations

9.1 Sample size

9.1.1 Primary Outcome: calculation

The event rate for the primary outcome (AVF Access Failure) is estimated to be 30% at twelve months in the control group. To achieve a 30% relative risk reduction is achievable with omega-3 fatty acids, then with 80% power and a significance level of 5%, 954 study subjects will be required. This allows for 5% drop-in from placebo to active treatment, 5% drop-out from study treatment (to no treatment – assumed equivalent to placebo), and 5% loss to follow-up. This is equivalent to an observed relative risk reduction of 24%. Without adjustment for compliance, the study size needed would have been 734 subjects.

The original 2x2 factorial design of the study would have resulted in equal number of patients distributed in both the parts of the study i.e. (1) aspirin hypothesis, and (2) omega-3 fatty acids hypothesis. The protocol amendment will allow inclusion of patients who are already taking and unable to cease aspirin into testing of the omega-3 fatty acids hypothesis of the study. This will lead to disproportionate allocation of patients to two parts of the study.

We anticipate that at the end of study, 350 to 400 patients will be randomised to either aspirin or its placebo compared with 954 subjects allocated to omega-3 fatty acids or placebo. Thus, the resultant sample size will have adequate power to address the omega-3 fatty acids hypothesis but will not have adequate power to answer the aspirin hypothesis.

However, the assumption of event rate (AVF Access Failure) of 30% is very conservative. The actual event rate is expected to be between 30% and 50%. With increasing event rate, required sample size will reduce as shown in table 4.

Table 4: The enrolment target with increasing event rates

Event rate	Sample size	
	Not adjusted	adjusted
30%	734	954
40%	488	634
50%	340	442

The study is not adequately powered to detect a clinically important difference between the combination of aspirin and omega-3 fatty acids and either treatment alone, but preliminary data on the combination especially on adverse events will be obtained.

9.1.2 Primary Outcome: justification of assumptions used

The event rate of the primary outcome, AVF Access Failure, is based on review of all papers published in this area. The effect size, 30% reduction, is based on the smallest size of the effect the proposing clinicians believe would lead to a change in practice (incorporation of intervention) should the trial prove the intervention is efficacious. AVF Access Failure is preferred as a primary outcome measure over failure of patency because it is more clinically relevant.

9.1.3 Planned recruitment rate

During the initial planning for the study, a review of the data from the ANZDATA renal registry [41] showed that 7,202 patients were receiving dialysis in Australia and New Zealand in December 2004 with 2042 patients commencing dialysis in Australia and New Zealand in that year. Data obtained from the New Zealand renal population and Princess Alexandra Hospital, Queensland, revealed that approximately 1 procedure was performed for every 2.3 - 2.8 patients receiving haemodialysis (total numbers of patients - new patients and prevalent patients). The New Zealand data was obtained by Dr. Mark Marshall (survey between October 2000 and September 2001) and by Dr. Carmel Hawley (Queensland) based on prospectively collected data in PAH renal unit from January 2004 until December 2004. In addition information from the 2005 registry report supports this data.

It was expected that all incident patients require new vascular access, in addition to existing patients on dialysis who needed further access because of failure of previous

vascular access procedures. Twenty seven percent of haemodialysis patients in Australia and 33% of patients in New Zealand, respectively, were incident patients in 2004. With extrapolation to the whole Australian and New Zealand renal population, and estimating 1 procedure per 3 patients allowed us to derive that a total of 2,400 access procedures are performed each year (AVF and AVG). With approximately 75% of access procedures AVF, it was estimated that there was 1,800 de-novo AVF creations per year. Assuming we were able to recruit 22% of these, recruitment for the study was estimated to take approximately three years.

After almost two years of recruitment for the study, patient enrolment has been much lower than expected, with 154 patients enrolled which is 13% of the total recruitment target. The first avenue to increase recruitment was the inclusion of additional sites outside Australia and New Zealand. Sites from Malaysia and the United Kingdom have been included as they have a good track record for recruitment into other renal studies, had a similar renal cohort and treatment regime and would add significant numbers of patients to the study.

Additionally, an analysis of screening logs showed that there was a high rate of screening failure with 73% of patients screened in Australia and New Zealand being excluded. The commonest cause of exclusion was current use of aspirin in 37% of patients excluded. Estimates from Malaysian and United Kingdom sites indicate that aspirin usage is also high in their renal populations (30 – 40%). With a aim of removing this barrier to recruitment, two additional arms have been added to the study which will allow patients who are taking aspirin and are unable to cease (open label aspirin) to be randomised to omega-3 fatty acids or placebo only. It is estimated that this change will improve the recruitment rate by at least double. This improvement combined with the addition of the new overseas sites should mean that the recruitment period will only be extended by 18 months from the original recruitment period of 3 years.

9.2 Analysis

9.2.1 Definition of Populations for Analyses

Analysis of the efficacy outcomes will be on an intent-to-treat basis. All randomised patients will be analysed in the group they were allocated to, even if they do not receive treatment as allocated, or do not commence treatment. All randomised patients will be included in the analysis.

A marginal analysis will be conducted for efficacy outcomes. Tests of hypotheses will be at the 5% significance level, and 2-sided p-values will be used. A statistical analysis plan (SAP) will be written by a blinded statistician and reviewed by the principal investigators (also blinded). It will be this document that the statistical analysis methods outlined in the next paragraphs will be fully developed.

9.2.2 Primary analysis methods

The efficacy analysis will involve several marginal comparisons:

a) Omega-3 fatty acids vs. placebo (with and without aspirin). This will be a comparison of Arms (A+B+E) vs. (C+D+F). Please refer to section 1.1 for further details

b) Aspirin vs. placebo excluding patients who are unable to cease aspirin. This will be a comparison of Arms (A+C) vs. (B+D).

c) Omega-3 fatty acids vs. placebo omega-3 fatty acids in open-label aspirin patients (Arms E vs. F).

The primary outcome is measured as the proportion with AVF Access Failure. The primary hypothesis will be tested using a chi-square test of proportions in a marginal analysis. (comparison A above). A similar strategy will be performed for all binary secondary efficacy objectives. For survival endpoints the log-rank test will be used in the same marginal analysis.

The exploratory efficacy objective is more investigative and involves a marginal comparison of type B for the primary and all secondary efficacy endpoints. A same strategy as before will be used.

An adjusted analysis based on logistic regression or the Cox model will be performed, including the stratification variables in the model for respectively binary and survival endpoints.

9.2.3 Safety analyses

Serious Adverse Events will be tabulated by treatment arm (n, %) and various marginal totals of patients experiencing a particular category of SAE will be presented. The same marginal analyses (type A, B or C) will be considered. Where applicable, Chi-square or Fisher tests will be used to test potential differences in the different marginal comparisons.

9.2.4 Subgroup analyses

Subgroup analysis (omega-3 fatty acids vs. placebo omega-3 fatty acids) stratified by aspirin use (patients unable to cease aspirin versus the others) will also be conducted using the same techniques as above.

9.2.5 Interim analyses and trial stopping rules

Safety reports will be produced regularly and sent to the Data and Safety Monitoring Board (DSMB). Two formal interim analyses will be conducted after 1/3 and 2/3 of total patients have been accrued and followed for sufficient time. The Haybittle-Peto rule will be used as a stopping guideline for efficacy as it offers the flexibility to update the number of looks if need be. If two interim analyses equally-spaced are indeed performed, the level of the final analysis will be 0.048. Similar analyses to the primary one will be undertaken on the secondary outcomes, as well as exploratory analyses looking at the validity of the chosen primary outcomes and its correlation with other outcomes, and the prediction of thrombosis from baseline variables such as markers of inflammation. SAEs or AEs will also be formally examined by the DSMB. Arrangements will be made to provide the Chair of the DSMB and possibly other members with regular information on fatalities and life-threatening SAEs possibly related to trial treatment (e.g. intracranial haemorrhage).

10. Ethical Considerations

10.1 Adherence to regulations and guidelines

This trial will adhere to the regulations, guidelines and standards espoused in the World Medical Association Declaration of Helsinki, the NHMRC National Statement of Ethical Conduct in Research Involving Humans, the joint NHMRC/AVCC Statement and Guidelines on Research Practice, and the Therapeutic Goods Administration Note for Guidance on Good Clinical Practice.

10.2 Ethics committee approvals

Patients will not be enrolled until the final approved protocol, as well as any trial information for patients is reviewed and approved by a Human Research Ethics Committee.

Correspondence with HRECs should be copied and supplied to the coordinating centre. It is the responsibility of the investigators to ensure that the appropriate person from each site is aware of the reporting responsibilities of each site to the relevant HRECs.

10.3 Justification of potential risks and benefits

The potential benefits to the patients are those relating to a reduction in AVF thrombosis rate and the associated benefits of lower hospitalisation rate, lower risk of needing alternative vascular access procedures and further attempts at establishing permanent vascular access. The only significant concern in this study is that of bleeding complications. This risk relates primarily to the group assigned to aspirin alone and to the group assigned combined aspirin and omega-3 fatty acid therapy.

The relationship between aspirin use and an increased risk of bleeding is a potential concern in patients with CKD. In the general population, the use of a chronic low dose of aspirin doubles the risk of serious gastrointestinal bleeding [33] and the theoretical risk may be higher in patients with CKD because of the presence of uraemic induced impairment of haemostasis. There have been published studies [34, 35] showing a significant elevation in bleeding times in patients on haemodialysis treatments administered a single dose of aspirin, but surprisingly little evidence-based clinical trial data in this population. This has been explored in the UK-HARP-I study. In this 2x2 factorial design study involving simvastatin and aspirin as active therapies, allocation to the aspirin group was not associated with an excess of major bleeds (2% in patients on aspirin vs. 3% in patients not on aspirin), but there was a three-fold increase in minor bleeding [36]. Importantly though, in this study, patients received aspirin for 12 months, whereas our study has a much shorter period on therapy of 3 months. In addition, patients with ESRD on dialysis undergoing renal transplantation have successfully received pre-operative aspirin for the prevention of graft thrombosis without an increased risk of major bleeding [42, 43].

Omega-3 fatty acids appear to be well tolerated, even in high doses, with gastrointestinal complaints particularly nausea, vomiting, diarrhoea and non-specific discomfort being the most often reported. In studies looking at the effect of omega-3 fatty acids on bleeding time it has not been found to be significantly prolonged [37]. In studies that have been done thus far in the CKD cohort, there have been no clear effects demonstrated in relation to platelet aggregation and bleeding times. There has only been one report of a serious bleeding event in a single patient in an uncontrolled study [38]. Furthermore, it has been reported that administration of omega-3 fatty acids can protect the gastric mucosa against aspirin-induced injury, the postulated mechanism being that omega-3 fatty acids counteract the effect of aspirin on the decrease in prostacyclin (combined effect of PGI_2 and PGI_3) [40]. This is supported by clinical data suggesting that omega-3 fatty acids decrease gastric erosions and ulcers caused by aspirin or alcohol [39]. Thus the combination of aspirin and omega-3 fatty acids may be expected to be additive in terms of efficacy in vascular access thrombosis, but to have a lower risk of bleeding complications than the use of aspirin alone.

It is important to perform this study to carefully explore the risks and benefits of aspirin and omega-3 fatty acids as single agents or as combination therapy.

10.4 Protection of patient confidentiality

Patients' records and the data generated by the study will be confidential in line with the recommendations of the NHMRC and the 2001 privacy legislation. Any information that may identify a patient will be excluded from data presented in the public arena. Data must be stored in a secure, lockable location. Electronic data storage must have adequate password protection. Standardised case report forms will be provided for each subject on this study. The participants in this study will be identified only by initials and subject number on these forms. De-identified information may only be released to the AKTN or designee.

10.5 Insurance

Insurance will be provided through the University of Queensland.

11. Trial Management

11.1 Organisational structure

The Trial Management Committee (TMC) is comprised of persons appointed through the AKTN Standard Operating Procedure. Representatives include the trial proposer (chair), one member of the AKTN Scientific Committee, study biostatistician, a member of the AKTN Operations Secretariat, and others as required. It is the responsibility of this group to provide leadership to the overall conduct of the trial. In particular, this group will review the progress of the study in achieving the objectives, take appropriate decisions to meet these objectives, and make decisions regarding the continuation or modification of a trial given reports from the Safety and Data monitoring committee.

Specifically, the TMC will:

- Monitor the blinded event rate for comparison with the one used for design purposes
- Monitor missing data
- Forward to the DSMB, on an ongoing basis, details of treatment-emergent deaths and life-threatening SAEs possibly related to treatment
- Ensure that treatment withdrawals are continuously monitored
- Communicate any notable imbalance between treatment groups to the DSMB, including details on the reason for withdrawal

The TMC oversees the trial-related activities of the coordinating office. The TMC reports to the AKTN Scientific Committee.

The Data and Safety Monitoring Board (DSMB) will be comprise of a statistician, an experienced clinical trialist and two Nephrologists who have no involvement with the day to day running of the study. The DSMB will be responsible for reviewing the interim analyses of safety (to be conducted at 1/3 and 2/3 of total patient numbers accrual), and will advise the Trial Management Committee when the stopping rules have reached or any other matters of safety that arise during the course of the study.

11.2 Publication Policy

The Australasian Kidney Trial Network subscribes to the criteria for authorship formulated by the International Committee of Medical Journal Editors [44]:

"Each author should have participated sufficiently in the work to take public responsibility for the content. Authorship credit should be based only on substantial contributions to

(a) conception and design, or analysis and interpretation of data; and to

(b) drafting the article or revising it critically for important intellectual content; and on

(c) final approval of the version to be published.

Conditions (a), (b), and (c) must all be met."

The main authorship will appear as follows:

The writing committee (listed: Chief PI listed first, then remaining members listed alphabetically) for the FAVOURED Study Collaborative Group.

On the front page, the following will be listed:

Members of the FAVOURED Study Collaborative Group are listed in the Supplementary Appendix, available with the full text of this article.

Authorship in the appendix will appear in the following order:

Writing Committee
Trial Management Committee
Steering Committee (the PI at each site)
Project Management Team (AKTN)
Data and Safety Monitoring Board
Data Management / Information Technology
All investigators and Study Coordinators at each site

The TMC will approve all publications arising from the trial before submission to journals, as will the AKTN Scientific Committee or its delegate.

Please see the AKTN Publication policy for more detail on authorship.

12. References

1. Allon, M. and M.L. Robbin, *Increasing arteriovenous fistulas in hemodialysis patients: Problems and solutions.* Kidney International, 2002. 62(4): p. 1109-1124.

2. Polkinghorne, K.R., et al., *Vascular access and all-cause mortality: a propensity score analysis.* Journal of the American Society of Nephrology, 2004. 15(2): p. 477-86.

3. Pastan, S., J.M. Soucie, and W.M. McClellan, *Vascular access and increased risk of death among hemodialysis patients.* Kidney International, 2002. 62(2): p. 620-626.

4. Excell, L., M. Marshall, and S.P. McDonald, *Haemodialysis, p35-52. In: ANZDATA Registry Report 2004*, L. Excell and S.P. McDonald, Editors. 2004, Australia and New Zealand Dialysis and Transplant Registry: Adelaide, South Australia.

5. Manns, B., et al., *Establishment and maintenance of vascular access in incident hemodialysis patients: a prospective cost analysis.* Journal of the American Society of Nephrology, 2005. 16(1): p. 201-9.

6. Port, F.K., et al., *Improving outcomes for dialysis patients in the international Dialysis Outcomes and Practice Patterns Study.* Clinical Journal of the American Society of Nephrology, 2006. 1(2): p. 246-55.

7. Ishani, A., et al., *Septicemia, access and cardiovascular disease in dialysis patients: the USRDS Wave 2 study.* Kidney International, 2005. 68(1): p. 311-8.

8. Asif, A., P. Roy-Chaudhury, and G.A. Beathard, *Early arteriovenous fistula failure: a logical proposal for when and how to intervene.* Clinical Journal of the American Society of Nephrology, 2006. 1(2): p. 332-9.

9. Rooijens, P.P.G.M., et al., *Radiocephalic Wrist Arteriovenous Fistula for Hemodialysis: Meta-analysis Indicates a High Primary Failure Rate.* European Journal of Vascular and Endovascular Surgery, 2004. 28(6): p. 583-589.

10. Marshall, M., et al., *Haemodialysis*, in *ANZDATA Registry Report 2005*. 2005, Australia and New Zealand Dialysis and Transplant Registry: Adelaide. p. 61-81.

11. Sidawy, A.N., et al., *Recommended standards for reports dealing with arteriovenous hemodialysis accesses.* Journal of Vascular Surgery, 2002. 35(3): p. 603-610.

12. Gröntoft, K.-C., et al., *Effects of Ticlopidine in AV-fistula Surgery in Uremia.* Scandinavian Journal of Urology and Nephrology, 1998. 32(4): p. 276-283.

13. Kaufman, J.S., *Antithrombotic agents and the prevention of access thrombosis.* Seminars in dialysis, 2000. 13(1): p. 40-46.

14. Andrassy, K., et al., *Prevention of p.o. clotting of av. cimino fistulae with acetylsalicyl acid. Results of a prospective double blind study.* Klin Wochenschr, 1974. 52: p. 348-349.

15. Kooistra, M.P., et al., *Low-dose aspirin does not prevent thrombovascular accidents in low-risk haemodialysis patients during treatment with recombinant human erythropoietin.* Nephrology, Dialysis, Transplantation, 1994. 9(8): p. 1115-1120.

16. Schmitz, P.G., et al., *Prophylaxis of hemodialysis graft thrombosis with fish oil: Double-blind, randomized, prospective trial.* Journal of the American Society of Nephrology, 2002. 13(1): p. 184-190.

17. Engstrom, K., R. Wallin, and T. Saldeen, *Effect of low-dose aspirin in combination with stable fish oil on whole blood production of eicosanoids.* Prostaglandins Leukotrienes and Essential Fatty Acids, 2001. 64(6): p. 291-297.

18. Khajehdehi, P., *Lipid-lowering effect of polyunsaturated fatty acids in hemodialysis patients.* Journal of renal nutrition, 2000. 10(4): p. 191-195.

19. Ando, M., T. Sanaka, and H. Nihei, *Eicosapentanoic acid reduces plasma levels of remnant lipoproteins and prevents in vivo peroxidation of LDL in dialysis patients.* Journal of the American Society of Nephrology, 1999. 10(10): p. 2177-2184.

20. Beilin, L.J. and T.A. Mori, *Dietary omega-3 fatty acids*, in *Lifestyle Modification for the Prevention and Treatment of Hypertension*, P. Whelton, J. He, and G. Louis, Editors. 2003, Marcel Dekker Inc: New York. p. 273-300.

21. Mori, T.A. and L.J. Beilin, *Omega-3 fatty acids and inflammation.* Current Atherosclerosis Reports, 2004. 6(6): p. 461-7.

22. Mori, T.A., *Effect of fish and fish oil-derived omega-3 fatty acids on lipid oxidation.* Redox Report, 2004. 9(4): p. 193-7.

23. Friedman, A. and S. Moe, *Review of the effects of omega-3 supplementation in dialysis patients.* Clinical Journal of the American Society of Nephrology, 2006. 1(2): p. 182-92.

24. Ethier, J., et al., *Aspirin prescription and outcomes in hemodialysis patients: the Dialysis Outcomes and Practice Patterns Study (DOPPS).* American journal of kidney diseases : the official journal of the National Kidney Foundation, 2007. 50(4): p. 602-11.

25. Hasegawa, T., et al., *Consistent aspirin use associated with improved arteriovenous fistula survival among incident hemodialysis patients in the dialysis outcomes and practice patterns study.* Clinical journal of the American Society of Nephrology : CJASN, 2008. 3(5): p. 1373-8.

26. Chan, K.E., et al., *Anticoagulant and antiplatelet usage associates with mortality among hemodialysis patients.* Journal of the American Society of Nephrology : JASN, 2009. 20(4): p. 872-81.

27. Dember, L.M., et al., *Effect of clopidogrel on early failure of arteriovenous fistulas for hemodialysis: a randomized controlled trial.* JAMA, 2008. 299(18): p. 2164-71.

28. Woodman, R.J., et al., *Effects of purified eicosapentaenoic acid and docosahexaenoic acid on platelet, fibrinolytic and vascular function in hypertensive type 2 diabetic patients.* Atherosclerosis, 2003. 166(1): p. 85-93.

29. Woodman, R.J., et al., *Effects of purified eicosapentaenoic and docosahexaenoic acids on glycemic control, blood pressure, and serum lipids in type 2 diabetic patients with treated hypertension.* American Journal of Clinical Nutrition, 2002. 76(5): p. 1007-1015.

30. Chan, D.C., et al., *Factorial study of the effects of atorvastatin and fish oil on dyslipidaemia in visceral obesity.* European Journal of Clinical Investigation, 2002. 32(6): p. 429-436.

31. Mori, T.A., et al., *Differential effects of eicosapentaenoic acid and docosahexaenoic acid on vascular reactivity of the forearm microcirculation in hyperlipidemic, overweight men.* Circulation, 2000. 102(11): p. 1264-1269.

32. Mori, T.A., et al., *Purified eicosapentaenoic and docosahexaenoic acids have differential effects on serum lipids and lipoproteins, LDL particle size, glucose, and insulin in mildly hypedipidemic men.* American Journal of Clinical Nutrition, 2000. 71(5): p. 1085-1094.

33. de Abajo, F.J. and L.A. Garcia Rodriguez, *Risk of upper gastrointestinal bleeding and perforation associated with low-dose aspirin as plain and enteric-coated formulations.* BMC clinical pharmacology, 2001. 1: p. 1-1.

34. Gaspari, F., et al., *Aspirin prolongs bleeding time in uremia by a mechanism distinct from platelet cyclooxygenase inhibition.* J. Clin. Invest, 1987. 79: p. 1788-1797.

35. Livio, M., G. Vigano, and A. Benigni, *Moderate doses of aspirin and risk of bleeding in renal failure.* Lancet, 1986. 1(8478): p. 414-416.

36. Baigent, C., et al., *First United Kingdom Heart and Renal Protection (UK-HARP-I) study: biochemical efficacy and safety of simvastatin and safety of low-dose aspirin in chronic kidney disease.* American Journal of Kidney Diseases, 2005. 45(3): p. 473-84.

37. Svaneborg, N., et al., *The acute and short-time effect of supplementation with the combination of n-3 fatty acids and acetylsalicylic acid on platelet function and plasma lipids.* Thrombosis Research, 2002. 105(4): p. 311-316.

38. Diskin, C.J., et al., *Fish oil to prevent intimal hyperplasia and access thrombosis.* Nephron, 1990. 55(4): p. 445-447.

39. Szabo, S. and C. Rogers, *Diet, ulcer disease, and fish oil.* Lancet, 1988. 331(8577): p. 119.

40. Al-Harbi, M.M., et al., *Effect of acute administration of fish oil (omega-3 marine triglyceride) on gastric ulceration and secretion induced by various ulcerogenic and necrotizing agents in rats.* Food and Chemical Toxicology, 1995. 33(7): p. 553-558.

41. McDonald, S.P., L. Excell, and V. Shtangey, *Method and Location of Dialysis*, in *ANZDATA Registry Report 2005*. 2005, Australia and New Zealand Dialysis and Transplant Registry: Adelaide. p. 54-60.

42. Robertson, A.J., et al., *Low dose aspirin as prophylaxis against renal-vein thrombosis in renal-transplant recipients.* Nephrol. Dial. Transplant., 2000. 15(11): p. 1865-1868.

43. Murphy, G.J., et al., *Influence of aspirin on early allograft thrombosis and chronic allograft nephropathy following renal transplantation.* British Journal of Surgery, 2001. 88(2): p. 261-266.

44. *Guidelines on authorship. International Committee of Medical Journal Editors.* British medical journal (Clinical research ed), 1985. 291(6497): p. 722.

13. Appendices

13.1 Sample Patient Information Sheet (Patients randomised to omega-3 fatty acids and aspirin)

(On institution letterhead)

(Name of Institution)

PATIENT INFORMATION SHEET

Patients taking Omega-3 Fatty Acid & Aspirin or Placebo

A randomised, double-blind, placebo-controlled, factorial-design trial to assess the effect of aspirin and fish oil (omega-3 fatty acids) in the prevention of early thrombosis in arterio-venous fistulae in patients with Stage IV or V chronic kidney disease requiring haemodialysis

FAVOURED Study (Fish oil and Aspirin in Vascular access OUtcomes In REnal Disease

Purpose of this form

This form is meant to help you to understand the information about the study. Your doctor should have already discussed the study with you. This form is intended to help you to decide whether or not to take part in the study by telling you about what is involved and what the possible risks and benefits of this study.

Purpose of the study:

The aim of the study is to find out whether aspirin or fish oil helps reduce blood clots in arterio-venous fistulae. Arterio-venous fistula or "AVF" is where an artery and vein are joined in your arm so that you are able to start haemodialysis.

Why is the study being done?

One problem which can occur with your AVF is if a blood clot forms and blocks blood flow. If this happens, it will usually happen within a few weeks of the surgery. One way to try and prevent this happening is to use medicines to prevent clots forming.

Platelets are cells in your blood which are involved in forming clots. Aspirin is well-known to have anti-platelet effects. Fish oil also has a number of effects may help stop clots forming. A combination of aspirin and fish oil may help stop clots forming better than either medication by itself.

Who will be asked to enter the study?

You will be invited to participate in the trial if you will shortly having an AVF created in your arm and are over 19 years old.

What will happen on this study?

Visit 1 - Baseline Visit (up to 4 weeks prior to surgery)

At this visit, you will be asked to provide written informed consent to participate in the study. Information will be collected such as:

- Your height
- Your weight
- Your blood pressure
- Any relevant medical history (including past use of dialysis and other medical conditions)
- Any medications you currently take

A fasting blood sample will be taken. You will be asked to complete a questionnaire about your lifestyle and dietary habits.

Visit 2 – Randomisation Visit (up to 7 days prior to surgery)

At this visit, you will be given the study medication. You will be asked to take study medication every day from the day before your AVF procedure for 3 months.

The daily medication will be

- One tablet of aspirin (100 mg) or its placebo, and
- Four capsules of fish oil (4 grams) or its placebo.

The placebo or dummy medication look just like the actual medication but contain no active ingredients. These amounts have proven to be effective in other studies.

You will be asked to take 2 capsules of fish oil and one tablet aspirin just before your breakfast and 2 capsules of fish oil just before your evening meal daily. Which type of medication you take will be decided at random (like a throw of the dice). You will have a 1 in 4 chance of receiving aspirin and fish oil together, aspirin alone, fish oil alone or just placebo. None of the study staff or any member of your usual health care team will know which medication you are taking.

While you are taking the study medication, please do not take any other medication which contains aspirin or fish oil. Medications which contain aspirin can include:

- Alka Seltzer
- Asasantin
- Aspalgin
- Aspro
- Astrix
- Cardiprin
- Cartia
- Codiphen
- Codis
- Codox
- Codral
- Disprin
- Ecotrin
- Solprin
- Veganin.

If you have a question about a medication you think may contain aspirin or fish oil, please contact your trial coordinator (insert name and phone number.)

Some herbal medications should not be taken with aspirin. So please be cautious when taking these medications while taking the study medication. It is also recommended that you limit the amount of alcohol you drink while you are taking the study medication.

Visit 3 – AVF Surgery (1 day after starting medication)

On the day of your AVF procedure, a description of the procedure will be collected including the results of any vein mapping studies undertaken prior to surgery. Details such as height, weight, blood pressure, any changes to concomitant medicine or health will be reported.

Visits 4, 5 & 6 – Treatment Visits (Weeks 1, 6 & 12)
At these visits, details about you will be recorded. This information includes:

- Your weight
- Your blood pressure
- Your use of the study medication
- Whether you are dialysing or not
- The status of your AVF
- Any changes to your health or medications you are taking

At visit 5 and 6 (weeks 6 and 12) a blood sample will be collected (you will need to fast for the week 12 sample).

Visit 7 onwards – Follow-up Visits (Month 6, Years 1, 2 and 3)
In this part of the study, you will have visits at 6 month, 1 year, 2 year and 3 years. At these visits, details such as weight, blood pressure, your dialysis and AVF status and at month 6 any changes to your health or concomitant medications will be recorded.

Throughout the study, staff will regularly check your AVF for "bruit", the sound the blood normally makes if the AVF is working properly. No other aspect of your medical care will be altered in any way. You will still be looked after by your usual health care team. A summary of all data that will be recorded is in the table below.

Study Phase	Baseline	Randomisation	Surgery	Treatment Period (wks)			Follow-up (mths)			
**Assessment**				**1**	**6**	**12**	**6**	**12**	**24**	**36**
Study medication dispensed		X								
Informed consent	X									
Medical history	X									
Weight	X		X	X	X	X	X	X	X	X
Blood pressure	X		X	X	X	X	X	X	X	X
Demographics	X									
Physical exam	X									
Dialysis details	X			X	X	X	X	X	X	X
Description and status of vascular access	X		X	X	X	X	X	X	X	X
Concomitant meds	X		X	X	X	X	X			
Adverse events	X		X	X	X	X	X			
Fasting blood test	X				X	X				

Are there any risks?

There is a large amount of research about the use of aspirin for prevention of blood clots. In general, aspirin is very good in stopping blood clots and there is only a small possibility of bleeding while taking aspirin. This study uses low dose aspirin (100mg once a day). The risk of side effects is very small at this dose. However, your doctor will keep a close eye on you and you should report any unusual bleeding immediately to your renal unit nurses or nephrologist. Symptoms that could have serious consequences include black stools, feeling faint, signs of allergic reactions and abdominal pain.

Fish oil is generally doesn't have many side effects. Gastrointestinal side effects such as nausea, vomiting, diarrhoea and non-specific discomfort are the most commonly reported.

These problems can be reduced by taking the fish oil capsules with a glass of cold water. The combination of aspirin and fish oil should have a lower risk of bleeding than just taking aspirin.

If you are a woman who is able to become pregnant, you should use an effective method of birth control for the three months that you are taking the study medication. If you do become pregnant, you should stop all study medications and inform your renal unit nurses or nephrologist immediately. The study medications are not known to affect men fathering children.

Potential Benefits

If you decide to take part in this trial, your medical condition will be very closely monitored. Any changes in your health will be very quickly identified. The results from this study may help other haemodialysis patients having AVFs in the future.

Confidentiality

All information collected for this study will be treated with complete confidentiality. You will be asked to give permission to any regulatory authorities related to the study to have access your medical records. This information will only be used for purposes of this study. Information gathered by the ANZDATA Registry (the Australia and New Zealand Dialysis and Transplant Registry) may also be used as part of this study. Medical records will be stored for accessibility for at least 15 years.

Costs

Your involvement in this study will not cost you anything. You will not be paid to take part in this study. Taking part in this study will not change usual hospital liability arrangements and processes.

Do you have a choice?

Entry into this study is entirely voluntary. Choosing not to take part in this study will not affect your treatment or care in any way. The doctors will continue to treat you with the best means available.

If you agree to participate in this study you will be asked to sign a consent form. However you may withdraw from the study at any time without giving a reason. Your doctor may also decide that you should stop if they believe it is in your best interest. You will be told if any new information arises which might affect your decision to be in the study.

For further information about this study, please contact (medical contact, research nurse contact, phone numbers).

Ethics Approval

This study has been reviewed and approved by the (local) Ethics Committee. Should you wish to discuss the matter with someone not directly involved, you can contact (Ethics Committee contact, number).

This study has been cleared by one of the human ethics committees of the University of Queensland in accordance with the National Health and Medical Research Council's guidelines. You are of course, free to discuss your participation in this study with project staff (contactable on ....). If you would like to speak to an officer of the University not involved in the study, you may contact the Ethics Officer on 07 3365 3924.

13.2 Sample Patient Information Sheet (Patients randomised to omega-3 fatty acids only)

(On institution letterhead)

(Name of Institution)

PATIENT INFORMATION SHEET

Patients taking Omega-3 Fatty Acids/Placebo and Open Label Aspirin

A randomised, double-blind, placebo-controlled, factorial-design trial to assess the effect of aspirin and fish oil (omega-3 fatty acids) in the prevention of early thrombosis in arterio-venous fistulae in patients with Stage IV or V chronic kidney disease requiring haemodialysis

FAVOURED Study (Fish oil and Aspirin in Vascular access OUtcomes In REnal Disease

Purpose of this form

This form is meant to help you to understand the information about the study. Your doctor should have already discussed the study with you. This form is intended to help you to decide whether or not to take part in the study by telling you about what is involved and what the possible risks and benefits of this study.

Purpose of the study:

The aim of the study is to find out whether aspirin and fish oil helps reduce blood clots in arterio-venous fistulae. Arterio-venous fistula or "AVF" is where an artery and vein are joined in your arm so that you are able to start haemodialysis.

Why is the study being done?

One problem which can occur with your AVF is if a blood clot forms and blocks blood flow. If this happens, it will usually happen within a few weeks of the surgery. One way to try and prevent this happening is to use medicines that may prevent the clot forming.

Platelets are cells in your blood which are involved in forming clots. Aspirin is well-known to have anti-platelet effects. Fish oil also has a number of effects may help stop clots forming. A combination of aspirin and fish oil may help stop clots forming better than either medication by itself.

Who will be asked to enter the study?

You will be invited to participate in the trial if you will shortly having an AVF created in your arm and are over 19 years old.

What will happen on this study?

Visit 1 - Baseline Visit (up to 4 weeks prior to surgery)

At this visit, you will be asked to provide written informed consent to participate in the study. Information will be collected such as:

- Your height
- Your weight
- Your blood pressure
- Any relevant medical history (including past use of dialysis and other medical conditions)
- Any medications you currently take

A fasting blood sample will be taken. You will be asked to complete a questionnaire about your lifestyle and dietary habits.

Visit 2 – Randomisation Visit (up to 7 days prior to surgery)

At this visit, you will be given the study medication. You will be asked to take study medication every day from the day before your AVF procedure for 3 months. The daily medication will be four capsules of fish oil (4 grams) or its placebo. The placebo or dummy medication look just like the fish oil capsules but contain no active ingredients. This amount has been proven to be effective in other studies.

You will be asked to take 2 capsules of fish oil just before your breakfast and 2 capsules of fish oil just before your evening meal daily. Which type of medication you take will be decided at random (like a throw of the dice). You will have a 1 in 2 chance of receiving fish oil alone or placebo. None of the study staff or any member of your usual health care team will know which medication you are taking.

While you are taking the study medication, please do not take any other medication which contains fish oil. Please inform the study staff if you take any medications containing aspirin other than your regular low dose aspirin including:

- Alka Seltzer
- Asasantin
- Aspalgin
- Aspro
- Astrix
- Cardiprin
- Cartia
- Codiphen
- Codis
- Codox
- Codral
- Disprin
- Ecotrin
- Solprin
- Veganin.

If you have a question about a medication you think may contain aspirin or fish oil, please contact your trial coordinator (insert name and phone number.)

Some herbal medications should not be taken with aspirin. So please be cautious when taking these medications while taking the study medication. It is also recommended that you limit the amount of alcohol you drink while you are taking the study medication.

Visit 3 – AVF Surgery (1 day after starting medication)

On the day of your AVF procedure, a description of the procedure will be collected including the results of any vein mapping studies undertaken prior to surgery. Details such as height, weight, blood pressure, any changes to concomitant medicine or health will be reported.

Visits 4, 5 & 6 – Treatment Visits (Weeks 1, 6 & 12)
At these visits, details about you will be recorded. This information includes:

- Your weight
- Your blood pressure
- Your use of the study medication
- Whether you are dialysing or not
- The status of your AVF
- Any changes to your health or medications you are taking

At visit 5 and 6 (weeks 6 and 12) a blood sample will be collected (you will need to fast for the week 12 sample).

Visit 7 onwards – Follow-up Visits (Month 6, Years 1, 2 and 3)
In this part of the study, you will have visits at 6 month, 1 year, 2 year and 3 years. At these visits, details such as weight, blood pressure, your dialysis and AVF status and at month 6 any changes to your health or concomitant medications will be recorded.

Throughout the study, staff will regularly check your AVF for "bruit", the sound the blood normally makes if the AVF is working properly. No other aspect of your medical care will be altered in any way. You will still be looked after by your usual health care team. A summary of all data that will be recorded is in the table below.

Study Phase	Baseline	Randomisation	Surgery	Treatment Period (wks)			Follow-up (mths)			
**Assessment**				**1**	**6**	**12**	**6**	**12**	**24**	**36**
Study medication dispensed		X								
Informed consent	X									
Medical history	X									
Weight	X		X	X	X	X	X	X	X	X
Blood pressure	X		X	X	X	X	X	X	X	X
Demographics	X									
Physical exam	X									
Dialysis details	X			X	X	X	X	X	X	X
Description and status of vascular access	X		X	X	X	X	X	X	X	X
Concomitant meds	X		X	X	X	X	X			
Adverse events	X		X	X	X	X	X			
Fasting blood test	X				X	X				

<u>Are there any risks?</u>
There is a large amount of research about the use of aspirin for prevention of blood clots. In general, aspirin is very good in stopping blood clots and there is only a small possibility of bleeding while taking aspirin. However, your doctor will keep a close eye on you and you should report any unusual bleeding immediately to your renal unit nurses or nephrologist. Symptoms that could have serious consequences include black stools, feeling faint, signs of allergic reactions and abdominal pain.

Fish oil is generally doesn't have many side effects. Gastrointestinal side effects such as nausea, vomiting, diarrhoea and non-specific discomfort are the most commonly reported. These problems can be reduced by taking the fish oil capsules with a glass of cold water. The combination of aspirin and fish oil should have a lower risk of bleeding than just taking aspirin.

If you are a woman who is able to become pregnant, you should use an effective method of birth control for the three months that you are taking the study medication. If you do become pregnant, you should stop all study medications and inform your renal unit nurses or nephrologist immediately. The study medications are not known to affect men fathering children.

Potential Benefits

If you decide to take part in this trial, your medical condition will be very closely monitored. Any changes in your health will be very quickly identified.

The results from this study may help other haemodialysis patients having AVFs in the future.

Confidentiality

All information collected for this study will be treated with complete confidentiality. You will be asked to give permission to any regulatory authorities related to the study to have access your medical records. This information will only be used for purposes of this study. Information gathered by the ANZDATA Registry (the Australia and New Zealand Dialysis and Transplant Registry) may also be used as part of this study. Medical records will be stored for accessibility for at least 15 years.

Costs

Your involvement in this study will not cost you anything. You will not be paid to take part in this study. Taking part in this study will not change usual hospital liability arrangements and processes.

Do you have a choice?

Entry into this study is entirely voluntary. Choosing not to take part in this study will not affect your treatment or care in any way. The doctors will continue to treat you with the best means available.

If you agree to participate in this study you will be asked to sign a consent form. However you may withdraw from the study at any time without giving a reason. Your doctor may also decide that you should stop if they believe it is in your best interest. You will be told if any new information arises which might affect your decision to be in the study.

For further information about this study, please contact (medical contact, research nurse contact, phone numbers).

Ethics Approval

This study has been reviewed and approved by the (local) Ethics Committee. Should you wish to discuss the matter with someone not directly involved, you can contact (Ethics Committee contact, number).

This study has been cleared by one of the human ethics committees of the University of Queensland in accordance with the National Health and Medical Research Council's guidelines. You are of course, free to discuss your participation in this study with project staff (contactable on ....). If you would like to speak to an officer of the University not involved in the study, you may contact the Ethics Officer on 07 3365 3924.

13.3 Sample Patient Consent Form

(Name of institution)

Consent Form

A randomised, double-blind, placebo-controlled, factorial-design trial to assess the effect of aspirin and fish oil (omega-3 fatty acids) in the prevention of early thrombosis in arterio-venous fistulae in patients with Stage IV or V chronic kidney disease requiring haemodialysis

- **FAVOURED Study (Fish oil and Aspirin in Vascular access OUtcomes In REnal Disease**

- I agree to participate in the above named clinical trial.
- I have read and understood and kept a copy of the Patient Information Sheet.
- I acknowledge and understand the methods and demands relating to the study and the possible risks and inconveniences which may occur.
- I have had my questions answered to my satisfaction.
- I understand that I may withdraw from the study at any time without giving any reason and that this will not affect my medical treatment.
- I give my permission for access to my medical records including information gathered by the ANZDATA Registry (the Australia and New Zealand Dialysis and Transplant Registry) for the purposes of this research.
- I give my permission for access to my medical records for the purposes of this research, including the pre-surgical Doppler vein mapping.

Patient's signature Printed name Date

(to be personally dated)

Witness signature Printed name Date

(to be personally dated)

I have explained the nature and purpose of this study to the above participant and have answered their questions.

Investigator's signature Printed name Date

13.4 Classification of Chronic Kidney Disease

The Kidney Disease Outcomes Quality Initiative (K/DOQI) of the National Kidney Foundation (NKF) defines chronic kidney disease (CKD) as either kidney damage or a decreased kidney glomerular filtration rate (GFR) of <60 mL/min/1.73 m^2 for 3 or more months. February 2002 K/DOQI classification of the stages of CKD:

- Stage 1: Kidney damage with normal or increased GFR (>90 mL/min/1.73 m^2)
- Stage 2: Mild reduction in GFR (60-89 mL/min/1.73 m^2)
- Stage 3: Moderate reduction in GFR (30-59 mL/min/1.73 m^2)
- Stage 4: Severe reduction in GFR (15-29 mL/min/1.73 m^2)
- Stage 5: Kidney failure (GFR <15 mL/min/1.73 m^2 or dialysis)

13.5 Lifestyle questionnaire

LIFESTYLE QUESTIONNAIRE

Fish Oil and Aspirin in the Prevention of Thrombosis in Chronic Kidney Disease

Thank you for your interest in participating in this research trial. We would very much appreciate your help in filling in this questionnaire, which will give us information about your lifestyle, including diet and exercise. Once you have completed the questionnaire please return it to the supervising research staff member.

The questionnaire should take approximately 15 minutes to complete.

Please take your time and answer all questions as accurately as possible.

N.B. ALL INFORMATION WILL REMAIN STRICTLY CONFIDENTIAL

**Today's Date** (dd/mm/yyyy)	_______ / _______ / ____________
**Patient's Initials**	_______ _______ _______
**Date of Birth** (dd/mm/yyyy)	_______ / _______ / ____________

YOUR DRINKING DETAILS

1. Do you drink alcohol? (Circle one)

I have never drunk alcohol (go to Question 4) . . 1
I used to drink alcohol (go to Question 2) . . . 2
I occasionally drink alcohol (go to Question 3) . . 3

2. If you gave up drinking alcohol, how long ago did you stop? (Circle one)

Less than 3 months ago 1
3 - 6 months ago 2
More than 6 months but less than 1 year ago . . 3
More than 1 year ago 4
(go to Question 4)

3. How many standard drinks (see below) of alcohol do you usually drink? (Circle one)

	Less than 1 per week	1 - 6 per week	1 per day	2-3 per day	4 per day	More than 4 per day
A. Beer	1	2	3	4	5	6
B. Wine	1	2	3	4	5	6
C. Spirits	1	2	3	4	5	6

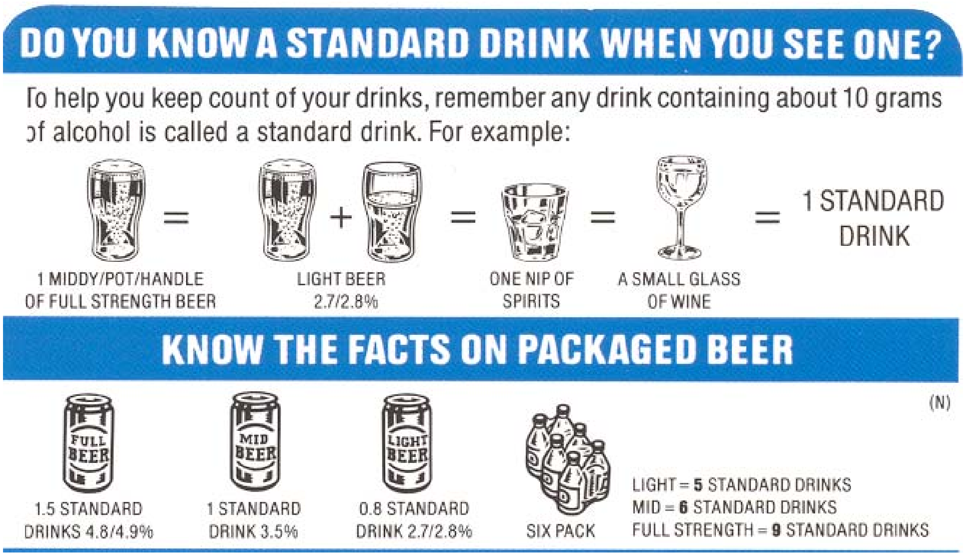

YOUR SMOKING HISTORY

4. Do you smoke cigarettes, cigars, pipes or other tobacco products? (Circle one)

I have never smoked (go to Question 7) . . .	1
I used to smoke (go to Question 5)	2
I am currently a smoker (go to Question 6) . . .	3

5. If you gave up smoking, about how long ago did you stop? (Circle one)

Less than 3 months ago	1
3 - 6 months ago	2
More than 6 months but less than 1 year ago . .	3
More than 1 year ago	4 (go to Question 7)

6. If you smoke, how many cigarettes, cigars, etc do you smoke per day on average? _______ per day

YOUR LEVEL OF PHYSICAL ACTIVITY

7. How often do you exercise (includes taking walks, gardening or playing sport)? (Circle one)

Daily	1
3 – 6 times per week	2
1 - 2 times per week	3
Monthly	4

8. How long, on average, are you active for on these days? _______ min per day

YOUR DIET

9. How many serves of <u>vegetables</u> or salad do you eat <u>per day</u> on average? (A serve is half-a cup) _______ per day

10. How many serves of <u>fruit</u> do you eat <u>per day</u> on average? (A serve is half-a cup or the equivalent of one medium apple) _______ per day

11. How many times <u>per week</u> do you eat <u>fried food</u>? _______ per week

12. How many times <u>per week</u> do you eat <u>pies, cakes, biscuits or chocolate</u>? _______ per week

13. How often do you eat <u>fish</u> on average? (Circle one)

Less than once per month	1
1 - 3 times per month	2
Once per week	3
2 - 4 times per week	4
More than 5 times per week	5

14. What <u>type of fish</u> do you usually eat? (Circle one)

Fish, grilled, steamed, or baked	1
Fish, fried	2
Fish, canned (include tuna, salmon, sardines, mackerel) .	3
Shellfish (include prawns, scallops, crayfish) . .	4

15. If you have recently taken <u>fish oil capsules</u>, on average, how many did you take? _______ per day

16. How often do you eat <u>red meat</u> on average? (Circle one)

Less than once per month	1
1 - 3 times per month	2
Once per week	3
2 - 4 times per week	4
More than 5 times per week	5

17. When you <u>red eat</u> meat do you remove the fat? (Circle one)

Usually	1
Sometimes	2
Never / rarely	3

18 Do you add <u>salt</u> to food at the table? (Circle one)

Usually	1
Sometimes	2
Never / rarely	3

19. How often do you eat <u>chicken</u> on average? (Circle one)

Less than once per month	1
1 - 3 times per month	2
Once per week	3
2 - 4 times per week	4
More than 5 times per week	5

20. When you eat chicken do you remove the skin? (Circle one)

Usually	1
Sometimes	2
Never / rarely	3

21. What kind of <u>milk</u> do you usually use? (Circle one)

Full cream	1
Reduced fat	2
High calcium	3
Skim	4
Soy	5
Other.	6

If other, please specify____

22. How much milk do you consume per day?

1 cup of coffee or tea has the equivalent of 1 tablespoon of milk _______ tablespoons per day

1 bowl of cereal has the equivalent of ½ cup of milk _______ cups per day

<u>YOUR FAMILY HISTORY</u>

23. Did your father have cardiovascular disease (stroke or other disease affecting the heart or blood vessels) before he was 45 years old? (Circle one)

Yes	1
No	2
Don't know.	3

24. Did your mother have cardiovascular disease before she was 55 years old? (Circle one)

Yes	1
No	2
Don't know.	3

THANK YOU FOR YOUR CO-OPERATION

13.6 Data related to patency rates

Table: Studies comparing long-term patency AVF versus AVG.

Study	Study Type	Country	Access Type	Number	1° Failure (%)	1° Patency 1yr (%)	1° Patency 2yr (%)	1° Patency 3yr (%)	1° failure included	Intervention Rate (/acc-yr)
Palder 1985	R,S	USA	RCF	154	24	60*	50*	42*	Y	**
			AVG	163	NS	78*	70*	56*	Y	**
Winsett 1985	R,S	USA	RCF	273	27			72 (84*)	N	0.06
			AVG	202	NS			39 (70*)	N	0.44
Kherlakian 1986	R,S	USA	RCF	100	12	71*	66*	64*	Y	***
			AVG	100	4	75*	61*	50*	Y	***
Zibari 1988	R,S	USA	AVF	160						***
			AVG	166						***
Coburn 1994	R,S	USA	Basilic	59	NS	90 (90*)	86 (86*)		NS	***
			AVG	47	NS	70 (87*)	49 (64*)		NS	***
Burger 1995	P,S^	Netherlands	RCF	208	NS	53 (79*)	36 (68*)	24 (59*)	NS	NS
			AVG	46	NS	37 (61*)	21 (60*)	17 (54*)	NS	NS
Enzler 1996	R,S	Switzerland	RCF	429	NS	70 (74*)	61 (67*)	59 (64*)	NS	**
			AVG	69	NS	41 (58*)	24 (47*)	24 (40*)	NS	**
Rocco 1996	R,S	USA	RCF	48	31	55 (78*)			NS	0.34
			AVG	40	12	60 (88*)			NS	0.8
Miller 1997	R,S	USA	AVF	75	NS	84 (83*)	74 (70*)		NS	0.17
			AVG	23	NS	47 (73*)	38 (58*)		NS	1.17
Hodges 1997	R,S	USA	AVF	87	31	43 (46*)			Y	0.07
			AVG	236	NS	41 (59*)			Y	0.5
Silva 1998	P,S	USA	AVF	108	8	83			Y	NS
			AVG	52	8	74			Y	NS
Matsuura 1998	R,S	USA	Basilic	30	NS		70 (70*)		NS	**
			AVG	68	NS		46 (51*)		NS	**
Kalman 1999	P,S	Canada	AVF	235	12		54 (70*)		Y	**
			AVG	231	NS		18 (60*)		Y	**
Ascher 2000	R,S	USA	AVF	99	18	84			N	0.07
			AVG	122	NS	54			N	0.74

Study	Study Type	Country	Access Type	Number	1° Failure (%)	1° Patency 1yr (%)	1° Patency 2yr (%)	1° Patency 3yr (%)	1° failure included	Intervention Rate (acc/yr)
Staramos 2000[+]	R,S	Greece	AVF	68	NS	67 (67*)	52 (52*)	44 (44*)	Y	NS
			AVG	67	NS	66 (81*)	53 (65*)	38 (58*)	Y	NS
Gibson 2001b	P, M	USA	AVF	492	NS	56 (73*)	40 (64*)		NS	1.0[c]
			VTAVF	181	NS	44 (68*)	28 (60*)		NS	1.32[c]
			AVG	1574	NS	38 (72*)	25 (60*)		NS	1.91[c]
Allon 2001	R,S	USA	AVF	139	46	42 (44*)			Y	0.57
			AVG	78	21	43 (48*)			Y	1.67
Gibson 2001a	R,S	USA	AVF	130	23	56 (72*)	41 (61*)		Y	**
			AVG	92	NS	36 (58*)	11 (41*)		Y	**
Oliver 2001	R,S	USA	BCF	56	32	64			N	0.4
			Basilic	59	21	64			N	0.7
			AVG	80	15	62			N	2.4
Dixon 2002	R,S	USA	RCF	88	32	44 (52*)	40 (48*)	31 (43*)	Y	1.4
			BCF	117	28	62 (69*)	48 (59*)	37 (53*)	Y	2.71
			AVG	117	22.6	27 (54*)	13 (42*)	7 (31*)	Y	3.84
Pisoni 2002	P,M	USA	AVF	177	NS	68			N	NS
			AVG	251	NS	49			N	NS
Lawrence 2002	R,S	Australia	AVF	64	NS	87	87		NS	NS
			AVG	26	NS	63	50		NS	NS
Shenoy 2003	R,M	USA	AVF [#]	242	17		54 (67*)		Y	0.22
			AVG [#]	440	17		36 (39*)		Y	0.86
			AVF [##]	276	28		34 (48*)		Y	0.37
			AVG [##]	384	28		17 (19*)		Y	1.73
Fischer 2003	R,S	Australia	RCF	147	NS	69*	63*	55*	NS	**
			AVG	50	NS	89*	75*	68*	NS	**
Choi 2003	R,S	USA	BCF	30	27	53 (67*)	34 (56*)		Y	**
			Basilic	42	5	76 (83*)	68 (75*)		Y	**
			AVG	53	NS	47 (59*)	26 (40*)		Y	**

Study	Study Type	Country	Access Type	Number	1o Failure (%)	1o Patency 1yr (%)	1o Patency 2yr (%)	1o Patency 3yr (%)	1o failure included	Intervention Rate (acc/yr)
Huber 2003	Meta	NA	AVF ###	1849	NS	72[a] (86*)	51[b] (77*)		NS	NS
			AVG	1245	NS	58[a] (76*)	33[b] (55*)		NS	NS
Perara 2004	R,S	USA	AVF	100	11%	56 (64*)	39 (53*)		Y	0.53
			AVG	131	NS	36 (65*)	9 (46*)		Y	0.92
Kawecka 2005	R,S	Poland	RCF	540	NS	52 (67*)	41 (56*)		NS	NS
			BCF	143	NS	45 (61*)	31 (44*)		NS	NS
			Basilic	85	NS	42 (54*)	28 (38*)		NS	NS
			AVG	86	NS	48 (56*)	21 (33*)		NS	NS
Fitzgerald 2005	R,S	USA	UAAVF	86	NS	50	46		NS	**
			AVG	60	NS	50	37		NS	**

Table from Polkinghorne, unpublished

Footnotes:

1° = Primary
AVF = native arteriovenous fistula
RCF = Radiocephalic AVF
BCF = Brachiocephalic AVF
Basilic = Brachiobasilic AVF
UAAVF = Upper Arm AVF (either BCF or Brachiobasilic AVF)
AVG = arteriovenous (PTFE) graft
VTAVF = Venous transposition AVF
R = Retrospective
P = Prospective
S = Single centre
M = multicentre
Meta = meta-analysis
Y = Yes

N = No
NS = Not stated
NA = Not applicable
* Indicates secondary patency,
** Overall rates not given however significantly more thrombectomies and revisions in the AVG group
*** Overall rates not given however significantly more complications seen in the AVG group
^ Retrospective 1971-1980, prospective 1980-1991
# Clips used
Sutures used
Upper arm AVF only + only patients 70 years and over
[a] 6 month patency
[b] 18 month patency
[c] Incident Rate Ratio, AVF comparison group VTAVF vs AVG p=0.04, AVG vs AVF p<0.01
