# Supplementary material for: Fish oil and aspirin effects on arteriovenous fistula function: Secondary outcomes of the randomised omega-3 fatty acids (Fish oils) and Aspirin in Vascular access OUtcomes in REnal Disease (FAVOURED) trial
Source: PLoS One. 2019 Mar 26;14(3):e0213274. doi: 10.1371/journal.pone.0213274 (PMC6435148; doi:10.1371/journal.pone.0213274)
Supplement: S2 File — (PDF) [file pone.0213274.s002.pdf]

STATISTICAL ANALYSIS PLAN
(Version 1.11)

Protocol Title: A randomised, double-blind, placebo-controlled factorial-design trial to assess the effect of aspirin and fish oil (omega-3 fatty acids) in the prevention of early thrombosis in arterio-venous fistulae in patients with stage IV or V chronic kidney disease requiring haemodialysis (Version 9)

Short title: FAVOURED (Fish oil and Aspirin in Vascular access OUtcomes in REnal Disease).

Protocol Date: 30 November 2010

Trial Registration: Australian New Zealand Clinical Trials Registry number ACTRN12607000569404

Australasian Kidney Trials Network

Princess Alexandra Hospital, 199 Ipswich Road, Woolloongabba QLD 4102

DOCUMENT HISTORY

Version	Reason(s) for change	Date
V1.11	Initial document	2 September 2015

APPROVALS

Principal Investigator & Chair of Trial Management Committee

Name	Signature	Date
Prof Ashley Irish		2 September 2015

Statistician, Australasian Kidney Trials Network

Name	Signature	Date
Ms Elaine Pascoe		2 September 2015

Chair, Executive Operations Secretariat, Australasian Kidney Trials Network

Name	Signature	Date
A/Prof Carmel Hawley		2 September 2015

This *Statistical Analysis Plan is* the property of the University of Queensland through the Australasian Kidney Trials Network. No section of this document may be copied, published, or used for any purpose without appropriate acknowledgement and the prior written permission of the Australasian Kidney Trials Network.

PREFACE

This Statistical Analysis Plan (SAP) describes the planned analyses and reporting for the Australasian Kidney Trials Network (AKTN) trial number 06.01, “A randomised, double-blind, placebo-controlled factorial-design trial to assess the effect of aspirin and fish oil (omega-3 fatty acids) in the prevention of early thrombosis in arterio-venous fistulae in patients with stage IV or V chronic kidney disease requiring haemodialysis: The FAVOURED Study”. Version nine of the protocol details revisions to the design and primary outcome and, while the trial continued to be referred to as a two-by-two factorial design, the revised primary objective focussed solely on omega-3 PUFAs and the trial design associated with this objective is two parallel groups (omega-3 PUFAs versus matching placebo) stratified by aspirin use (no aspirin, randomised to aspirin, open-label aspirin) (1).

The structure and content of this SAP provide sufficient detail to meet the requirements identified by the International Conference on Harmonisation of Technical Requirements for Registration of Pharmaceuticals for Human Use (ICH): Guidance on Statistical Principles in Clinical Trials (2). All work planned and reported for this SAP will follow national and international guidelines for statistical practice (3-6).

The planned analyses identified in this SAP will be included in future manuscripts. Exploratory analyses not necessarily identified in this SAP may be performed to support planned analyses. Any post-hoc or unplanned analyses not specified in this SAP will be clearly identified as such in the Final Statistical Report (FSR) and manuscripts for publication.

This SAP was written by clinical investigators and statisticians and other trial staff who were blinded to treatment allocation and treatment-related study results and will remain so until the central database is locked and the final data are extracted for analysis. To ensure blinding, treatment allocations are stored in a separate location accessible only by the designated un-blinded AKTN statistician.

The following documents were reviewed when preparing this SAP:

- Clinical Research Protocol for AKTN Trial Number 06.01 (7).
- Case report forms (CRFs) for AKTN Trial Number 06.01.
- Charter for the Data Safety Monitoring Board (DSMB) for the FAVOURED trial (8).
- ICH Guidance on Statistical Principles for Clinical Trials (2).
- ICH Guidance on Structure and Content of Clinical Study Reports (9).

Readers of this SAP are encouraged to read the Clinical Research Protocol for details on the conduct of this study and the timing of clinical assessments.

ABBREVIATIONS

ABBREVIATION	DEFINITION
ADR	Adverse Drug Reaction
AKTN	Australasian Kidney Trials Network
AVF	Arteriovenous Fistula
AVG	Arteriovenous Graft
CAP	Cannulation Assessment Period
CKD	Chronic Kidney Disease
CVC	Central Venous Catheter
DSMB	Data and Safety Monitoring Board
ESKD	End Stage Kidney Disease
FSR	Final Statistical Report
HD	Haemodialysis
ICH	International Conference on Harmonisation
ITT	Intention-To-Treat
NSAID	Non-steroidal anti-inflammatory drug
Omega-3 PUFAs	Omega-3 Polyunsaturated Fatty Acids
SAE	Serious Adverse Event
SAP	Statistical Analysis Plan
TMC	Trial Management Committee
WHO	World Health Organisation

TABLE OF CONTENTS

DOCUMENT HISTORY 2

APPROVALS 2

PREFACE 3

ABBREVIATIONS 4

TABLE OF CONTENTS 5

1. INTRODUCTION 7

1.1 Background 7

1.2 Study synopsis 7

1.3 Sub-studies 8

2. STUDY DESIGN ISSUES 8

2.1 Overview 8

2.2 Study population 9

2.2.1 Inclusion criteria 9

2.2.2 Exclusion criteria 9

2.3 Study design and treatment allocation 10

2.4. Sample size 10

2.5 Treatment blinding and allocation concealment 11

2.6 Schedule of assessments 11

3. STUDY OUTCOME VARIABLES 12

3.1 Primary outcome 12

3.2 Secondary outcomes 12

3.3 Tertiary outcomes 13

3.4 Safety outcomes 14

4. SEQUENCE OF PLANNED ANALYSES 14

4.1 Interim analyses 14

4.2 Blinded reviews of primary outcome 14

4.3 Planned intervention group comparisons 14

4.3.1 Primary outcome 14

4.3.2 Secondary outcomes 15

4.3.3 Tertiary outcomes 15

4.3.4 Safety outcomes 15

4.4 Final analyses and reporting 15

5. CHANGES TO STATISTICAL INFORMATION IN THE PROTOCOL 16

6. ANALYSIS PRINCIPLES *16*

6.1 Intention-to-treat principle and analysis dataset **16**

6.2 Multicentre study and heterogeneity **17**

6.3 Multiple comparisons and multiplicity **17**

6.4 Covariate adjustment of main analyses **17**

7. STATISTICAL METHODS *18*

7.1 Analysis of the primary outcome **18**

7.1.1 Primary comparison 18

7.1.2 Supporting and sensitivity analyses 18

7.1.3 Subgroup analyses 19

7.1.4 Other comparisons 19

7.2 Analysis of secondary outcomes **20**

7.3 Analysis of tertiary outcomes **20**

7.4 Analysis of safety outcomes **20**

7.5 Data manipulation and computing **21**

8. REPORTING *21*

8.1 Trial profile **21**

8.2 Patient characteristics and baseline comparisons **21**

8.3 TABLES, LISTINGS, AND FIGURES (TLFs) **22**

8.3.1 Planned tables 22

8.3.2 Planned listings 23

8.3.3 Planned figures 23

8.3.4 Supplementary TLFs 23

8.4 General reporting conventions **23**

8.5 Statistical summary conventions **24**

9. REFERENCES *24*

1. INTRODUCTION

1.1 Background

The incidence and prevalence of end-stage kidney disease (ESKD) are increasing due to aging of the population and growing rates of co-morbid conditions such as diabetes mellitus. Haemodialysis (HD) is the most common renal replacement therapy worldwide and a functioning vascular access is essential for optimal patient outcomes. Vascular access dysfunction is associated with significant morbidity and mortality and presents a major economic burden for health care providers (10-12). A native arteriovenous fistula (AVF) is the preferred type of vascular access due to lower rates of thrombosis, infection, interventions to maintain patency and overall mortality when compared with synthetic arteriovenous grafts (AVG) or central venous catheters (CVC) (13-15). However, native AVF take longer to establish and have a higher risk of failure to mature.

Primary failure rates of AVF have been reported to range from 20% to 60% with failure usually occurring as a result of early thrombosis or failure of the fistula to mature (16, 17). Strategies to improve early access survival by reducing thrombosis include short-term anti-platelet therapy and pre-operative identification by ultrasound of unsuitable anatomy. Although several small trials have assessed the effect of short-term post-operative use of antiplatelet agents such as aspirin, sulphinpyrazone, and ticlopidine (18-22) on early AVF thrombosis, these trials had many limitations including inadequate power, variable drug dosing and variable timing of drug administration. To date, there has been very little work focused on the important area of interventions to promote AVF maturation. Omega-3 polyunsaturated fatty acids (omega-3 PUFAs) found in fish oils exert biological effects that may improve both AVF maturation and patency due to pleiotropic biological actions, including inhibition of platelet aggregation, vasodilatation, and anti-inflammatory and anti-proliferative effects by reducing the availability of arachidonic acid, leukotriene and cytokine production as well as increasing prostaglandin-13 production (23). Whilst fish oil reduces thrombosis rates in synthetic *grafts* when compared to placebo (24), there has been no trial to date examining the effect of fish oils on AVF outcomes.

1.2 Study synopsis

The FAVOURED trial is an international multi-centre, double-blind, randomised controlled trial designed to determine whether the use of omega-3 PUFAs when compared with matched placebo would reduce the AVF access failure rate in the 12 months following AVF surgery. The trial has more arms than needed to test the primary question because of midstream revisions of inclusion criteria to allow randomisation of patients taking aspirin as well as changes to the primary objective and outcome to focus on the effect of omega-3 PUFAs on a composite measure of AVF access failure (1).

Figure 1 displays the study schema. Eligible patients were classified according to their current aspirin-taking status. Patients who were taking aspirin and unable to cease (open-label aspirin) were randomly assigned to receive either omega-3 PUFAs or placebo capsules. Patients in the omega-3 PUFAs group received 4 gm daily in the form of four Omacor capsules (46% eicosapentaenoic acid (EPA) and 38% docosapentaenoic acid (DHA), as the ethyl esters). Patients in the placebo group received 4 matching placebo capsules (olive oil). Both active and placebo capsules were supplied by BGP Products Operations GmBH.

Patients who were either not taking aspirin or were taking aspirin but could stop were randomly assigned to one of four groups: omega-3 PUFAs plus low-dose oral aspirin (100 mg per day); omega-3 PUFAs plus matching placebo aspirin; placebo omega-3 PUFAs plus low-dose oral aspirin; or placebo omega-3 PUFAs plus placebo aspirin. Active and placebo aspirin tablets were supplied by Bayer Healthcare. For all groups, treatment commenced on the day prior to surgery and continued for 12 weeks.

To address the primary research question, two groups were formed by combining the three groups given omega-3 PUFAs (arms A, B, E) and the three groups given placebo omega-3 PUFAs (arms C, D, F). Aspirin use (no aspirin, randomised to aspirin, open-label aspirin) was expected to be balanced across the two combined omega-3 PUFAs groups (active and placebo) due to the separate randomisation schemes, one allocating approximately equal numbers of existing aspirin users to the two omega-3 PUFAs groups and the other allocating approximately equal numbers of non-aspirin users to the four groups in the original factorial design.

The planned sample size was 906 patients (includes adjustment for anticipated rates of treatment drop-out and non-compliance). Allowing for a 5% loss to follow-up, the recruitment target was 954 patients. The trial began recruiting participants in August 2008 and stopped recruiting on 28 February 2014, before reaching the recruitment target. A total of 568 participants from 35 clinical centres in Australia, New Zealand, Malaysia and the United Kingdom were randomised, which corresponds to approximately 60% of the planned recruitment target. The central database is expected to be ready for analysis by July 2015.

1.3 Sub-studies

There are three sub-studies associated with the FAVOURED trial: the red blood cell (RBC) membrane fatty acid sub-study; the dietary and physical questionnaire sub-study; and the pre-surgical doppler sub-study. The RBC fatty acid sub-study will assess compliance in use of omega-3 fatty acids and the association between RBCs and clinical and dietary baseline characteristics. The dietary and physical questionnaire sub-study will examine the relationship between dietary intake (especially fish) and lifestyle factors and their association with patient outcomes. The pre-surgical Doppler sub-study will examine relationships between pre-operative vascular characteristics determined by duplex ultrasound and AVF access outcomes. Analysis plans for these sub-studies will be documented separately. A fourth sub-study is described in the trial protocol, the platelet and coagulation function sub-study, but it was abandoned due to difficulties in data collection.

2. STUDY DESIGN ISSUES

2.1 Overview

The study is a prospective, multi-centre, double-blind, randomised, placebo-controlled phase-three trial.

2.2 Study population

The population of interest is chronic kidney disease (CKD) patients attending renal units (outpatients and dialysis) throughout Australia, New Zealand, Malaysia and the United Kingdom who have a planned AVF surgery.

2.2.1 Inclusion criteria

Patients were eligible for inclusion in the study if all the following criteria were met:

1. Stage 4 or 5 CKD
2. Currently on haemodialysis or haemodialysis is planned to start within 12 months (including patients currently on peritoneal dialysis)
3. Planned AVF will be the primary haemodialysis access mechanism
4. Surgery to create an AVF in the upper or lower arm is planned
5. Aged over 19 years
6. Treating team agreeable to patient's involvement in the trial
7. Able to give informed consent

2.2.2 Exclusion criteria

Patients were excluded from the study if any one of the following conditions was met:

1. Revision of existing AVF rather than de novo AVF
2. Medical indication for anticoagulants or anti-platelet agents*****
3. Known intolerance of agents including hypersensitivity to aspirin, allergy to any other nonsteroidal anti-inflammatory drug (NSAID) or fish
4. Current use of aspirin within two weeks of commencing the trial, or of omega-3 PUFAs within 4 weeks of commencing the trial*****
5. Pregnancy, lactation or intention to fall pregnant during the time course of the study
6. Known bleeding disorder or established diagnosis of active or suspected bleeding
7. History of gastrointestinal ulcers or bleeding within the last 3 months
8. Platelet count less than 100 x 10^9 /L
9. Known active peptic ulcer disease
10. Severe hepatic insufficiency
11. Already receiving anti-coagulation therapy such as warfarin
12. Receiving regular NSAID treatment for another indication such as arthritis
13. Syndrome of asthma, rhinitis and nasal polyps if uncontrolled on usual therapy
14. Plan to have other (non-access) surgery within 2 weeks of trial medication period where, in the opinion of the investigator, aspirin or omega-3 PUFAs would be contraindicated for the planned procedure
15. Potential non-compliance with treatment regimen in the view of the treating clinicians
16. Involved in another clinical trial where the intervention being trialled is likely to confound the outcome of this trial
17. Previously randomised to this trial

*****These criteria were exclusions under the original protocol. Under the amended protocol, patients who met exclusion criterion 2 (Medical indication for anticoagulants or anti-platelet agents) due to aspirin use and/or exclusion criterion 4 (Current use of aspirin within two weeks of commencing trial) but were otherwise suitable were randomised to one of the open-label aspirin arms.

2.3 Study design and treatment allocation

Before randomisation, participants were classified according to their aspirin-taking status: (1) not taking aspirin or taking aspirin but able to cease; (2) taking aspirin and unable to cease. Those in the first category were randomised 1:1:1:1 to one of four arms in a factorial design: active aspirin and active omega-3 PUFAs; active aspirin and placebo omega-3 PUFAs; placebo aspirin and active omega-3 PUFAs; placebo aspirin and placebo omega-3 PUFAs. Participants in the second category were randomised 1:1 to one of two parallel groups: active omega-3 PUFAs or placebo omega-3 PUFAs. Within both strata, interventions were allocated using adaptive minimisation with planned fistula site (lower vs upper arm) and study site (hospital or dialysis unit) as minimisation variables.

The overall design is illustrated in Figure 1. The key comparison is that between active omega-3 PUFAs (arms A, B and E) and placebo omega-3 PUFAs (arms C, D and F).

Figure 1. Study schema

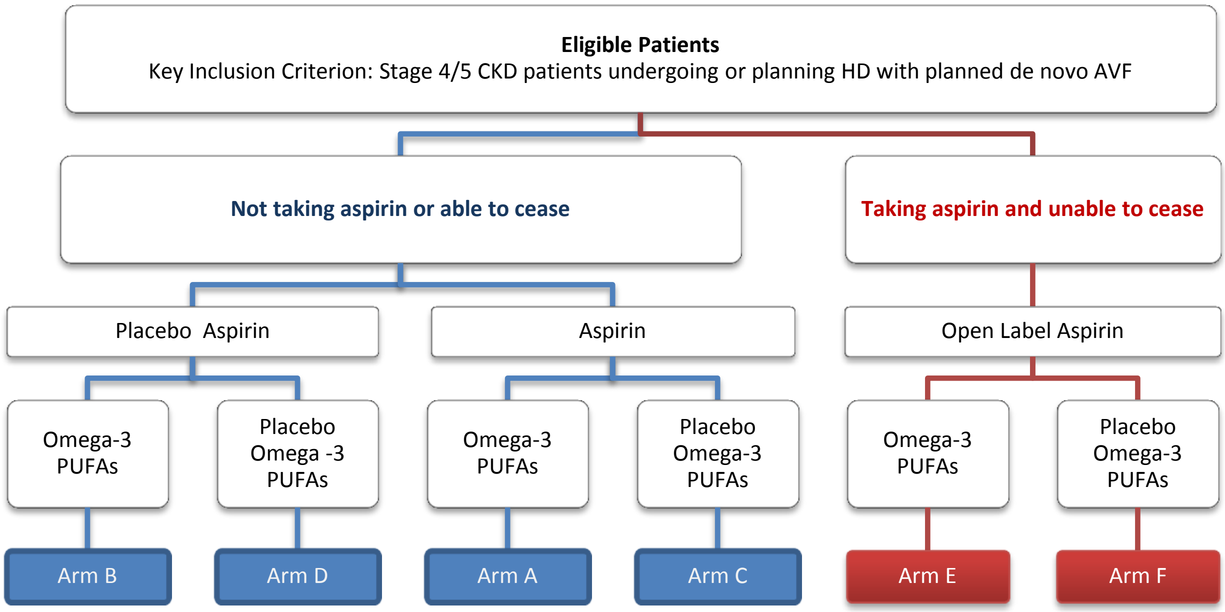

2.4. Sample size

The amended study was sized to detect a clinically relevant difference between omega-3 PUFAs and omega-3 PUFAs control groups (arms A, B and E combined versus C, D and F combined) on the primary composite outcome of AVF access failure. The 12- month post-surgery event rate for AVF access failure was assumed to be at least 30% in the control group. It was expected that the reduction in AVF access failure due to omega-3 PUFAs would be 30% (an absolute reduction in risk of 9%). To detect a 30% relative risk reduction with 80% power and a significance level of 5%, 734 participants would be required (367 per group). To allow for a 5% drop-in from control to omega-3 PUFAs and a 5% drop-out from omega-3 PUFAs to placebo, the total number of participants increased to 906 (453 per group). Allowing for a 5% loss to follow-up, a total of 954 participants needed to be

recruited. The assumed 30% event rate for AVF access failure is conservative when compared to the 60% event rate reported by the North American clopidogrel Trial (25) and other recently published studies (23, 26). However, the vascular access practices and AVF outcomes in the United States differ from those in Australasia. Moreover, the report to the FAVOURED Data and Safety Management Board in December 2011 showed a 38% blinded pooled event rate among the first 184 participants, indicating that a vascular access failure rate of 30% is a reasonable assumption.

2.5 Treatment blinding and allocation concealment

Investigators and patients were blinded to treatment assignment. Microbiology staff in local laboratories who performed outcome assessments were blinded to the patient's assigned treatment. To ensure concealment of treatment allocation, randomisation was performed using a central web-based randomisation system called Flexetrials™ administered by the National Health and Medical Research Council Clinical Trials Centre in Sydney.

2.6 Schedule of assessments

**Study Phase**	**Baseline**	**Surgery**	**Treatment Period (wks)**			**Follow-up Period (mths)**			
**Assessment**	**≤ 4wks prior to surgery**		**1**	**6**	**12**	**6**	**12**	**24**	**36**
Informed Consent	X								
Medical History	X								
Weight and blood pressure	X	X	X	X	X	X	X	X	X
Demographic characteristics	X								
Physical examination	X								
Dialysis status	X		X	X	X	X	X	X	X
Description and status of AVF		X	X	X	X	X	X	X	X
AVF Access Failure components		X	X	X	X	X	X		
Concomitant medications	X	X	X	X	X	X			
Adverse events		X	X	X	X	X			
**Fasting Pathology Tests**									
Haematology and coagulation	X				X				
Biochemistry and lipid profile	X				X				
Urinary protein/creatinine ratio	X				X				
Optional: Homocysteine and Lp(a)	X				X				
**Stored Samples for ANZ Sites** (for central laboratory analysis)									
RBC fatty acid sample, hsCRP	X			X	X				
Urinary thromboxane B2 and prostacyclin metabolites	X				X				

3. STUDY OUTCOME VARIABLES

3.1 Primary outcome

The primary outcome is AVF access failure (no, yes) within 12 months after AVF creation. AVF access failure is a composite outcome comprising three clinical components:

1. Thrombosis of the AVF: This is defined as the absence of a thrill or bruit by clinical assessment and/or requirement for rescue interventions including medical thrombolysis or surgical thrombectomy to restore patency for thrombosis or occlusion for the study AVF between AVF surgery and the month 12 visit.
2. AVF Abandonment: This is defined as the permanent abandonment of study AVF between AVF surgery and the month 12 visit. Events that may indicate AVF abandonment include thrombosis of the study AVF, imaging showing that the study AVF is unusable or not amenable to any intervention for its improvement, insertion of another dialysis access (new AVF, AVG, CVC or peritoneal dialysis access) or ligation of the study AVF due to thrombosis.
3. Cannulation Failure: This is defined as the failure to successfully cannulate the study AVF with 2 needles (or with 1 needle if using a single needle dialysis method) during 8 or more out of 12 haemodialysis sessions during the Cannulation Assessment Period (CAP). CAP is defined as the first 12 consecutive maintenance HD sessions after the week 12 visit (end of study intervention). HD sessions that occur before week 12 are not part of the CAP. A study AVF that is abandoned before the 12 month visit will be considered to have failed the CAP. Participants who do not commence HD before the 12 month visit are not assessable for cannulation failure.

The three components are being independently adjudicated by two observers, each blinded to the medical history of participants and their treatment assignment, and categorised as 0=no or 1=yes. Disagreements are being documented and will be resolved by consensus. AVF access failure will be coded 1=yes for participants with at least one component event and 0=no for participants with no component events.

3.2 Secondary outcomes

Data are being collected on a range of secondary outcome variables related to AVF access. The key secondary outcomes are the components of the primary composite outcome. The full list of secondary outcomes is as follows:

1. Thrombosis of the study AVF between AVF surgery and the month 12 visit, where thrombosis is a dichotomous outcome (no, yes) as defined for the primary composite outcome.
2. AVF abandonment between AVF surgery and the month 12 visit, where abandonment is a dichotomous outcome (no, yes) as defined for the primary composite outcome.
3. Cannulation failure between AVF surgery and the month 12 visit, where cannulation failure is a dichotomous outcome (no, yes) as defined for the primary composite outcome.
4. Primary patency (no, yes) of the AVF during the first 12 months after AVF surgery. Primary patency is defined as the presence of an audible bruit over the site of the arterio-venous anastomosis and is recorded at the following time points: within 24 hours post-surgery, and at visits 1, 6, and 12 weeks and 6 and 12 months after AVF surgery.

5. Failure (no, yes) of a patent AVF to cannulate during the cannulation assessment period.
6. Number and type of interventions the study AVF required between AVF surgery and the month 12 visit. Interventions include rescue procedures designed to restore patency of the AVF (medical thrombolysis or surgical thrombectomy) and non-rescue procedures (surgical or radiological revision or dilatation of the AVF from or proximal to the anastomosis to the ipsilateral central vein, dilation of central venous stenosis, ligation of tributaries, superficialisation of AVF or ligation of fistula or salvage by DRIL [distal reconstruction and interval ligation] due to distal ischaemia). Specific outcomes will be:
    a. Any intervention (no, yes)
    b. Total number of interventions
    c. Any rescue intervention (no, yes)
    d. Total number of rescue interventions
    e. Any non-rescue intervention (no, yes)
    f. Total number of non-rescue interventions
7. Time to first thrombosis/rescue intervention is defined as the time from AVF creation to the first occasion of a rescue intervention up to the month 12 visit.
8. Time to AVF abandonment is defined as the time from AVF surgery to permanent abandonment of study AVF up to the 12 month visit.
9. Time to the first component event of AVF thrombosis/rescue intervention or AVF abandonment.
10. Time to first successful cannulation is defined as the time from the AVF surgery to the first successful attempt at access cannulation up to the 12 month visit.
11. CVC requirement from the end of the 12-week study intervention period to the 12 month visit where CVC requirement is defined as the use of a CVC on any occasion to provide vascular access for haemodialysis (binary: no, yes).
12. Any CVC requirement during the CAP (no, yes)
13. Any CVC requirement between the end of the CAP and the 12 month visit (no, yes)
14. Number of days a CVC was in situ between the week 12 and 12 month visit

3.3 Tertiary outcomes

The following have been designated as tertiary outcome variables:

1. Any CVC requirement up to the 12 month visit (no, yes)
2. Long term outcomes of AVF:
    a. AVF abandonment by the 24 month visit (no, yes)
    b. AVF abandonment by the 36 month visit (no, yes)
    c. Time to AVF abandonment (administratively censored at 36 months)
3. Accuracy (unsure/incorrect, correct) of participant’s best guess about whether they received active or placebo omega-3 PUFA treatment - measured at the end of the treatment period
4. Accuracy (unsure/incorrect, correct) of physician’s best guess about whether their patient had received active or placebo omega-3 PUFA treatment - measured at the end of the treatment period

3.4 Safety outcomes

The following safety outcome variables have been recorded:

1. Any SAE
    a. Death due to any cause
    b. Any life-threatening event
    c. Any initial or prolonged inpatient hospitalisation
    d. Any persistent or significant disability/incapacity
    e. Any important medical event
    f. Any congenital abnormality/birth defect
2. SAE body system (10 categories)
3. SAE relationship to study medication (none, unlikely, possible, probable)
4. Any adverse drug reaction (ADR)
    a. Any bleeding event
    b. Any gastrointestinal event
    c. Other event
5. ADR severity (mild, moderate, severe, life-threatening)
6. ADR relationship to study medication (possible, probable)

4. SEQUENCE OF PLANNED ANALYSES

4.1 Interim analyses

An independent Data and Safety Monitoring Board (DSMB) comprising experts in clinical trials, biostatistics, and nephrology was in charge of reviewing un-blinded data on patient characteristics, treatment compliance, and safety and efficacy outcomes. Two formal interim efficacy analyses were planned after 1/3 and 2/3 of total patients had been recruited and followed for at least 12 months. The Haybittle-Peto rule was used as a stopping guideline for efficacy (27). Due to cessation of recruitment well before the target sample size, only the first efficacy interim analysis was performed. The DSMB met on 20 March 2013 to consider the results of this analysis and recommended continuation of the trial. SAEs, including bleeding events, have been monitored on a regular basis by the DSMB. Only DSMB members and statisticians compiling the closed-session reports for DSMB meetings had access to un-blinded interim data and results before database lock.

4.2 Blinded reviews of primary outcome

Blinded reviews of the primary outcome data were performed to check that the assumptions underlying the original sample size calculations remained plausible. These reviews were conducted at irregular intervals by clinicians and a statistician blinded to treatment allocation.

4.3 Planned intervention group comparisons

4.3.1 Primary outcome

The following intervention group comparisons are planned for the primary outcome:

1. Omega-3 PUFAs (Arms A, B & E) versus placebo omega-3 PUFAs (Arms C, D & F)
2. Omega-3 PUFAs versus placebo omega-3 PUFAs under open label aspirin (Arm E vs F)
3. Aspirin (Arms A & C) versus placebo aspirin (Arms B & D)
4. Combined aspirin and omega-3 PUFAs (Arm A) versus combined placebo aspirin and placebo omega-3 PUFAs (Arm D)
5. Subgroups formed by:
   a. Planned AVF site (lower arm, upper arm)
   b. Actual AVF site (lower arm, upper arm)
   c. Diabetes (no, yes)
   d. Gender (female, male)
   e. Age (quartiles)
   f. Any cardiovascular disease (no, yes [peripheral vascular disease, cerebrovascular disease, ischaemic heart disease])
   g. Dialysis at baseline (no [pre-dialysis, transplant], yes [haemodialysis, peritoneal dialysis])

4.3.2 Secondary outcomes

The following intervention group comparisons are planned for fourteen secondary outcomes:

1. Omega-3 PUFAs (Arms A, B & E) versus placebo omega-3 PUFAs (Arms C, D & F)
2. Omega-3 PUFAs versus placebo omega-3 PUFAs under open label aspirin (Arm E vs F)
3. Aspirin (Arms A & C) versus placebo aspirin (Arms B & D)

4.3.3 Tertiary outcomes

A single comparison is planned for each tertiary outcome variable: omega-3 PUFAs (Arms A, B, & E) versus placebo omega-3 PUFAs (Arms C, D, & F).

4.3.4 Safety outcomes

The following intervention group comparisons are planned for all safety outcomes:

1. Omega-3 PUFAs (Arms A, B, & E) versus placebo omega-3 PUFAs (Arms C, D, & F)
2. Omega-3 PUFAs versus placebo omega-3 PUFAs under open label aspirin (Arm E vs F)
3. Aspirin (Arms A & C) versus placebo aspirin (Arms B & D)

In addition, ADRs that are bleeding events will be summarised in more detail by comparing Arms A versus B versus C versus D and Arms A+E versus B versus C+F versus D.

4.4 Final analyses and reporting

All final planned analyses identified in the protocol and in this SAP will be performed only after the last patient has completed the 12 month post-surgery follow-up assessment and the database has been cleaned and locked. A blinded data review meeting will be held by the

Trial Management Committee (TMC) prior to database lock. The database will not be locked, randomisation un-blinded or analysis commenced until this SAP has been approved by the TMC. Key statistics and trial results from the final analyses will be presented to the TMC for discussion prior to completion of the FSR and subsequent manuscripts. Any post-hoc exploratory analyses performed to provide support for planned analyses but not identified in this SAP will be documented and reported in appendices to the FSR and clearly identified as unplanned analyses in the text of the FSR.

5. CHANGES TO STATISTICAL INFORMATION IN THE PROTOCOL

The following amendments have been made to statistical information given in the original publication of the study protocol (22):

1. The primary outcome was changed from unassisted patency of the AVF at three months after randomisation to a composite measure of AVF access failure up to 12 months after AVF surgery. This amendment was in response to external results reported by Dember et al. (25).
2. The original 2*2 factorial design was expanded to 6 groups to increase the recruitment rate by allowing recruitment of patients who were taking aspirin and unable to cease. These aspirin-taking patients were randomised to receive active omega-3 PUFAs or placebo omega-3 PUFAs. As a result of this amendment, omega-3 PUFAs became the primary focus of the trial and aspirin was downgraded to a secondary objective.
3. Following from the first two changes, the sample size was recalculated to accommodate the new primary outcome.
4. Additional subgroups have been pre-specified. The original protocol reported one subgroup (AVF site). The TMC revised the number of subgroups at a meeting held in December 2014 specifically to discuss details of the SAP.

With the exception of the change to subgroups, the above changes to the study design and methods were incorporated into a revised study protocol in November 2010 (7) and subsequently published (1).

6. ANALYSIS PRINCIPLES

6.1 Intention-to-treat principle and analysis dataset

Tests of the effects of treatment on the primary composite outcome and the key secondary outcomes (i.e., the three components of the composite) will be conducted as close as possible to the intention-to-treat (ITT) ideal. All randomised patients who undergo their scheduled AVF surgery will be included in the "full analysis set" and analysed in the group to which they were randomly allocated regardless of whether the y received the assigned treatment and irrespective of any protocol deviations or violations. Analyses of these and other efficacy outcome variables will exclude the following randomised patients: (1) those who did not have an AVF created, either because their scheduled AVF surgery was cancelled, AVG surgery was performed instead, or an AVF was attempted but did not successfully form; (2) those who died within 12 months of surgery and were not assessed on any of the three components of the primary outcome before their death; and (3) those who withdrew from study treatment

and withdrew consent for use of their data. These discontinuations will be captured in a figure illustrating participant progression through the trial.

Missing primary outcome data will be imputed using a method based on assumptions that are clinically defensible and unlikely to be biased in favour of the omega-3 PUFAs intervention. Robustness of results to the assumptions underlying the imputation method will be evaluated using a global sensitivity analysis approach which involves analyses based on different assumptions that trend towards best and worst case scenarios (28). Imputation and sensitivity analysis methods for the primary outcome are described in section 7.1.

6.2 Multicentre study and heterogeneity

Thirty-five dialysis centres recruited between one and 50 participants. Although included as a variable in the randomisation scheme, study centre will not be adjusted for in the main analyses of primary and key secondary efficacy outcomes because some centres were small (≤ 5 participants) and may not contribute any events to the pooled data. However, if there is a positive treatment effect on the primary composite outcome and/or the component events, study centres will be combined within five Australian states, within Malaysia, and within a pooled New Zealand/United Kingdom (seven regional strata in total) to assess the homogeneity of the treatment effect across regions. Potential heterogeneity will be tested by including a treatment-by-region interaction term in a multivariable model with treatment and region as main effects.

6.3 Multiple comparisons and multiplicity

The Heybittle-Peto rule with a maximum of three analyses (two interims and one final) was used to control the overall Type 1 error rate for analysis of the effect of omega-3 PUFAs on the composite primary outcome variable. The trial continued after the first of two planned interim analyses. The second interim analysis was not performed due to early cessation of recruitment and so the final analysis will be assessed against an alpha of 0.05. There will be no further adjustments for multiplicity as there is a pre-defined hierarchy of importance of outcome variables and study objectives and the influence of individual results on the overall interpretation of the trial will reflect their level within this hierarchy. Further, there are a limited number of subgroups, all of which are pre-specified. All statistical tests of significance will be two-sided.

6.4 Covariate adjustment of main analyses

The main statistical analyses of primary and secondary efficacy outcomes comparing active omega-3 PUFAs (Arms A, B & E) with placebo omega-3 PUFAs (Arms C, D & F) will be adjusted for aspirin strata (taking aspirin at randomisation, randomised to aspirin, randomised to placebo aspirin). Supporting analyses will be additionally adjusted for regional location of the clinical site and six pre-specified prognostic baseline characteristics (section 7.1.3). Comparisons of other groups on the primary and secondary efficacy outcomes will be unadjusted, with supporting analyses adjusted as described for the main omega-3 PUFA comparison.

7. STATISTICAL METHODS

7.1 Analysis of the primary outcome

The primary outcome is AVF access failure within 12 months of AVF creation due to one or more of AVF thrombosis/rescue intervention, AVF abandonment, and AVF cannulation failure. Each component will be coded as no event = 0 and an event = 1. The composite will be coded as no component events = 0 and at least one component event = 1. There are two broad reasons why data may be missing on one or more AVF access failure components: the participant withdrew from the study within 12 months of AVF surgery by choice, due to receipt of a kidney transplant, or was lost to follow-up and was not assessed on all three components of the primary outcome before withdrawal; the participant completed 12 months of follow-up but did not commence HD and was not assessed for cannulation during the 12 month follow-up period. Participants with missing data on a component will be randomly allocated a zero or one according to the proportion of events in participants with valid data for the component. This method is consistent with an assumption that data are missing completely at random (MCAR) and justifiable on the grounds that the main reasons for withdrawal are unlikely to be related to the study treatment or the status of the patient's fistula.

7.1.1 Primary comparison

Log binomial regression will be used to compare omega-3 PUFAs (Arms A, B & E) to placebo PUFAs (Arms C, D & F) on AVF access failure. The analysis will be adjusted for aspirin-taking strata (randomised to active aspirin, randomised to placebo aspirin, open-label aspirin). In the log binomial regression model, the omega-3 PUFA versus placebo PUFA groups will be represented as a binary indicator variable and aspirin taking strata (3 groups) will be represented as two binary indicator variables with aspirin placebo as the reference group. The main result will be the treatment effect risk ratio and 95% confidence interval adjusted for aspirin strata.

7.1.2 Supporting and sensitivity analyses

Robustness of the estimate of the omega-3 PUFA treatment effect will be assessed by an analysis which additionally adjusts for baseline characteristics either known or *a priori* suspected to be associated with the primary outcome (29). The relevant baseline characteristics are: planned AVF site (lower arm, upper arm), diabetes (no, yes), gender (female, male), age (years), any cardiovascular disease (no, yes [peripheral vascular disease, cerebrovascular disease, ischaemic heart disease]), and dialysis at baseline (no [pre-dialysis, transplant], yes [haemodialysis, peritoneal dialysis]). This covariate adjusted model will include main effects for treatment group and the six covariates but will not include interaction terms. An adjusted analysis will be viewed as supportive, providing additional context for interpreting the primary unadjusted analysis. If there is a statistically significant effect of omega-3 PUFAs on AVF access failure, heterogeneity of the effect will be tested by including a treatment by region (Australian state, Malaysia, New Zealand/United Kingdom) interaction effect in the primary and robustness models.

Sensitivity of the result of the primary treatment comparison to single imputation of a trial-specific representative proportion of events for each component will be assessed as follows:

1. Repeat the log binomial regression analysis on best- and worst-case single imputation scenarios. In the former, all missing values on component events in the omega-3 group will be assumed to be non-events and all those in the placebo omega-3 group will be assumed to be events. The worst-case scenario will be based on the reverse pair of assumptions. In addition, a range of intermediate scenarios will be assessed.
2. Repeat the log binomial regression analysis on complete cases. This analysis will be performed on the composite outcome but include data only from participants with observed data on all three components.
3. Perform a maximum likelihood-based analysis using observations on the three components. Participants with no observed data will not contribute to the maximum likelihood estimate of component and composite relative risks.
4. Analyse the composite outcome using a pattern mixture model allowing for two dominant patterns of missing component (and therefore composite) data: participants who completed 12 months of follow-up but had incomplete data because their AVF was not assessed for cannulation and they did not have an event on the other two components; and participants who withdrew before the 12 month visit.

These sensitivity analyses are variously designed to address potential bias in the main estimate of treatment effect and the main problem with single imputation, namely that the method does not take account of uncertainty pertaining to missing data with the result that the estimate of variability in the treatment effect may be biased downwards and inflate Type I error above the nominal 5% level.

7.1.3 Subgroup analyses

Pre-specified subgroups are formed by the following baseline characteristics: planned AVF site (lower arm, upper arm), actual AVF site (lower arm, upper arm), diabetes (no, yes), gender (female, male), age (quartiles), any cardiovascular disease (no, yes [peripheral vascular disease, cerebrovascular disease, ischaemic heart disease]), and dialysis at baseline (no [pre-dialysis, transplant], yes [haemodialysis, peritoneal dialysis]). The main subgroup analyses will be tests of treatment-by-subgroup variable interactions in log binomial regression models adjusted for aspirin strata. These analyses will be performed regardless of the result of the log binomial model for the primary comparison. Risk ratio estimates of treatment effect (omega-3 PUFAs versus placebo PUFAs) within each sub-group will be reported along with their 95% confidence limits.

7.1.4 Other comparisons

Log binomial regression models will be used to compare omega-3 PUFAs and placebo PUFAs under open label aspirin (Arm E versus F), aspirin and placebo aspirin (Arms A & C versus B & D), and combined aspirin and omega-3 PUFAs (Arm A) relative to placebo aspirin and placebo PUFAs (Arm D). These models will have a single binary indicator variable representing the treatment group comparison and will not be adjusted for other characteristics. The second model assumes an additive effect for aspirin and omega-3 PUFAs which will be assessed by initially including a treatment (aspirin or placebo aspirin) by treatment (PUFAs or placebo PUFAs) interaction term in the log binomial model. Supporting and sensitivity analyses performed for the primary comparison will be performed for these secondary comparisons.

7.2 Analysis of secondary outcomes

Secondary outcome variables are variously binary, count, and time-to-event data. Binary outcomes with a single measurement per participant such as the three components of the primary outcome will be analysed using a log binomial regression model to obtain estimates of risk ratios. Primary patency is a binary outcome which is assessed repeatedly within participants. To accommodate the within participant correlation, this outcome will be analysed using a generalised estimating equation approach for relative risks with an unstructured variance-covariance matrix (Toeplitz if convergence fails). Count outcomes will be analysed with Poisson regression models. If there is under- or over-dispersion, residuals will be examined to determine the most suitable model (e.g., quasi-Poisson regression, negative binomial regression). Time-to-event outcomes will be analysed with a competing risks regression model. Competing risks for time to first rescue intervention and time to first successful cannulation will be AVF abandonment, death and renal transplant. Competing risks for time to AVF abandonment will be death and renal transplant. As there will be relatively few competing risk events, these analyses will be performed with a composite competing risk group. Participants who did not experience the relevant outcome event or a competing risk event by 12 months after surgery will be censored at this time and those who withdrew from the study will be censored at the time of withdrawal.

Omega-3 PUFAs (Arms A, B & E) will be compared to placebo PUFAs (Arms C, D & F) on all secondary outcomes using a statistical model appropriate for the outcome. The same statistical models will be used to compare omega-3 PUFAs versus placebo PUFAs under open label aspirin (Arm E versus F) and aspirin (Arms A & C) versus placebo aspirin (Arms B & D) for participants who were not taking aspirin or able to cease before randomisation.

7.3 Analysis of tertiary outcomes

Tertiary outcome variables are binary and time-to-event data. Omega-3 PUFAs (Arms A, B & E) will be compared to placebo PUFAs (Arms C, D & F) on all tertiary outcomes using a statistical model appropriate for the type of outcome as described for secondary outcomes.

7.4 Analysis of safety outcomes

All treatment groups will be compared on safety outcomes: omega-3 PUFAs (Arms A, B & E) versus placebo PUFAs (Arms C, D & F); omega-3 PUFAs versus placebo PUFAs under open label aspirin (Arm E versus F); and aspirin (Arms A & C) versus placebo aspirin (Arms B & D) for participants who were not taking aspirin or able to cease before randomisation. The relationship of each category of SAE and ADR (present versus absent) to treatment group will be summarised by frequencies and percentages and analysed with chi-square tests. The relationship between treatment group and SAE body system (10 categories), SAE relationship to study medication (none, unlikely, possible, probable), type of ADR event (bleeding, gastrointestinal, other), ADR severity (mild, moderate, severe, life-threatening), and ADR relationship to study medication (possible, probable) will be summarised by frequencies and percentages and analysed with chi-square tests. The same methods will be used to summarise in more detail ADRs that are bleeding events. The more detailed summaries will compare Arms A versus B versus C versus D and Arms A+E versus B versus C+F versus D.

7.5 Data manipulation and computing

All trial data collected after randomisation are stored in a central InForm® database. Most data manipulation, tables, figures, listings and analyses will be documented in SAS® programs and performed using SAS version 9.4. Other data preparation and analyses will be documented in Stata® programs and performed using Stata version 13.1.

8. REPORTING

All results described above as well as tables, listings and figures (TLFs) listed below will be presented in the FSR.

8.1 Trial profile

All patients who provide informed consent will be accounted for in the FSR. A CONSORT-style flow diagram will illustrate patient progression through the trial from initial screening for eligibility to completion of the final primary outcome assessment. Number (percentage) of participants randomised to each treatment group will be given for all randomised patients along with reasons for study withdrawal by treatment group.

8.2 Patient characteristics and baseline comparisons

Demographic and other baseline characteristics will be summarised by assigned treatment group. Categorical variables will be summarised by frequencies and percentages. Percentages will be calculated according to the number of patients for whom data are available. Where values are missing, the denominator, which will be less than the number of patients assigned to the treatment group, will be reported either in the body or a footnote in the summary table. Continuous variables will be summarised by mean and standard deviation as well as quartiles.

Baseline demographic and clinical characteristics and laboratory investigations will be presented in three tables (see Section 6.3 for further details). Each table will include the following baseline demographic and clinical characteristics: gender, age at randomisation, ethnicity (Aboriginal/Torres Strait Islander [ATSI], Asian, Caucasoid, Maori/Pacific Islander [MPI], Other, Unknown), planned AVF location (upper arm, lower arm), height, weight, Body Mass Index (BMI), waist measurement (cm), hip measurement (cm), waist/hip ratio, smoking status (never, former, current), systolic blood pressure (mm Hg), diastolic blood pressure (mm Hg), heart rate, diabetes (no, yes), type of diabetes (1, 2), primary cause of end-stage renal failure (diabetic neuropathy, glomerulonephritis, hypertension/vascular, polycystic kidney disease, reflux nephropathy, other, unknown), ischaemic heart disease (no, yes), congestive heart disease (no, yes), hypertension (no, yes), cerebrovascular disease (no, yes), peripheral vascular disease (no, yes), currently on renal replacement therapy (no, yes), type of current renal replacement therapy (transplant, automated peritoneal dialysis, continuous ambulatory peritoneal dialysis, haemodialysis), principal access currently in use for dialysis (AVF, AVG, CVC, PD catheter).

Each table will include the following baseline blood and biochemistry investigations: haemoglobin (g/L), platelets (10^9/L), white blood cells (10^9/L), International Normalised Ratio ([INR]), Activated Partial Thromboplastin Time ([APTT] sec), fibrinogen (mg/dL),

sodium (mmol/L), potassium (mmol/L), bicarbonate (mmol/L), urea (mmol/L), creatinine (µmol/L), albumin (g/dL), calcium (mmol/L), phosphate (mmol/L), fasting glucose (mmol/L [separately for diabetics and non-diabetics]), HbA1c (% [separately for diabetics and non-diabetics]), parathormone (pmol/L), triglyceride (mmol/L), cholesterol (mmol/L), HDL cholesterol (mmol/L), LDL cholesterol (mmol/L), and urine protein:creatinine ratio (mg/mmol).

8.3 TABLES, LISTINGS, AND FIGURES (TLFs)

Templates for all planned TLFs are in a separate document titled FAVOURED SAP Tables, Listings and Figures. Where a TLF gives results by treatment group, unless otherwise stated, there should be three versions of the TLF:

a) All randomised participants: active omega-3 PUFAs (arms A, B, E) compared with placebo omega-3 PUFAs (arms C, D, F)
b) Participants randomised within the open-label aspirin stratum: active omega-3 PUFAs (arm E) compared with placebo omega-3 PUFAs (arm F)
c) Participants randomised within the non-aspirin-taking stratum: active aspirin (arms A and C) compared with placebo aspirin (arms B and D).

8.3.1 Planned tables

The following are planned summary tables:

Table 1. Enrolment by study centre
Table 2. Treatment group by study centre (parts a to c)
Table 3. Aspirin stratification at randomisation (open-label versus randomised) by study centre
Table 4. Protocol deviations and violations by treatment group (parts a to c)
Table 5. Protocol deviations and violations by aspirin stratification at randomisation (open-label versus randomised)
Table 6. Medication compliance and withdrawals by treatment group (parts a to c)
Table 7. Medication compliance and withdrawals by aspirin stratification at randomisation (open-label versus randomised)
Table 8. Demographic and clinical baseline characteristics by treatment group (parts a to c)
Table 9. Demographic and clinical baseline characteristics by aspirin stratification at randomisation (open-label versus randomised)
Table 10. Baseline blood investigations by treatment group (parts a to c)
Table 11. Baseline blood investigations by aspirin stratification at randomisation (open-label versus randomised)
Table 12. Clinical assessments by treatment group across study visits (parts a to c)
Table 13. Primary outcome by treatment group (parts a to c)
Table 14. Primary outcome for subgroups by omega-3 PUFA treatment group (arms A, B, & E versus C, D, & F)
Table 15. Secondary outcomes by treatment group (parts a to c): components of the primary composite outcome
Table 16. Secondary outcomes by treatment group (parts a to c): primary patency
Table 17. Secondary outcomes by treatment group (parts a to c): number and type of AVF interventions
Table 18. Secondary outcomes by treatment group (parts a to c): CVC requirement

Table 19. Long term outcomes of AVF by omega-3 PUFA group (active vs placebo)
Table 20. Participant and physician best guess about participant's omega-3 PUFA group by participant's actual omega-3 PUFA group
Table 21. Any ADR and ADR categories by treatment group (parts a to c)
Table 22. ADR severity by treatment group (parts a to c)
Table 23. ADR relationship to study medication by treatment group (parts a to c)
Table 24. Any SAE and SAE categories by treatment group (parts a to c)
Table 25. Relationship of SAE to study medication by treatment group (parts a to c)
Table 26. SAE body system by treatment group (parts a to c)

8.3.2 Planned listings

The following are planned data and patient listings:

Listing 1. Reasons randomised participants did not have surgery
Listing 2. Reasons randomised participants had AVG rather than AVF surgery
Listing 3. Reasons for participants withdrawing from the study
Listing 4. Medication compliance problems requiring participant to come off trial
Listing 5. Deaths and life threatening events

8.3.3 Planned figures

The following are planned summary figures:

Figure 1. Monthly and cumulative entry of participants into the study
Figure 2. Flowchart of patient progression through the study
Figure 3. Forest plot of odds ratios and 95% confidence intervals for subgroup analyses (primary composite outcome and components of the composite)
Figure 4. Cumulative incidence functions for time to first thrombosis/rescue intervention by treatment groups (parts a to c)
Figure 5. Cumulative incidence functions for time to AVF abandonment by treatment groups (parts a to c)
Figure 6. Cumulative incidence functions for time to first thrombosis/rescue intervention or AVF abandonment by treatment groups (parts a to c)
Figure 7. Cumulative incidence functions for time to first successful cannulation by treatment groups (parts a to c)

8.3.4 Supplementary TLFs

Results from supporting and sensitivity analyses will be presented in supplementary tables. Missing data on the primary outcome will be summarised by treatment groups and presented in supplementary tables and figures as appropriate.

8.4 General reporting conventions

All TLFs will be presented in portrait orientation, unless landscape orientation suggests that the information is easier to view. Legends will be used for all figures with more than one variable or item displayed. Figure lines should be wide enough to see the line after being copied.

All titles will be centred on a page. The first title line will be the number of the table, figure, or data listing. The second (and if required, third) line will be the description of the table, figure, or data listing. The ICH numbering convention will be used for all TLFs (30).

All tables, figures, and data listings will have the name of the relevant SAS (or Stata) program and a date-time stamp on the bottom of each output. All analysis programs developed for a table, figure, or data listing will be self-contained to facilitate transfer of programs to multiple computing environments. A separate analysis program will be written to produce each table.

8.5 Statistical summary conventions

For tables, sample sizes for each treatment group will be presented as totals in the column header (N=xxx), where appropriate. Sample sizes shown with summary statistics are the number (n) of patients with non-missing values.

Summaries for categorical variables will include only categories that patients had a response in. Percentages corresponding to null categories (cells) will be suppressed. All summaries for continuous variables will include: N, mean, and SD. Other summaries (e.g. median, quartiles, 5%, 95% intervals, coefficient of variation (CV) or %CV will be used as appropriate. All percentages should be rounded and reported to a single decimal place (xx.x%). If percentages are reported as integers, percentages greater than 0% but <1% will be reported as <1%, whereas percentages greater than 99% but <100% will be reported as >99%. A percentage of 100% will be reported as 100%. No value of 0% should be reported. Any computation of percent that results in 0% is to be reported as a blank. Summaries that include p-values will report the p-value to three decimal places with a leading zero (0.001). P-values <0.001 will be reported as <0.001.

9. REFERENCES

1. Viecelli AK, Pascoe E, Polkinghorne KR, Hawley C, Paul-Brent PA, Badve SV, et al. The Omega-3 fatty acids (Fish Oils) and Aspirin in Vascular access OUtcomes in Renal Disease (FAVOURED) study: the updated final trial protocol and rationale of post-initiation trial protocol modifications. BMC Nephrology. 2015(16).
2. ICH. ICH harmonised tripartite guideline: statistical principles for clinical trials E9. 1998.
3. ASA. Ethical guidelines for statistical practice. Prepared by the Committee on Professional Ethics.1999. Available from: http://www.amstat.org/about/ethicalguidelines.cfm.
4. NZSA. Code of Conduct of the New Zealand Statistical Association. 1995. Available from: http://stats.org.nz/code_of_conduct.shtml.
5. RSS. The Royal Statistical Society: Code of Conduct.1993. Available from: http://www.rss.org.uk/uploadedfiles/documentlibrary/142.pdf.
6. SSAI. The Statistical Society of Australia Inc. Code of Conduct.
7. The FAVOURED Trial Protocol, Version 9, 30 November 2009.
8. The FAVOURED Trial Charter of the Data and Safety Monitoring Board (DSMB), 13 June 2011.
9. ICH. ICH Harmonised tripartite guideline: guideline for good clinical practice E6. 1996.
10. Feldman HI, Held PJ, Hutchinson JT, Stoiber E, Hartigan MF, Berlin JA. Hemodialysis vascular access morbidity in the United States. Kidney International. 1993;43(5):1091-6.

11. Feldman HI, Kobrin S, Wasserstein A. Hemodialysis vascular access morbidity. Journal of the American Society of Nephrology. 1996;7(4):523-35.
12. Manns B, Tonelli M, Yilmaz S, Lee H, Laupland K, Klarenbach S, et al. Establishment and maintenance of vascular access in incident hemodialysis patients: a prospective cost analysis. Journal of the American Society of Nephrology. 2005;16(1):201-9.
13. Allon M, Robbin ML. Increasing arteriovenous fistulas in hemodialysis patients: problems and solutions. Kidney International. 2002;62(4):1109-24.
14. Pastan S, Soucie JM, McClellan WM. Vascular access and increased risk of death among hemodialysis patients. Kidney International. 2002;62(2):620-6.
15. Polkinghorne KR, McDonald SP, Atkins RC, Kerr PG. Vascular access and all-cause mortality: a propensity score analysis. Journal of the American Society of Nephrology. 2004;15(2):477-86.
16. Andrassy K, Malluche H, Bornefeld H, Comberg M, Ritz E, Jesdinsky H, et al. Prevention of p.o. clotting of av. cimino fistulae with acetylsalicyl acid. Results of a prospective double blind study. Klinische Wochenschrift. 1974;52(7):348-9.
17. Kaufman JS. Antithrombotic agents and the prevention of access thrombosis. Seminars in Dialysis. 2000;13(1):40-6.
18. Engstrom K, Wallin R, T. S. Effect of low-dose aspirin in combination with stable fish oil on whole blood production of eicosanoids. Prostaglandins, leukotrienes, and essential fatty acids. 2001;64(6):291-7.
19. Fiskerstrand CE, Thompson IW, Burnet ME, Williams P, Anderton JL. Double-blind randomized trial of the effect of ticlopidine in arteriovenous fistulas for hemodialysis. Artificial organs. 1985;9(1):61-3.
20. Grontoft KC, Larsson R, Mulec H, Weiss LG, Dickinson JP. Effects of ticlopidine in AV-fistula surgery in uremia. Fistula Study Group. Scandinavian journal of urology and nephrology. 1998;32(4):276-83.
21. Grontoft KC, Mulec H, Gutierrez A, Olander R. Thromboprophylactic effect of ticlopidine in arteriovenous fistulas for haemodialysis. Scandinavian Journal of urology and nephrology 1985;19(1):55-7.
22. Irish A, Dogra G, Mori T, Beller E, Heritier S, Hawley C, et al. Preventing AVF thrombosis: the rationale and design of the Omega-3 fatty acids (Fish Oils) and Aspirin in Vascular access OUtcomes in REnal Disease (FAVOURED) study. BMC Nephrology. 2009;10:1.
23. Schinstock CA, Albright RC, Williams AW, Dillon JJ, Bergstralh EJ, Jenson BM, et al. Outcomes of arteriovenous fistula creation after the Fistula First Initiative. Clinical Journal of the American Society of Nephrology. 2011;6(8):1996-2002.
24. Lok CE, Moist L, Hemmelgarn BR, Tonelli M, Vazquez MA, Dorval M, et al. Effect of fish oil supplementation on graft patency and cardiovascular events among patients with new synthetic arteriovenous hemodialysis grafts: a randomized controlled trial. JAMA. 2012;307(17):1809-16.
25. Dember LM, Beck GJ, Allon M, Delmez JA, Dixon BS, Greenberg A, et al. Effect of clopidogrel on early failure of arteriovenous fistulas for hemodialysis: a randomized controlled trial. Journal of the American Medical Association. 2008;299(18):2164-71.
26. Biuckians A, Scott EC, Meier GH, Panneton JM, Glickman MH. The natural history of autologous fistulas as first-time dialysis access in the KDOQI era. Journal of vascular surgery. 2008;47(2):415-21; Discussion 20-1.
27. Jennison C, Turnbull BW. Group sequential methods with applications to clinical trials. Washington, D.C.: Chapman & GHall/CRC; 2000.
28. (U.S.). NRC. The prevention and treatment of missing data in clinical trials. Washington, DC.: National Academies Press.; 2010.

29. Pocock SJ, Assmann SE, Enos LE, Kasten LE. Subgroup analysis, covariate adjustment and baseline comparisons in clinical trial reporting: current practice and problems. Statist Med. 2002;21:2917-30.
30. ICH. ICH Harmonised Tripartite Guideline: Structure and Content of Clinical Study Reports E3. 1995.
