# Supplementary material for: Fish oil and aspirin effects on arteriovenous fistula function: Secondary outcomes of the randomised omega-3 fatty acids (Fish oils) and Aspirin in Vascular access OUtcomes in REnal Disease (FAVOURED) trial
Source: PLoS One. 2019 Mar 26;14(3):e0213274. doi: 10.1371/journal.pone.0213274 (PMC6435148; doi:10.1371/journal.pone.0213274)
Supplement: S1 Table — (DOC) [file pone.0213274.s004.doc]

**S1 Table. Outcomes including measurement definition, metrics and method of aggregation**

| **Outcome** | **Definition** | **Metric(s)/Method of aggregation** |
| --- | --- | --- |
| **Interventions** | Medical, surgical or radiological interventions performed on the study AVF within the first 12 months of creation including: | Proportion of participants with at least 1 intervention  Rate of interventions (number of interventions per 1000 patient-days)  Time to first intervention |
| - **Rescue interventions** | Medical thrombolysis or surgical thrombectomy |
| - **Non-rescue interventions** | Surgical or radiological revision or dilation of the AVF from or proximal to the anastomosis to the ipsilateral central vein, dilation of central venous stenosis, ligation of tributaries, superficialisation of AVF, ligation of AVF or salvage by distal reconstruction and interval ligation due to distal ischemia [DRIL] |
| **First successful cannulation** | First of three consecutive successful cannulations | Proportion of participants with successful cannulation  Time to event |
| **Primary patency loss** | First thrombosis or need for rescue intervention (i.e. medical thrombolysis or surgical thrombectomy) | Proportion of participants with primary patency loss  Time to event |
| **Permanent AVF abandonment** | No further use of, or attempt to intervene on, the study AVF | Time to event |
| **CVC exposure** | CVC requirement  CVC in situ | Proportion of participants requiring at least 1 CVC  Median number of days in situ |
| **Late dialysis suitability failure** | Inability to cannulate the study AVF for at least 8 out of 12 consecutive HD sessions or permanent access abandonment within the first 6 months post-surgery | Proportion of participants with late dialysis suitability failure |

Abbreviations: AVF – arteriovenous fistula; CVC – central venous catheter
