# Supplementary material for: Fish oil and aspirin effects on arteriovenous fistula function: Secondary outcomes of the randomised omega-3 fatty acids (Fish oils) and Aspirin in Vascular access OUtcomes in REnal Disease (FAVOURED) trial
Source: PLoS One. 2019 Mar 26;14(3):e0213274. doi: 10.1371/journal.pone.0213274 (PMC6435148; doi:10.1371/journal.pone.0213274)
Supplement: S2 Table — Type and frequency of AVF interventions for fish oil versus placebo (A) and aspirin versus placebo (B). (DOCX) [file pone.0213274.s005.docx]

**S2 Table. Type and frequency of AVF interventions for fish oil versus placebo (A) and aspirin versus placebo (B)**

| **Table 2A \| AVF interventions for fish oil versus placebo** | | | | |
| --- | --- | --- | --- | --- |
| **Outcomes** | **Fish Oil (n=270)** | **Placebo**  **(n=266)** | **Relative risk**  **(95% CI)^d^** | ***P* Value** |
| Patients with ≥ 1 intervention, n (%)^a^   - 1 intervention - 2 interventions - 3 interventions - 4 interventions | 60 (22)  50 (19)  8 (3)  2 (1)  0 | 72 (27)  52 (20)  14 (5)  3 (1)  3 (1) | 0.82 (0.61, 1.11) | 0.20 |
| Patients with ≥ 1 rescue intervention, n (%)^b^   - 1 intervention - 2 interventions | 8 (3)  8 (3)  0 | 13 (5)  11 (4)  2 (1) | 0.60 (0.25, 1.42) | 0.24 |
| Patients with ≥ 1 non-rescue intervention, n (%)^c^   - 1 intervention - 2 interventions - 3 interventions - 4 interventions | 55 (20)  48 (18)  5 (2)  2 (1)  0 | 66 (25)  52 (20)  9 (3)  4 (2)  1 (<1) | 0.82 (0.60, 1.12) | 0.22 |
| **Table 2B \| AVF interventions for aspirin versus placebo** | | | | |
| **Outcomes** | **Aspirin (n=194)** | **Placebo**  **(n=194)** | **Relative risk**  **(95% CI)** | ***P* Value** |
| Patients with ≥ 1 intervention, n (%)^a^   - 1 intervention - 2 interventions - 3 interventions - 4 interventions | 44 (23)  34 (18)  7 (4)  2 (1)  1 (1) | 49 (25)  36 (19)  9 (5)  2 (1)  2 (1) | 0.9 (0.63, 1.28) | 0.55 |
| Patients with ≥ 1 rescue intervention, n (%)^b^   - 1 intervention - 2 interventions | 5 (3)  4 (2)  1 (1) | 12 (6)  11 (6)  2 (1) | 0.42 (0.15, 1.16) | 0.58 |
| Patients with ≥ 1 non-rescue intervention n (%)^c^   - 1 intervention - 2 interventions - 3 interventions - 4 interventions | 42 (22)  34 (18)  6 (3)  2 (1)  0 | 43 (22)  36 (19)  3 (2)  3 (2)  1 (1) | 0.98 (0.67, 1.42) | 0.12 |

Abbreviations: AVF – arteriovenous fistula; CI – confidence interval; n – number.

^a^Surgical or radiological revision or dilatation of the AVF from or proximal to the anastomosis to the ipsilateral central vein, dilation of central venous stenosis, ligation of tributaries, superficialisation of AVF, thrombolysis or thrombectomy, ligation of fistula, salvage by distal reconstruction and interval ligation, others.

^b^Thrombolysis or thrombectomy.

^a^Surgical or radiological revision or dilatation of the AVF from or proximal to the anastomosis to the ipsilateral central vein, dilation of central venous stenosis, ligation of tributaries, superficialisation of AVF, ligation of fistula or salvage by distal reconstruction and interval ligation.

^d^Adjusted for differences in aspirin use (no aspirin, randomized to aspirin, open-label aspirin).
