# Supplementary figures and images for: Fish oil and aspirin effects on arteriovenous fistula function: Secondary outcomes of the randomised omega-3 fatty acids (Fish oils) and Aspirin in Vascular access OUtcomes in REnal Disease (FAVOURED) trial
Source: PLoS One. 2019 Mar 26;14(3):e0213274. doi: 10.1371/journal.pone.0213274 (PMC6435148; doi:10.1371/journal.pone.0213274)

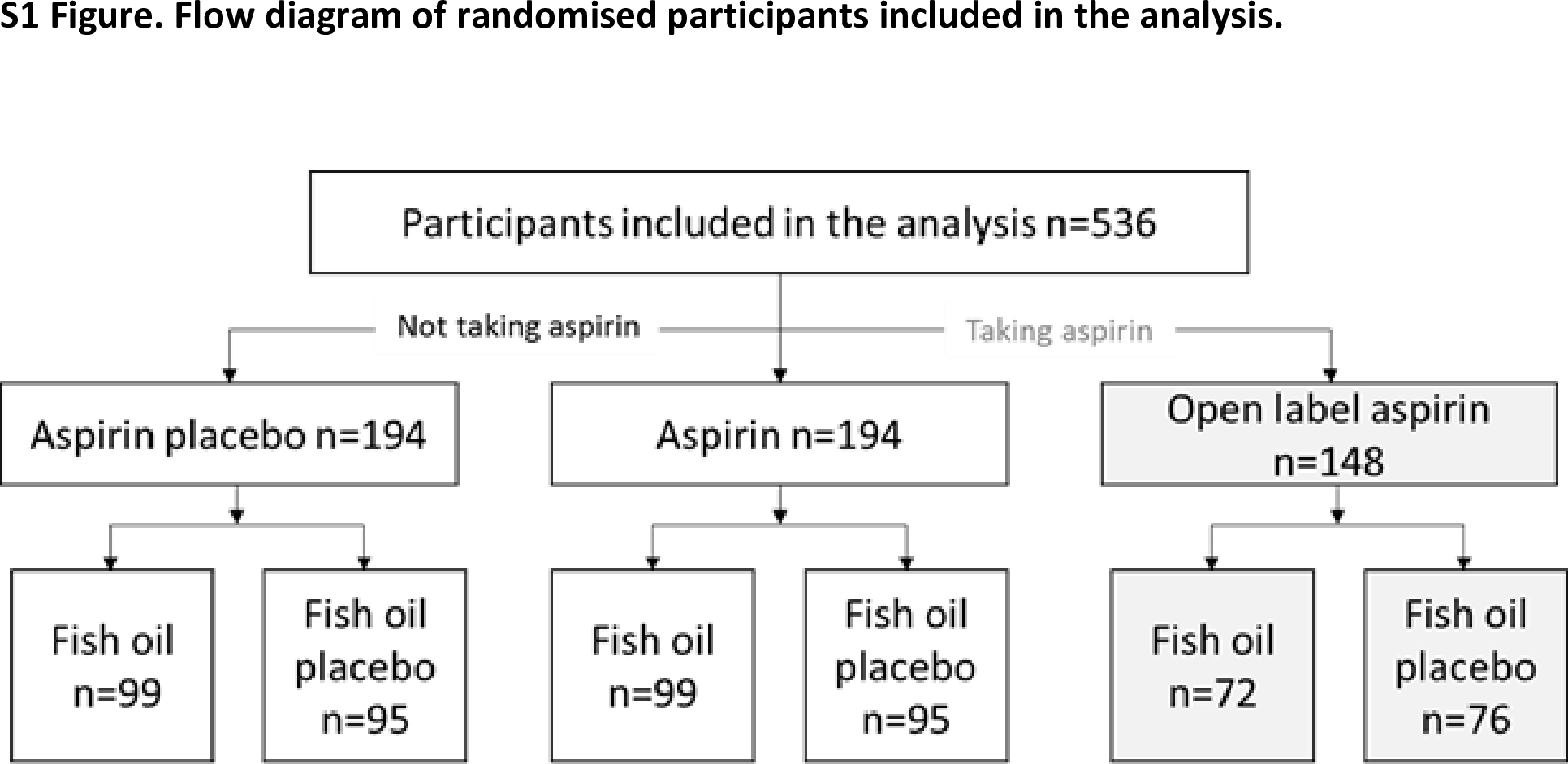

Supplement: S1 Fig — (TIF) [file pone.0213274.s006.tif]
